# Total Synthesis of (+)-Penicyclone A and Evaluation of Biological Activity Including Intermediate Compounds

**DOI:** 10.3390/ijms26146643

**Published:** 2025-07-11

**Authors:** Mirko Duvnjak, Gregor Talajić, Jurica Baranašić, Nea Baus Topić, Hana Čipčić Paljetak, Nikola Cindro

**Affiliations:** 1 Department of Chemistry, Faculty of Science, University of Zagreb, Horvatovac 102a, 10000 Zagreb, Croatia; mduvnjak@chem.pmf.hr (M.D.); gtalajic@chem.pmf.hr (G.T.); nea.baus@chem.pmf.hr (N.B.T.); 2 Ruđer Bošković Institute, Bijenička c. 54, 10000 Zagreb, Croatia; jurica.baranasic@irb.hr; 3 Center for Translational and Clinical Research, School of Medicine, University of Zagreb, Šalata 2, 10000 Zagreb, Croatia; hana.paljetak@mef.hr

**Keywords:** double Grignard reaction, fungus metabolite, natural product, polyketide, total synthesis

## Abstract

Penicyclone A is a polyketide compound with a unique and intriguing structure recently isolated from the fungus *Penicillium* sp. F23-2 during an OSMAC (one-strain-many-compounds) campaign. The compound demonstrated significant antimicrobial activity without exhibiting any cytotoxic effects, which prompted us to pursue total synthesis of the reported enantiomer. Upon completion of the synthesis, we observed that our synthetic compound lacked antimicrobial activity. Further analysis suggested that the natural product may have, in fact, been the opposite enantiomer to that reported. This observation led us to synthesize the antipodal enantiomer using our previously developed synthetic sequence and to evaluate the biological activity (via antibacterial and cytotoxicity assays) of both the final compound and the selected intermediates from both enantiomeric series.

## 1. Introduction

**Figure 1:**
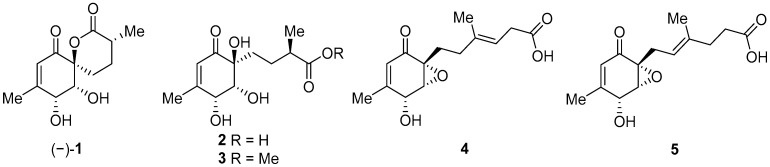
Molecular structures of penicyclones A–E (**1**–**5**).

Marine natural products derived from organisms such as sponges, corals, algae and bacteria often exhibit sought-after antimicrobial, anticancer and anti-inflammatory properties which make them potential candidates for further drug development [1,2]. Penicyclones are polyketide secondary metabolites isolated from the deep-sea-derived fungus *Penicillium* sp. F23-2. Five representatives of this class are known, penicyclones A–E (Figure 1), with their isolation and characterization reported in 2015 [3]. Other natural compounds have previously been isolated from *Penicillium* spp., such as cytotoxic non-ribosomal peptide synthetases (NRPSs), alkaloids (meleagrins and roquefortines) and diterpenes (conidiogenones) in a potato-based medium in static conditions [4]. Nitrogen-containing polyketides (sorbicillinoids) were discovered when the fungus was grown in an agitated peptone yeast glucose (PYG) medium [5]. Changing the microorganism cultivation conditions often results in the formation of new secondary metabolites [6,7,8], and thus penicyclone compounds were discovered using the one-strain-many-compounds (OSMAC) approach when the fungus was grown on a rice nutrient medium. The isolated compounds were structurally characterized, and their minimum inhibitory concentrations (MICs) were determined for several bacterial strains, including *S. aureus*, for which penicyclone A (**(−)-1**) had an MIC of 0.3 µg/mL, which was the lowest value among the tested compounds. Penicyclone A has a very rare and interesting spiro [5.5]lactone [9] motif in its structure, which, in addition to its pronounced biological activity, encouraged us to carry out its total synthesis [10]. We recently published a paper describing its synthesis; however, the obtained compound did not show the reported biological activity. The synthetic sample of (−)-penicyclone A and the naturally obtained sample were identical in terms of the proton and carbon NMR results, as well as their MS spectra. The only differences, in addition to its antimicrobial activity, were the direction and value of the optical rotation. In the course of the synthesis, we used SCXRD to confirm the stereochemistry at several points, including for the final compound bearing TMS groups. The presence of a heavier atom (Si) enhanced the precision of determination of the absolute configuration further. In addition, the data for single-crystal diffraction in the original isolation report had high deviation in the Flack parameter, which additionally indicated that the natural compound may have been a mixture of enantiomers or the antipodal enantiomer. Recently, it was shown that a certain number of compounds isolated from nature come in racemic form [11]. In order to investigate this, we prepared the enantiomer of the compound and tested its antimicrobial activity, as well as the antimicrobial activity and cytotoxicity of the synthetic intermediates.

## 2. Results and Discussion

### 2.1. Synthesis of (+)-Penicyclone A and Selected Derivatives

**Figure 2:**
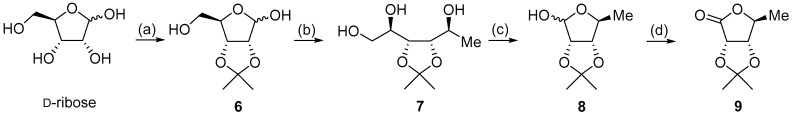
The preparation of l-deoxyribonolactone derivative **9**. Reaction conditions: (a) 2,2-dimethoxypropane (1.05 eq.), acetone, H_2_SO_4_ (0.3 mol%), 5 °C, 20 h (79%); (b) MeMgI (10 eq.), Et_2_O, 0 °C to RT, 3.5 h, then NH_4_Cl (38%); (c) NaIO_4_ (1.4 eq.), THF:H_2_O (1:1), 0 °C to RT, 15 min (97%); (d) TEMPO (2.4 mol%), NaOCl (2.6 eq.), KBr (1.8 eq.), NaHCO_3_ (1.5 eq.), EtOAc/H_2_O, 0 °C to RT, 1 h (91%).

The most logical approach to the synthesis of the antipodal enantiomer was to use the l-ribose derivative as the starting material instead of the d-ribose derivative in the reaction sequence that we developed for the chiral total synthesis of (−)-penicyclone A [10]. To our delight, it turns out that compound **9**, which is the enantiomer that we needed for synthesis, could be prepared from d-ribose in a modified short reaction sequence, as described by M. Kinoshita and coworkers [12] and K. P. Kaliappan and coworkers [13] and shown in Figure 2. In the first step, the *cis*-diol moiety of d-ribose was protected with the acetonide group, forming **6**, which was then reacted with methylmagnesium iodide, affording the triol **7**. Oxidative cleavage using sodium periodate formed the hemiacetal **8**, which was easily oxidized into the ribonolactone derivative **9** using a TEMPO/NaOCl/KBr oxidation system.

**Figure 3:**
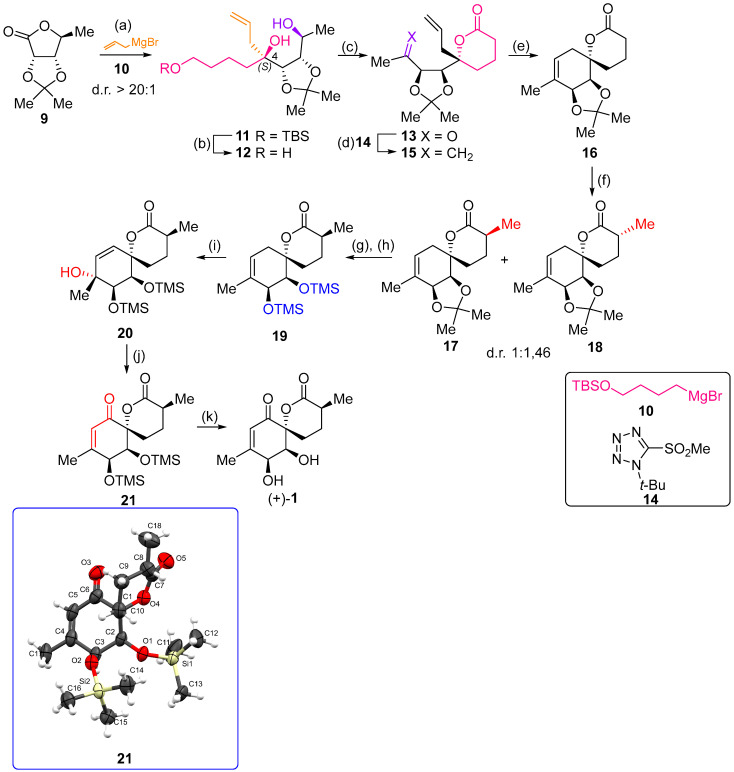
Synthesis of (+)-penicyclone A (**(+)-1**). Reaction conditions: (a) allylmagnesium bromide (1 eq.), Et_2_O/THF, −78 °C, 1.5 h then TBSO(CH_2_)_4_MgBr (**10**) (2 eq.), −78 °C to RT, 30 min; (b) TBAF (1.8 eq.), DCM/THF, RT, 20 h (48% over two steps); (c) TEMPO (0.2 eq.), PIDA (3.6 eq.), DCM, RT, 3 h then DMP (1.4 eq.), RT, 2.5 h (83%); (d) **13** (1.4 eq.), NaHMDS (1.3 eq.), THF, 0 °C to RT, 20 h (52%); (e) Grubbs–Hoveyda II (2 mol%), toluene, 200 mbar, 4 h (98%); (f) NaHMDS (1.01 eq.), THF, −78 °C, 50 min then MeI (2 eq.), −50 °C (2 h) to RT (30 min) (30% **17** and 44% **18**), (g) TFA, DCM/H_2_O, RT, 1.5 h; (h) HMDS (2.5 eq.), MeNO_2_, RT, 5 min (75% over two steps); (i) tetraphenylporphyrin (2.5 mol%), O_2_, hν, CDCl_3_, RT, 46 h then PPh_3_ (1.14 eq.), 5 min (41%), (j) PCC (12 mol%), PIDA (3 eq.), O_2_, DCM, RT, 18 h (34%), (k) TFA (3.65 eq.), MeOH, RT, 20 min (91%).

As shown in Figure 3, with compound **9** to hand at the gram scale, we started the synthesis of (+)-penicyclone A. The first step was the double Grignard reaction according to our previous method [10], the scope of which we are currently investigating. The reaction involves the two-stage addition of different Grignard reagents to the lactone, which results in diastereoselective formation of the chiral tertiary alcohol **11**, in contrast to the regular Grignard reaction, in which esters are converted into achiral tertiary alcohols bearing two identical substituents. In this case, compound **9** was first treated with allylmagnesium bromide, which was followed by the addition of Grignard reagent **10** derived from THF in two steps. This resulted in the highly diastereoselective formation of compound **11**, and no trace of the (4*R*) diastereomer was detected. The protected form of tertiary alcohol **11** was first treated with TBAF to remove the TBS and also to simplify the purification step, as the side product in partial addition is decomposed using TBAF and enables simple chromatographic separation of the product over two steps. In terms of the yield (in comparison to that in our previously reported synthesis of (−)-penicyclone A), the diastereoselective Grignard reaction followed by TBS deprotection using TBAF resulted in similar outcomes, affording triol **12** at a 48% yield over two steps. The next step was a remarkable oxidation/cyclization process in which three hydroxyl groups in the molecule took part. TEMPO-oxidation of the primary hydroxyl group in **12** afforded a hemiacetal with a tertiary OH group. Since the hemiacetal was the most reactive in this sequence, it was further oxidized into the corresponding lactone, leaving the secondary OH group to be partially oxidized into ketone [14]. To accelerate this process, after establishing that there was no starting material, DMP was added to complete the oxidation into ketolactone **13**. This complex transformation is based on the different reactivities of the OH groups, as well as the kinetics of oxidation of the intermediates. Primary alcohol is oxidized faster than secondary alcohol and quickly forms the corresponding aldehyde. Since aldehyde can form a six-membered ring with the tertiary alcohol, which is not oxidized in these conditions, the hemiacetal is formed quickly. The next step was transformation of the ketone into methylidene **15** using a modified Julia–Kocienski procedure using sulfone **14** [15] since all other methods failed to provide the corresponding bismethylene precursor **15**. This is most likely due to steric hindrance of the neighboring lactone and isopropylidene rings, making the conventionally used bulkier reagents incapable of carrying out the olefination reaction. In the next step, ring-closing metathesis using bismethylene **15** provided smooth access to spirolactone **16**, which was methylated using a simple alpha-alkylation approach, affording diastereomers **17** and **18** at a 1:1.46 ratio. Compound **17** was converted into (+)-penicyclone A in five steps. Swapping the protective groups from acetonide **17** with bis-TMS and photooxygenation of compound **19**, followed by oxidative rearrangement of tertiary allylic alcohol **20**, introduced the enone moiety, as discussed in the previous report [10], since all other methods led to allylic oxidation for more activated carbons. Removal of the silyl protective groups was the final step that furnished (+)-penicyclone A.

The NMR and HRMS spectra of (+)-penicyclone A were identical to those from the report by Li’s group [3], as well as those of our sample obtained in the previously described synthesis of the other enantiomer (denoted as (−)-penicyclone A) [10].

The value of optical rotation reported by Li’s group was ${\left[\alpha \right]}_{D}^{23}$ + 72.8 (*c* 0.10, MeOH), while the optical rotation measured for the previously obtained synthetic (−)-penicyclone A was ${\left[\alpha \right]}_{D}^{23}$ − 198.0 (*c* 0.10, MeOH). The optical rotation for the synthetic sample was also determined to be ${\left[\alpha \right]}_{D}^{23}$ − 206.0 (*c* 0.10, CHCl_3_) in chloroform. For the enantiomer prepared in the scope of this paper ((+)-penicyclone A), we measured an optical rotation of ${\left[\alpha \right]}_{D}^{23}$ + 201.2 (*c* 0.24 CHCl_3_), which is of a similar value but of a different sign compared to the value for the previously obtained synthetic sample.

The crystal structures of the synthetic intermediates **21** and the previously obtained ***ent*-21** were determined through SCXRD. The Flack parameters for compound **21** and ***ent*-21** were refined to 0.03(4) (see the Supplementary Materials for detailed crystallographic data) and 0.002(13), respectively. The high certainty of the Flack parameters and the similar values for optical rotation with different signs confirm that the final product **(+)-1** is the antipodal enantiomer compared to our previously synthesized specimen [10].

**Figure 4:**
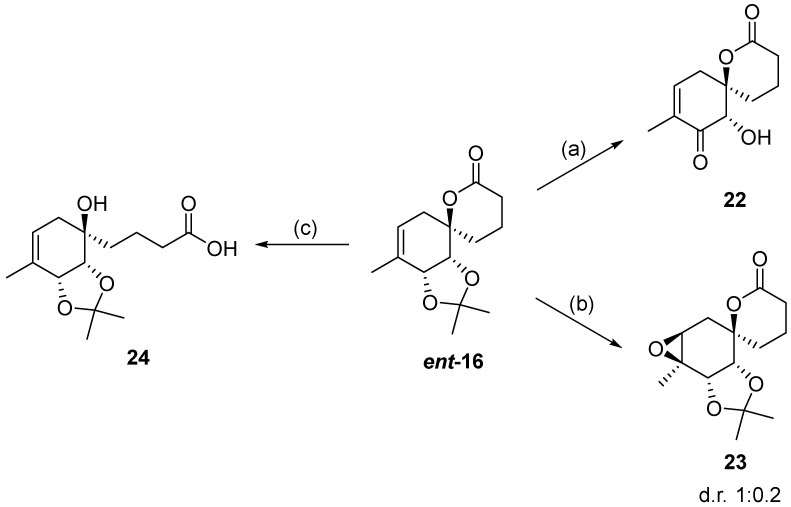
Synthesis of derivatives **22**–**24** from spirolactone ***ent*-16**. Reaction conditions: (a) SeO_2_ (2.2 eq.), KH_2_PO_4_ (3.2 eq.), MeNO_2_, 60 °C, 2 h (30%); (b) *m*-CPBA (1.2 eq.), NaHCO_3_ (1.8 eq.), DCM, RT, 24 h (30%); (c) LiOH × H_2_O (4.5 eq.), MeOH/H_2_O, RT, 16 h (quant.).

Additionally, several derivatives of the spirolactone intermediate ***ent*-16** were prepared, as shown in Figure 4. Allylic oxidation conditions using SeO_2_/KH_2_PO_4_ in nitromethane [16] resulted in acetonide cleavage and alcohol oxidation, affording compound **22**. Epoxidation of ***ent*-16** with *m*-CPBA gave an inseparable mixture of diastereomers **23** at a 1:0.2 ratio. The diastereomeric ratio was determined by comparing the ^1^H NMR integrals of the signals from the major and minor diastereomers. Lactone hydrolysis using LiOH gave the corresponding carboxylic acid **24**.

### 2.2. Biological Evaluation

With (+)-penicyclone A in hand, the potential pharmacological activities of the prepared compounds were evaluated. The synthetic intermediates obtained from the (+)-penicyclone A synthetic sequence, as well as the enantiomers obtained from the previously reported synthetic sequence through which (−)-penicyclone A was prepared, were evaluated. The minimum inhibitory concentrations (MICs) were determined for *S. aureus* (ATCC 29213), *E. faecalis* (ATCC 29212), *M. catarrhalis* (ATCC 23246) and *E. coli* (TolC-Tn10). Cytotoxicity was evaluated in ovarian (MES-OV, ATCC CRL-3272) and breast (MDA-MB-468, ATCC HTB-132) cancer lines (Table 1).

## 3. Materials and Methods

Please see the Supplementary Materials file for general and detailed experimental procedures, synthetic sequence (Figure S1), detailed crystallographic data (Figures S2 and S3 and Tables S1 and S2), ^1^H and ^13^C NMR spectra (Figures S4–S40) and HRMS spectra (Figures S41–S55).

### 3.1. Chemicals

All solvents and reagents were purchased from Sigma-Aldrich (St. Louis, MO, USA), Carbolution (St. Ingbert, Germany) or Merck KGaA (Darmstadt, Germany). All reactions were carried out in dry solvents under an inert argon atmosphere unless otherwise stated. Dichloromethane (DCM) and methanol (MeOH) were dried using 4 Å and 3 Å molecular sieves, respectively. Tetrahydrofuran (THF), diethyl ether (Et_2_O) and toluene were distilled over sodium prior to their use.

### 3.2. Methods

#### 3.2.1. The In Vitro Cytotoxicity Assay

The human ovarian carcinoma MES-OV (ATCC CRL-3272) and human breast cancer MDA-MB-468 (ATCC HTB-132) cell lines were obtained from the American Type Culture Collection (ATCC) (Manassas, VA, USA). The MES-OV cells were grown in McCoy’s 5 A medium supplemented with 10% fetal bovine serum (FBS) (Gibco BRL Life Technologies, Carlsbad, CA, USA), while the MDA-MB-468 cells were grown in Dulbecco’s Modified Eagle Medium (DMEM) supplemented with 10% FBS. The cells were cultured in a humidified atmosphere of 5% CO_2_ at 37 °C.

The cytotoxicity was determined using the Alamar Blue colorimetric assay [17] (Sigma-Aldrich, St. Louis, MO, USA). Briefly, the cells were seeded into a 96-well plate. Proportions of 5 × 10^3^ MES-OV cells and 1 × 10^4^ MDA-MB-468 cells per well were seeded into 180 µL of the appropriate medium. The next day, the cells were treated with 0.01, 0.1, 1 and 10 µM of the intermediate compounds. After 72 h, the medium was discarded, and Alamar Blue solution was added. After 4 h of incubation, the fluorescence of resorufin was measured using a Tecan Infinite 200 Pro microplate reader (Tecan, Männedorf, Switzerland). To determine the IC_50_ values, nonlinear regression was used in GraphPad Prism (v 8.4.3, GraphPad Software, Boston, MA, USA).

#### 3.2.2. The In Vitro Antibacterial Activity Assay

The organisms tested represent relevant Gram-positive (*Staphylococcus aureus* and *Enterococcus faecalis*) and Gram-negative (*Escherichia coli* and *Moraxella catarrhalis*) organisms. The *E. coli* TolC-Tn10 strain has an impaired multidrug resistance (MDR) AcrAB-TolC efflux system due to disruption of the tolC gene, resulting in hypersensitivity to multiple antibiotics compared to that in wild-type strains.

The MICs were assessed using the broth microdilution method in accordance with the CLSI guidelines [18]. The tested compounds were initially dissolved in dimethyl sulfoxide (DMSO) at a concentration of 10 mg mL^–1^. Azithromycin and ciprofloxacin served as the reference controls. Serial two-fold dilutions of the compounds were prepared in Mueller–Hinton broth using 96-well microplates, yielding final concentrations ranging from 0.25 to 128 μg mL^−1^. The bacterial strains were cultured on appropriate agar plates (Becton Dickinson, Franklin Lakes, NJ, USA): Columbia agar with 5% sheep blood for *E. fecalis* and *M. catarrhalis*, while *S. aureus* and *E. coli* were grown on Mueller–Hinton agar. The inocula were prepared through direct colony suspension, and microtiter plates were inoculated with approximately 5 × 10^5^ CFU/mL (5 × 10^4^ CFU/well). The MICs were determined through visual inspection following 18–22 h of incubation at 37 °C in ambient air.

## 4. Conclusions

In the original report describing the isolation and structural elucidation of penicyclone A, SCXRD was employed to assign the absolute configuration. However, due to the absence of heavier atoms in the structure, the Flack parameter derived from the data indicated uncertainty regarding its absolute stereochemistry. During our synthetic efforts, we were able to confirm the absolute configuration at several key positions using silylated intermediates. Following the successful total synthesis of both enantiomers of penicyclone A, we evaluated their biological activity, including their potential antimicrobial and antiproliferative effects, as well as that of selected intermediates from both enantiomeric series. Unfortunately, none of the tested compounds displayed significant biological activity, indicating that they are unlikely to represent a promising new class of antimicrobial agents.

## Figures and Tables

**Table 1:** Evaluation of pharmacological activities of selected prepared compounds.

Compound	MIC (µg/mL)				IC_50_ (µM)	
	*S. aureus*	*E. faecalis*	*M*. *catarrhalis*	*E. coli*	MES-OV	MDA-MB-468
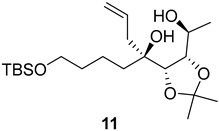	>128	>128	>128	>128	>10	>10
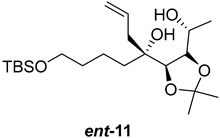	>128	>128	>128	>128	>10	>10
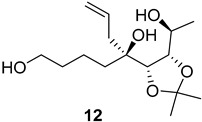	>128	>128	>128	>128	>10	>10
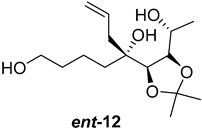	>128	>128	>128	>128	>10	>10
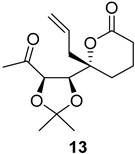	>128	>128	>128	>128	>10	>10
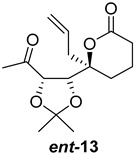	>128	>128	>128	>128	>10	>10
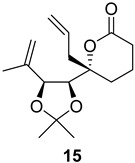	>128	>128	>128	>128	>10	>10
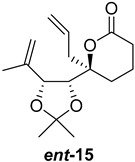	>128	>128	>128	>128	>10	>10
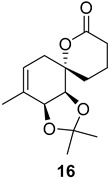	>128	>128	>128	>128	>10	>10
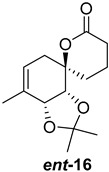	>128	>128	>128	>128	>10	>10
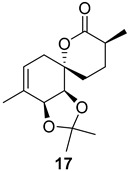	>128	>128	>128	>128	>10	>10
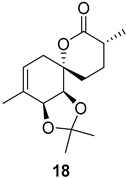	>128	>128	>128	>128	>10	>10
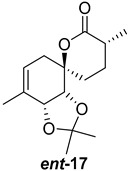	>128	>128	>128	>128	>10	>10
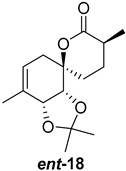	>128	>128	>128	>128	>10	>10
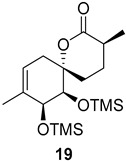	>128	>128	>128	>128	>10	>10
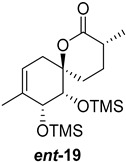	>128	>128	>128	>128	>10	>10
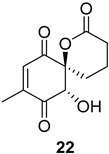	>128	>128	>128	>128	>10	>10
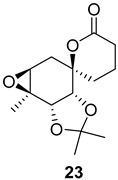	>128	>128	>128	>128	>10	>10
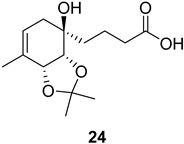	>128	>128	>128	>128	>10	>10
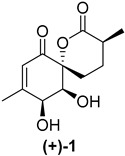	>128	>128	>128	>128	>10	>10
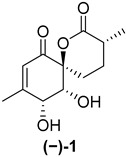	>128	>128	>128	>128	>10	>10

## Data Availability

CCDC 2449626 contains the supplementary crystallographic data for this paper. These data can be obtained free of charge at https://www.ccdc.cam.ac.uk/structures/ (accessed on 7 July 2025) (or from the Cambridge Crystallographic Data Centre, 12, Union Road, Cambridge CB2 1EZ, UK; fax: +44-1223-336033).

## Supplementary Materials

The following supporting information can be downloaded at https://www.mdpi.com/article/10.3390/ijms26146643/s1. References [19,20,21,22,23,24,25,26,27] are cited in the Supplementary Materials.

## Abbreviations

The following abbreviations are used in this manuscript:

DMP	Dess–Martin periodinane
HRMS	High-resolution mass spectrometry
MIC	Minimum inhibitory concentration
NMR	Nuclear magnetic resonance
NRPS	Non-ribosomal peptide synthetase
OSMAC	One-strain-many-compounds
PYG	Peptone yeast glucose
SCXRD	Single-crystal X-ray diffraction
TBAF	Tetrabutylammonium fluoride
TBS	*tert*-butyldimethylsilyl protective group
TEMPO	(2,2,6,6-Tetramethylpiperidin-1-yl)oxyl
THF	Tetrahydrofuran
TMS	Trimethylsilyl protective group
