# Supplementary material for: Total Synthesis of (+)-Penicyclone A and Evaluation of Biological Activity Including Intermediate Compounds
Source: Int J Mol Sci. 2025 Jul 11;26(14):6643. doi: 10.3390/ijms26146643 (PMC12295702; doi:10.3390/ijms26146643)
Supplement: Supplementary file 1 [file ijms-26-06643-s001.zip › ijms-3722221-supplementary.pdf]

# Supplementary Materials

## Total Synthesis of (+)-Penicyclone A and Evaluation of Biological Activity of Intermediate Compounds

Mirko Duvnjak,[a‡] Gregor Talajić,[a‡] Jurica Baranašić,[b] Nea Baus Topić,[a] Hana Čipčić Paljetak,[c] Nikola Cindro[a*]

[a] Department of Chemistry, Faculty of Science, University of Zagreb, Horvatovac 102a, 10000 Zagreb, Croatia

[b] Ruđer Bošković Institute, Bijenička c. 54, Zagreb 10000, Croatia

[c] Center for Translational and Clinical Research, Croatian Center of Excellence for Reproductive and Regenerative Medicine, School of Medicine, University of Zagreb, 10000 Zagreb, Croatia

‡These authors contributed equally as co-first authors.

[*] e-mail: ncindro.chem@pmf.hr

# TABLE OF CONTENTS

1. Synthesis of (+)-penicyclone A ........ S2
   1.1. General methods and materials ........ S3
   1.2. Preparation of L-ribonolactone derivative **9**' ........ S4
   1.3. Preparation of (+)-penicyclone A ........ S7
   1.4. Preparation of synthetic derivatives **22** – **24** ........ S19
2. NMR spectra ........ S22
3. HRMS spectra ........ S59

# 1. Synthesis of (+)-penicyclone A

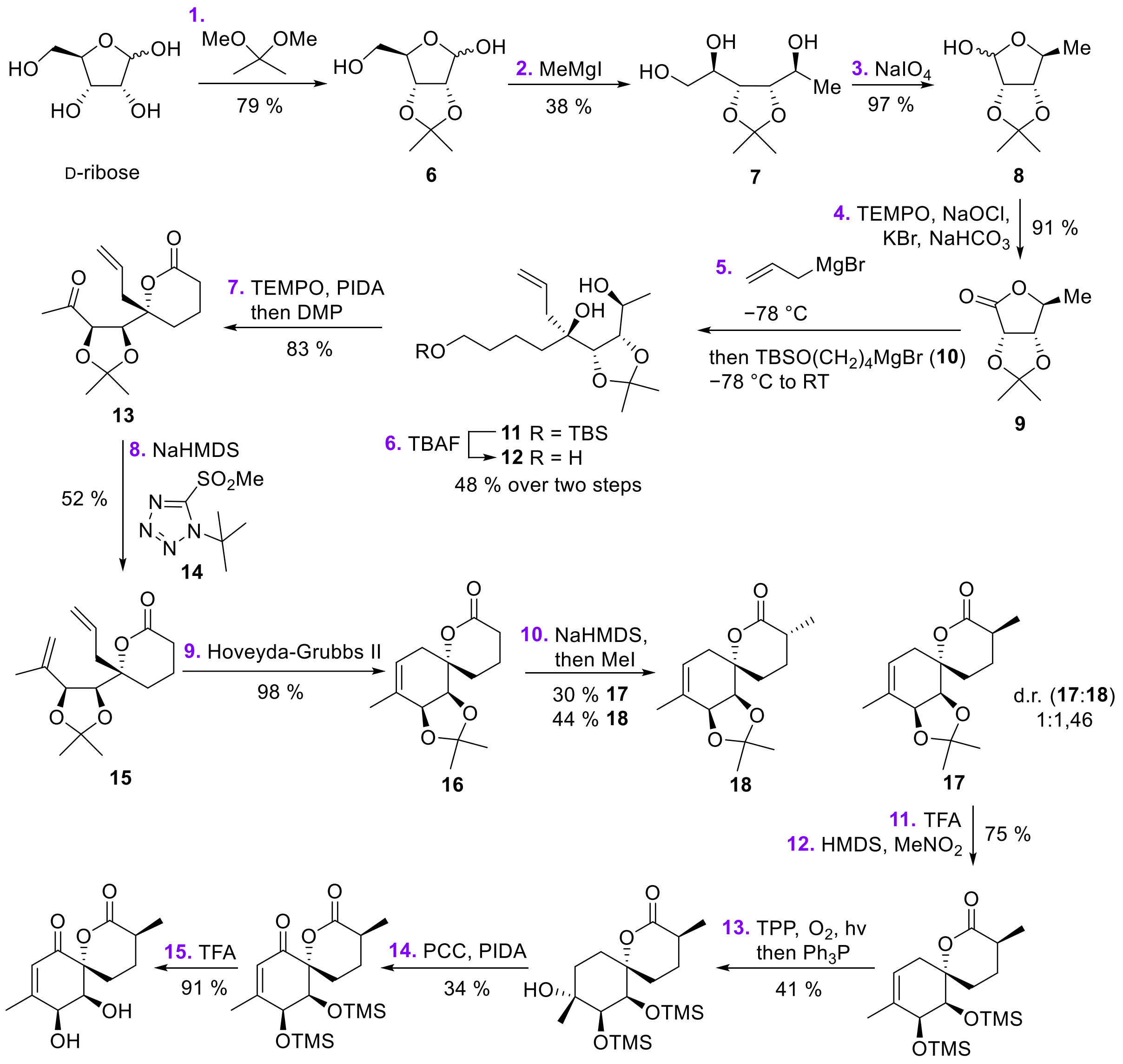


**Figure S1**. Total synthesis of (+)-penicyclone A from D-ribose.

## 1.1. General methods and materials

All solvents and reagents were purchased from Sigma-Aldrich, Carbolution or Merck. All reactions were carried out in dry solvents under an inert argon atmosphere unless otherwise stated. Dichloromethane (DCM) and methanol (MeOH) were dried using 4 Å and 3 Å molecular sieves, respectively. Tetrahydrofuran (THF), diethyl ether ($Et_2O$) and toluene were distilled over sodium prior to use. Analytical Thin Layer Chromatography (TLC) was performed on silica gel 60 F254 precoated aluminum sheets with a fluorescent indicator (0.2 mm layer; Merck, Darmstadt, Germany), the components were detected under a UV lamp ($\lambda$ = 254 nm) and the plates were developed using $KMnO_4$ and heat. Silica gel chromatography was performed using Merck silica gel (60, particle size 0.040 – 0.063 mm). $^1$H and $^{13}$C NMR spectra were acquired by means of a Bruker Ascend 400 MHz or 600 MHz spectrometer (Bruker, Carlsruhe, Germany). Solutions of compounds were prepared in $CDCl_3$ or DMSO-$d_6$ with tetramethylsilane (TMS) as the internal standard. Chemical shifts ($\delta$) are given in ppm and are referenced to TMS ($^1$H NMR 0 ppm) or solvent signals ($CHCl_3$: $^1$H NMR $\delta$ = 7.26 ppm, $^{13}$C NMR $\delta$ = 77.23 ppm; DMSO: $^1$H NMR $\delta$ = 2.50 ppm, $^{13}$C NMR $\delta$ = 39.52 ppm). High-resolution mass spectra (HRMS) were recorded on a Thermo Fisher Scientific Q Exactive ESI Orbitrap mass spectrometer. Optical rotations were measured on a Schmidt Haensch Polartronic NH8 polarimeter using a 10 cm cuvette.

Single crystals of compound **21** were obtained by recrystallization from $Et_2O$. Single crystal X-ray diffraction (SCXRD) data were obtained using a Rigaku XtaLAB Synergy-S diffractometer equipped with a Dualflex source (CuKα radiation, $\lambda$ = 1.54184 Å) and HyPix detector, using ω-scans. The crystals were kept at 298 K during data collection. Data were prepared using the CrysAlis program package.[1] The structures were solved with dual space methods using SHELXT.[2] The refinement procedure by full-matrix least-squares methods based on $F^2$ values against all reflections included anisotropic displacement parameters for all non-H atoms. Hydrogen atoms bound to carbon atoms were placed in geometrically idealized positions and refined using the riding model with $U_{iso} = 1.2U_{eq}$ of the connected carbon atom or as ideal $CH_3$ groups with $U_{iso} = 1.5U_{eq}$. Hydrogen atoms attached to heteroatoms were located in the difference Fourier maps at the final stages of the refinement procedure. All refinements were performed using SHELXL.[3] The SHELX programs operated within the Olex2 suite.[4] Geometrical calculations and molecular graphics were done with Platon[5] and Mercury.[6] CCDC **2449626** contains the supplementary crystallographic data for this paper. These data can be obtained free of charge via http://www.ccdc.cam.ac.uk/conts/retrieving.html (or from the Cambridge Crystallographic Data Centre, 12, Union Road, Cambridge CB2 1EZ, UK; fax: +44 1223 336033).

1.2. **Preparation of L-ribonolactone derivative 9**[7,8]

**Preparation of acetonide 6**

((3a*R*,6*R*,6a*R*)-6-(hydroxymethyl)-2,2-dimethyltetrahydrofuro[3,4-d][1,3]dioxol-4-ol)

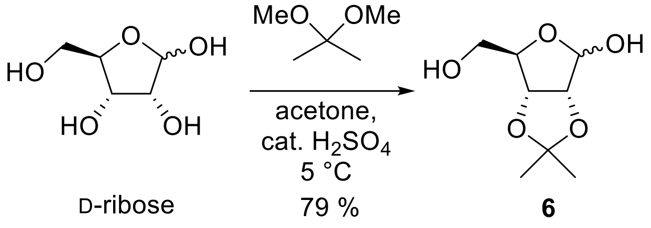


D-ribose (10.0 g, 66.6 mmol, 1 eq.) was suspended in dry acetone (200 mL), 2,2-dimethoxypropane (8.6 mL, 70.2 mmol, 1.05 eq.) and concentrated sulphuric acid (10.0 µL, 0.187 mmol, 0.3 mol%) were added and the reaction mixture was stirred at 5 °C for 20 h. The reaction was quenched by addition of $NaHCO_3$ (1 g), the mixture was filtered and concentrated *in vacuo*. 13.89 g of the crude product were purified by column chromatography (10 % MeOH in DCM). 9.99 g (79 %) of pure compound **6** were obtained as a slightly yellow oil. The $^1$H NMR spectrum is in agreement with previously reported spectra.[9]

**$^1$H NMR** (400 MHz, $CDCl_3$) $\delta$/ppm: 5.43 (d, $J$ = 6.4 Hz, 1H), 4.85 (d, $J$ = 5.9 Hz, 1H), 4.60 (d, $J$ = 5.9 Hz, 1H), 4.42 (t, $J$ = 2.7 Hz, 1H), 4.39 (d, $J$ = 6.4 Hz, 1H), 3.80 – 3.69 (m, 2H), 3.33 (dd, $J_1$ = 7.3 Hz, $J_2$ = 3.1 Hz, 1H), 1.49 (s, 3H), 1.33 (s, 3H)

**Preparation of allitol 7**

((*R*)-1-((4*R*,5*S*)-5-((*S*)-1-hydroxyethyl)-2,2-dimethyl-1,3-dioxolan-4-yl)ethane-1,2-diol)

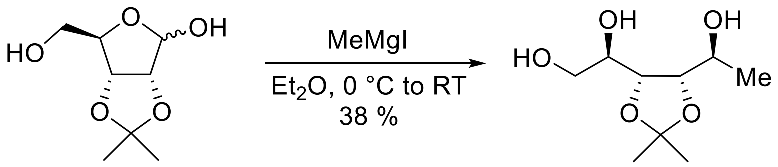


A fresh solution of MeMgI was prepared from magnesium turnings (12.8 g, 0.527 mol, 10 eq.) and MeI (36 mL, 0,578 mol, 11 eq.) in dry $Et_2O$ (400 mL). The mixture was cooled to 0 °C and a solution of **6** (9.99 g, 52.6 mmol, 1 eq.) in dry $Et_2O$ (200 mL) was added dropwise. The reaction mixture was warmed to RT and stirred for 3.5 h after which a saturated solution of $NH_4Cl$ (75 mL) was slowly added at 0 °C. The mixture was transferred to a separatory funnel, diluted with EtOAc (500 mL) and the phases were separated. The aqueous layer was extracted with EtOAc (2×300 mL + 3×50 mL). The organic extracts were combined, washed with brine, dried over anhydrous $Na_2SO_4$, filtered and concentrated *in vacuo*. 6.9 g of the crude product

were purified by column chromatography (EtOAc). 4.07 g (38 %) of pure compound **7** were obtained as a colourless oil and 1.23 g of the starting material were recovered. The $^{1}$H and $^{13}$C NMR spectra are in agreement with previously reported spectra.[10]

**$^{1}$H NMR** (400 MHz, $CDCl_3$) $\delta$/ppm: 4.11 (dd, $J_1$ = 9.2 Hz, $J_2$ = 5.3 Hz, 1H), 4.05 – 3.92 (m, 1H), 3.90 – 3.85 (m, 2H), 3.74 – 3.69 (m, 1H), 1.38 (s, 3H), 1.34 (d, $J$ = 6.0 Hz, 3H), 1.34 (s, 3H)

**$^{13}$C NMR** (100 MHz, $CDCl_3$) $\delta$/ppm: 108.9, 81.9, 77.7, 69.7, 66.2, 64.6, 28.2, 25.6, 20.9

**<u>Preparation of deoxy-L-ribofuranose 8</u>**

((3a*S*,6*S*,6a*S*)-2,2,6-trimethyltetrahydrofuro[3,4-d][1,3]dioxol-4-ol)

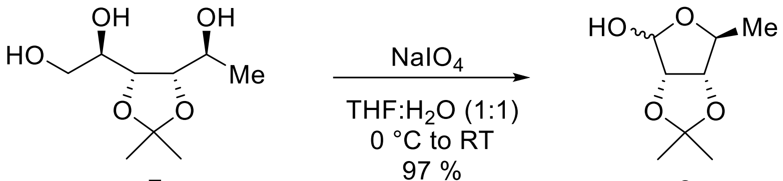


**7** **8**

A solution of **7** (4.07 g, 19.7 mmol, 1 eq.) in a THF:$H_2O$ (1:1) mixture (60 mL) was cooled to 0 °C and with mechanical stirring $NaIO_4$ (5.90 g, 27.6 mmol, 1.4 eq.) was added in small portions. The reaction mixture was stirred for 10 min at 0 °C, warmed to RT and stirred for an additional 5 minutes. The reaction was quenched by addition of a saturated $Na_2S_2O_3$ solution (100 mL), the contents were transferred to a separatory funnel and diluted with DCM (100 mL). The phases were separated and the aqueous layer was extracted with DCM (4×25 mL). The organic extracts were combined, dried over anhydrous $Na_2SO_4$, filtered and concentrated *in vacuo*. 3.33 g (97 %) of pure compound **8** were obtained as a colourless oil and used in the next step without further purification. $^{1}$H and $^{13}$C NMR spectra are in agreement with previously reported spectra.[10]

**$^{1}$H NMR** (400 MHz, $CDCl_3$) $\delta$/ppm: 5.43 (d, $J$ = 2.5 Hz, 1H), 4.68 (d, $J$ = 5.9 Hz, 1H), 4.56 (dd, $J_1$ = 5.9 Hz, $J_2$ = 0.8 Hz, 1H), 4.42 – 4.34 (m, 1H), 2.62 (d, $J$ = 2.5 Hz, 1H), 1.48 (s, 3H), 1.36 (d, $J$ = 7.0 Hz, 3H), 1.32 (s, 3H)

**$^{13}$C NMR** (100 MHz, $CDCl_3$) $\delta$/ppm: 103.6, 86.8, 85.8, 83.7, 26.7, 25.2, 22.0

**Preparation of L-ribonolactone derivative 9**

((3a*S*,6*S*,6a*S*)-2,2,6-trimethyldihydrofuro[3,4-d][1,3]dioxol-4(3aH)-one)

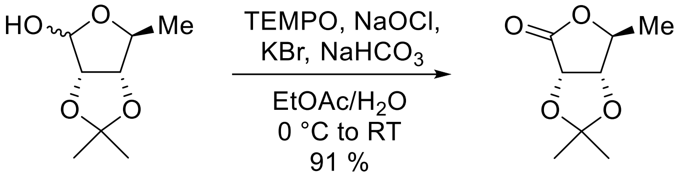


**8** **9**

To a solution of **8** (5.4 g, 31.0 mmol, 1 eq.) in EtOAc (170 mL) were added KBr (6.67 g, 56.0 mmol, 1.8 eq.), $NaHCO_3$ (3.90 g, 46.4 mmol, 1.5 eq.), TEMPO (121 mg, 0.744 mmol, 2.4 mol%) and water (50 mL). The biphasic mixture was cooled to 0 °C and with mechanical stirring a solution of NaOCl (*w*/*v* 13 %) (38 mL, 80.6 mmol, 2.6 eq.) was added over a course of 15 min. The reaction mixture was warmed to RT and stirred for 1 h. The reaction was quenched by addition of a saturated solution of $Na_2S_2O_3$ (5 mL), the contents were transferred to a separatory funnel and the phases were separated. The aqueous layer was extracted with EtOAc (2×50 mL), the organic extracts were combined, washed with brined, dried over anhydrous $Na_2SO_4$, filtered and concentrated *in vacuo*. 5.36 g of the crude product were purified by column chromatography (50 % to 100 % $Et_2O$ in hexanes). 4.85 g (91 %) of pure compound **9** were obtained as a colourless oil. $^1H$ and $^{13}C$ NMR spectra are in agreement with previously reported spectra.[8]

**$^1$H NMR** (400 MHz, $CDCl_3$) $\delta$/ppm: 4.79 (d, $J$ = 5.6 Hz, 1H), 4.70 (q, $J$ = 6.9 Hz, 1H), 4.51 (d, $J$ = 5.6 Hz, 1H), 1.49 (s, 3H), 1.39 (d, $J$ = 6.9 Hz, 3H), 1.39 (s, 3H)

**$^{13}$C NMR** (100 MHz, $CDCl_3$) $\delta$/ppm: 174.0, 114.2, 80.6, 79.2, 75.0, 27.0, 25.9, 19.9

**HRMS** (ESI) m/z $[M + H]^+$ calc. ($C_8H_{13}O_4^+$) 173.0769, found 173.0808

$R_f$ = 0.24 ($Et_2O$:Hex 1:1, $KMnO_4$), $\alpha_D^{23}$ +78.0 (*c* 1.00 $CHCl_3$)

### 1.3. Preparation of (+)-penicyclone A

**Preparation of tertiary alcohol 11**

((*S*)-8-((tert-butyldimethylsilyl)oxy)-4-((4*S*,5*S*)-5-((*S*)-1-hydroxyethyl)-2,2-dimethyl-1,3-dioxolan-4-yl)oct-1-en-4-ol)

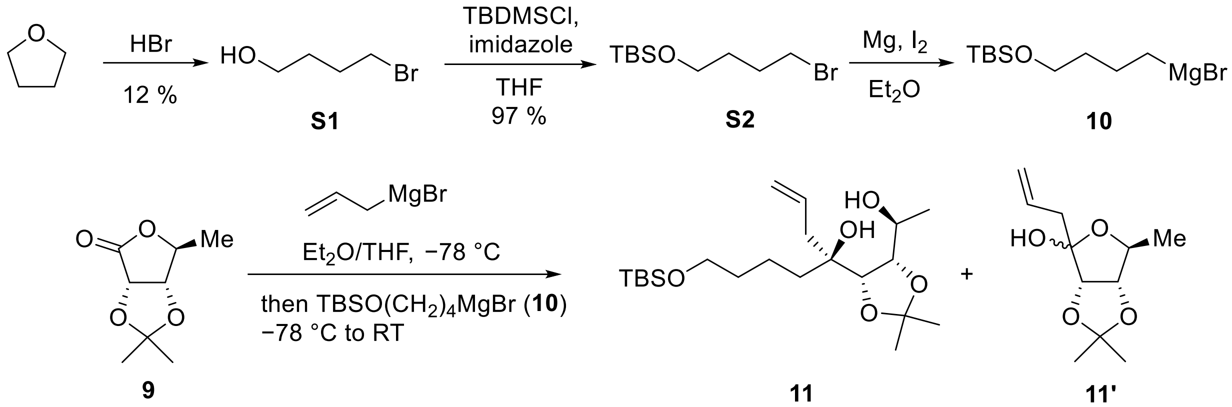


**Compound S1:** A 47 % solution of HBr (36 mL, 0.66 mol, 1.06 eq.) was added dropwise over 45 min to refluxing THF (50 mL, 0.62 mol, 1 eq.) and refluxing was continued for 2 h. The mixture was cooled to 0 °C, water (50 mL) and $Et_2O$ (75 mL) were added, the contents transferred to a separatory funnel and the phases separated. The aqueous layer was extracted with $Et_2O$ (2×30 mL), the combined organic extracts were washed with brine (15 mL), dried over anhydrous $Na_2SO_4$, filtered and concentrated *in vacuo*. Due to the volatility of the product, the solvent was not fully evaporated. 11.34 g (12 %) of **S1** were obtained as a viscous colourless liquid and used in the next step without further purification. $^1H$ NMR spectrum is in agreement with previously reported spectra.[11]

**$^1$H NMR** (400 MHz, $CDCl_3$) $\delta$/ppm: 3.69 (t, $J$ = 6.3 Hz, 2H), 3.46 (t, $J$ = 6.7 Hz, 2H), 2.01–1.94 (m, 2H), 1.76–1.70 (m, 2H)

**Compound S2:** TBDMSCl (12.23 g, 81.1 mmol, 1.1 eq.) was added in small portions to a solution of 1-bromobutanol (11.34 g, 74.1 mmol, 1 eq.) and imidazole (7.57 g, 111 mmol, 1.5 eq.) in dry THF (175 mL). The reaction mixture was stirred at RT for 2 h, concentrated *in vacuo*, $Et_2O$ (150 mL) was added, the contents were transferred to a separatory funnel and washed with a saturated solution of $NaHCO_3$ (2×25 mL). The aqueous layer was extracted with $Et_2O$ (50 mL), the combined organic extracts were washed with brine (2×25 mL), dried over anhydrous $Na_2SO_4$, filtered and concentrated *in vacuo*. Due to the volatility of the product, the solvent was not fully evaporated. The crude product was purified by a short column

chromatography (5 % EtOAc in Hex). 19.2 g (97%) of pure **S2** were obtained as a viscous colourless liquid. $^{1}$H NMR spectrum is in agreement with previously reported spectra.[11]

**$^{1}$H NMR** (400 MHz, $CDCl_3$) $\delta$/ppm: 3.64 (t, $J$ = 6.1 Hz, 2H), 3.45 (t, $J$ = 6.8 Hz, 2H), 1.98–1.90 (m, 2H), 1.69–1.52 (m, 2H), 0.89 (s, 9H), 0.05 (s, 6H)

**Compound 10:** To a suspension of magnesium turnings (1.38 g, 56.7 mmol, 1.06 eq.) in dry $Et_2O$ (25 mL) was added $I_2$ (1 mg) then **S2** (14.3 g, 53.5 mmol, 1 eq.) dropwise over 5 min. The mixture refluxes without external heating. After complete addition, refluxing was continued for 1 h. The reaction mixture was cooled and immediately used in the next step.

To a solution of **9** (4.60 g, 26.7 mmol, 1 eq.) in dry $Et_2O$ (160 mL) and dry THF (20 mL) at −78 °C under strong stirring was added a solution of allylmagnesium bromide ($c$ = 1.0 M in $Et_2O$) (26.7 mL, 26.7 mmol, 1 eq.) dropwise over a course of 50 min. The mixture was stirred at −78 °C for 35 min, then a freshly prepared solution of **10** (53.5 mmol, 2 eq.) was added over 10 min. The reaction mixture was warmed to RT and stirred for 30 min. The reaction was quenched by addition of a saturated solution of $NH_4Cl$ (90 mL), the contents were transferred to a separatory funnel, water (15 mL) was added, and the layers were separated. The aqueous layer was extracted with $Et_2O$ (2×50 mL), the combined organic extracts were washed with brine (50 mL), dried over anhydrous $Na_2SO_4$, filtered and concentrated *in vacuo*. 14.53 g of the crude product were purified by flash column chromatography (15 % to 30 % EtOAc in Hex). 6.59 g of **11** containing the monoadduct **11’** (ca. 10 mol %) were obtained and used in the next step without further purification. An analytically pure sample of **11** was obtained by a second column chromatography (50 % EtOAc in DCM).

**$^{1}$H NMR** (400 MHz, $CDCl_3$) $\delta$/ppm: 5.85–5.75 (m, 1H), 5.18–5.14 (m, 2H), 4.29 (brs, 1H), 3.98 (d, $J$ = 4.7 Hz, 1H), 3.98–3.93 (m, 1H), 3.75 (dd, $J_1$ = 9.7 Hz, $J_2$ = 4.7 Hz, 1H), 3.64–3.58 (m, 2H), 2.68 (brs, 1H), 2.54–2.43 (m, 2H), 1.83–1.75 (m, 1H), 1.66–1.45 (m, 5H), 1.43 (s, 3H), 1.32 (s, 3H), 1.26 (d, $J$ = 6.2 Hz, 3H), 0.89 (s, 9H), 0.04 (s, 6H)

**$^{13}$C NMR** (100 MHz, $CDCl_3$) $\delta$/ppm: 133.3, 119.6, 107.2, 82.0, 80.9, 74.7, 65.4, 63.1, 41.5, 35.0, 33.3, 28.3, 26.2, 26.0, 19.9, 19.2, 18.5

**HRMS** (ESI) $m/z$ $[M+Na]^+$ calc. ($C_{21}H_{42}O_5SiNa^+$) 425.2699, found 425.2689

$R_f$ = 0.48 (30 % EtOAc/Hex, KMnO4), $[\alpha]_D^{23}$ −24.4 ($c$ 1.00 $CHCl_3$)

**Preparation of triol 12**

((*S*)-5-((4*S*,5*S*)-5-((*S*)-1-hydroxyethyl)-2,2-dimethyl-1,3-dioxolan-4-yl)oct-7-ene-1,5-diol)

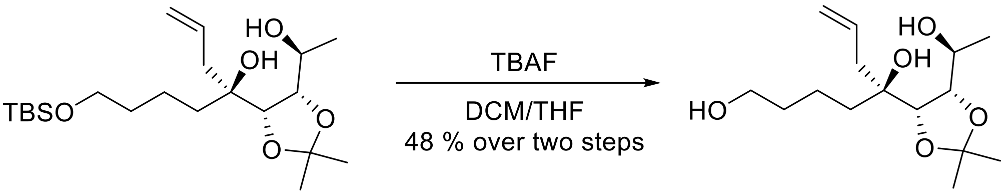


**11** **12**

A solution of TBAF ($c$ = 1.0 M in THF) (49 mL, 49 mmol) was added to a solution of **11** (6.59 g, 16.4 mmol) in dry DCM (49 mL) and the reaction mixture was stirred at RT overnight. The reaction mixture was concentrated *in vacuo* and purified by column chromatography (EtOAc). 3.70 g (48 % over two steps) of pure compound **12** were obtained as a colourless oil.

**$^{1}$H NMR** (400 MHz, $CDCl_3$) $\delta$/ppm: 5.85–5.75 (m, 1H), 5.19–5.13 (m, 2H), 4.56 (brs, 1H), 4.00–3.93 (m, 1H), 3.76 (dd, $J_1$ = 9.7 Hz, $J_2$ = 4.7 Hz, 1H), 3.65 (t, $J$ = 6.0 Hz, 2H), 3.28 (brs, 1H), 2.49 (d, $J$ = 7.5 Hz, 2H), 1.84–1.77 (m, 1H), 1.66–1.46 (m, 5H), 1.43 (s, 3H), 1.32 (s, 3H), 1.27 (d, $J$ = 6.4 Hz, 3H)

**$^{13}$C NMR** (100 MHz, $CDCl_3$) $\delta$/ppm: 133.4, 119.4, 107.2, 81.9, 80.9, 74.5, 65.4, 62.6, 41.4, 34.9, 32.9, 28.3, 25.9, 19.9, 19.0

**HRMS** (ESI) *m/z* $[M+Na]^+$ calc. ($C_{15}H_{28}O_5Na^+$) 311.1834, found 311.1826

$R_f$ = 0.38 (EtOAc, KMnO4), $[\alpha]_D^{23}$ −33.0 ($c$ 1.00 $CHCl_3$)

**Preparation of ketolactone 13**

((*S*)-6-((4*S*,5*R*)-5-acetyl-2,2-dimethyl-1,3-dioxolan-4-yl)-6-allyltetrahydro-2H-pyran-2-one)

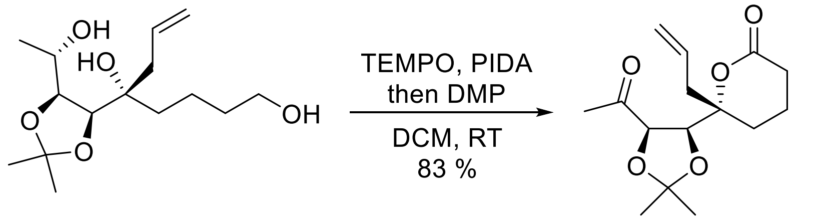


**12** **13**

To a solution of **12** (3.70 g, 12.8 mmol, 1 eq.) in dry DCM (123 mL) were added PIDA (14.8 g, 45.9 mmol, 3.6 eq.) and TEMPO (406 mg, 2.60 mmol, 0.2 eq.). After stirring the reaction mixture at RT for 3 h, DMP (7.6 g, 17.9 mmol, 1.4 eq.) was added and stirring was continued for 2.5 h. The reaction mixture was diluted with $Et_2O$ (70 mL), the resulting suspension was concentrated *in vacuo* and the crude residue was purified by short column

chromatography (60 % EtOAc in Hex). A second chromatographic purification (30 % EtOAc in Hex) afforded 3.00 g (83 %) of pure compound **13** as a white solid.

**$^{1}$H NMR** (400 MHz, $CDCl_3$) $\delta$/ppm: 5.76–5.66 (m, 1H), 5.23–5.17 (m, 2H), 4.41 (d, *J* = 7.9 Hz, 1H), 4.35 (d, *J* = 7.9 Hz, 1H), 2.88–2.22 (m, 1H), 2.53–2.31 (m, 3H), 2.27 (s, 3H), 2.10–2.00 (m, 1H), 1.95–1.74 (m, 3H), 1.68 (s, 3H), 1.38 (s, 3H)

**$^{13}$C NMR** (100 MHz, $CDCl_3$) $\delta$/ppm: 212.3, 169.8, 132.1, 120.6, 109.9, 84.1, 82.4, 80.9, 40.4, 29.6, 28.9, 26.7, 24.6, 23.8, 16.1

**HRMS** (ESI) *m*/*z* $[M+H]^+$ calc. ($C_{15}H_{23}O_5^+$) 283.1501, found 283.1538

$R_f$ = 0.50 (30 % EtOAc/Hex, KMnO4), $[\alpha]_D^{23}$ +23.4 (*c* 1.00 $CHCl_3$)

**Preparation of diene 15**

((*S*)-6-allyl-6-((4*S*,5*S*)-2,2-dimethyl-5-(prop-1-en-2-yl)-1,3-dioxolan-4-yl)tetrahydro-2H-pyran-2-one)

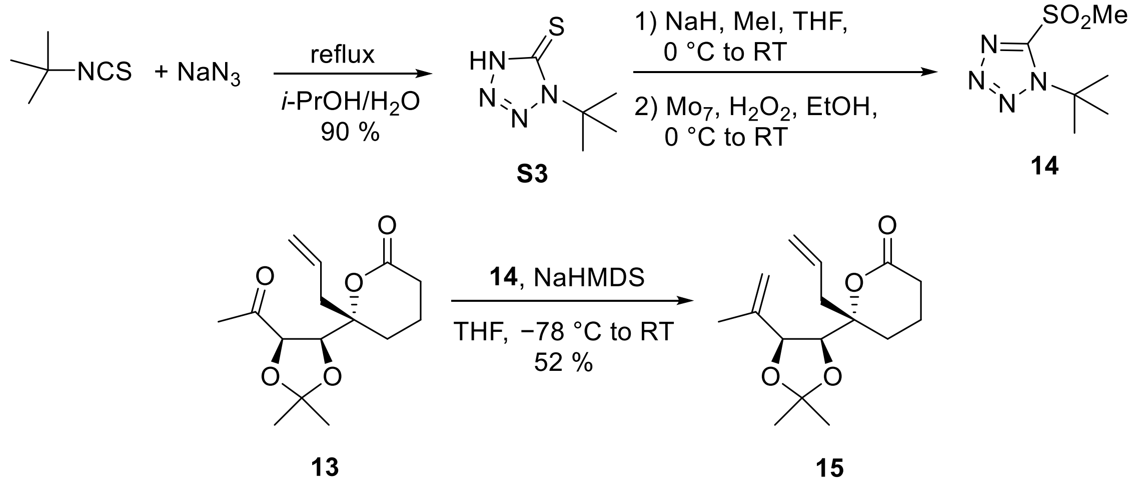


**Compound S3:** To a refluxing solution of $NaN_3$ (2.80 g, 43.1 mmol, 1 eq.) in water (13 mL) a solution of *t*-BuNCS (4.96 g, 43.1 mmol, 1 eq.) in *i*-PrOH (10 mL) was added dropwise over 30 min and refluxing was continued overnight. The reaction mixture was cooled to 0 °C, concentrated HCl (6.5 mL) was added carefully and the mixture was concentrated *in vacuo*. The contents were stored at 4 °C overnight during which the product crystallized. The solid was filtered off, washed with ice-cooled water and dried over $P_4O_{10}$ for 48 h. 5.02 g (74 %) of compound **S3** were obtained which were used in the next step without further purification.

**Compound 14**: A solution of **S3** (5.02 g, 31.7 mmol, 1 eq.) in dry THF (50 mL) was added dropwise to a suspension of NaH (60 wt % in mineral oil) (1.34 g, 33.5 mmol, 1.06 eq.) in dry THF (21 mL) at 0 °C. After stirring the mixture for 10 min, MeI (2.77 mL, 44.5 mmol, 1.4 eq.)

was added and the reaction mixture was stirred at RT overnight. The reaction was quenched by addition of a saturated solution of $NH_4Cl$ (50 mL), diluted with DCM, transferred to a separatory funnel and the phases were separated. The organic extract was washed with brine (50 mL), dried over anhydrous $Na_2SO_4$, filtered and concentrated *in vacuo*. 5.85 g of the crude solid were dissolved in EtOH (32 mL), the solution was cooled to 0 °C and a solution of $Mo_7O_{24}(NH_4)_6 \cdot 4H_2O$ (3.32 g, 2.69 mmol, 0.08 eq.) in $H_2O_2$ (32 mL) was added dropwise. The reaction mixture was stirred at RT for a few hours (monitored by TLC), then diluted with DCM (100 mL) and water (50 mL). The contents were transferred to a separatory funnel and the phases were separated. The organic extract was washed with brine (50 mL), dried over anhydrous $Na_2SO_4$, filtered and concentrated *in vacuo*. The crude solid was suspended, filtered, washed with pentane (3×15 mL) and dried in air. 5.84 g (90 %) of pure **14** were obtained as a white solid. $^1$H NMR spectrum is in agreement with previously reported spectra.[12]

**$^1$H NMR** (400 MHz, $CDCl_3$) $\delta$/ppm: 3.67 (s, 3H), 1.86 (s, 9H)

To a solution of **13** (3.00 g, 10.6 mmol, 1 eq.) and **14** (3.04 g, 14.9 mmol, 1.4 eq.) in dry THF (140 mL) at −78 °C was added NaHMDS ($c$ = 1.0 M in THF) (13.8 mL, 13.8 mmol, 1.3 eq.) and the reaction mixture was slowly warmed to RT and stirred overnight. The reaction was quenched by addition of a saturated solution of $NH_4Cl$ (80 mL), the contents were transferred to a separatory funnel and the phases were separated. The aqueous layer was extracted with $Et_2O$ (2×75 mL), the combined organic extracts were dried over anhydrous $Na_2SO_4$, filtered and concentrated *in vacuo*. 5.51 g of the crude residue were purified by column chromatography (0 % to 20 % EtOAc in DCM). 1.55 g (52 %) of pure compound **15** were obtained as a white solid.

**$^1$H NMR** (400 MHz, $CDCl_3$) $\delta$/ppm: 5.84–5.74 (m, 1H), 5.18–5.13 (m, 2H), 5.08 (m, 1H), 5.02 (m, 1H), 4.22 (d, $J$ = 6.9 Hz, 1H), 2.60 (dq, $J_1$ = 15.4 Hz, $J_2$ = 7.4 Hz, 2H), 2.50–2.43 (m, 1H), 2.40–2.33 (m, 1H), 1.96–1.73 (m, 7H), 1.59 (s, 3H), 1.37 (s, 3H)
**$^{13}$C NMR** (100 MHz, $CDCl_3$) $\delta$/ppm: 170.6, 143.2, 132.5, 120.0, 114.8, 108.1, 85.1, 80.7, 80.4, 40.6, 30.0, 26.6, 25.5, 25.0, 21.2, 20.9, 16.6, 14.4
**HRMS** (ESI) $m/z$ $[M+Na]^+$ calc. ($C_{16}H_{24}O_4Na^+$) 303.1572, found 303.1563
$R_f$ = 0.60 (15 % EtOAc/DCM, KMnO4), $[\alpha]_D^{23}$ +31.4 ($c$ 1.00 $CHCl_3$)

**Preparation of spirolactone 16**

((3a*S*,4*S*,7a*S*)-2,2,7-trimethyl-3a,4',5',7a-tetrahydro-5H-spiro[benzo[d][1,3]dioxole-4,2'-pyran]-6'(3'H)-one)

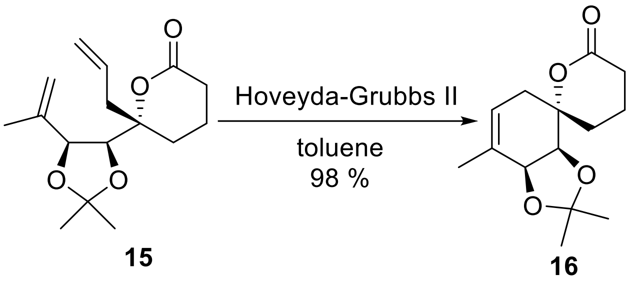


Grubbs-Hoveyda II catalyst (65 mg, 0.104 mmol, 2 mol %) was added to a solution of **15** (1.36 g, 4.85 mmol, 1 eq.) in toluene (125 mL). The reaction mixture was stirred at reduced pressure for 4 h, concentrated *in vacuo* and the residue was purified by column chromatography (60 % EtOAc in Hex). 1.20 g (98 %) of **16** was obtained as an off-white solid due to traces of ruthenium and was used in the next step without further purification.

**$^1$H NMR** (400 MHz, $CDCl_3$) $\delta$/ppm: 5.33 (m, 1H), 4.53 (d, $J$ = 5.1 Hz, 1H), 4.15 (dd, $J_1$ = 5.5 Hz, $J_2$ = 1.0 Hz, 1H), 2.61–2.46 (m, 2H), 2.41–2.35 (m, 1H), 2.27–2.21 (m, 1H), 2.09–2.02 (m, 1H), 2.00–1.79 (m, 3H), 1.78 (m, 3H), 1.39 (s, 3H), 1.36 (s, 3H)

**$^{13}$C NMR** (100 MHz, $CDCl_3$) $\delta$/ppm: 170.6, 133.0, 118.9, 109.9, 83.3, 76.7, 76.3, 33.6, 30.1, 29.9, 27.6, 27.0, 19.6, 16.1

**HRMS** (ESI) *m*/*z* $[M+Na]^+$ calc. ($C_{14}H_{20}O_4Na^+$) 275.1259, found 275.1248

$R_f$ = 0.58 (60 % EtOAc/Hex, KMnO4), $[\alpha]_D^{23}$ +73.0 (*c* 1.00 $CHCl_3$)

__**Preparation of diastereomers 17 and 18**__

((3a*S*,4*S*,5'*S*,7a*S*)- and (3a*S*,4*S*,5'*R*,7a*S*)-2,2,5',7-tetramethyl-3a,4',5',7a-tetrahydro-5H-spiro[benzo[d][1,3]dioxole-4,2'-pyran]-6'(3'H)-one)

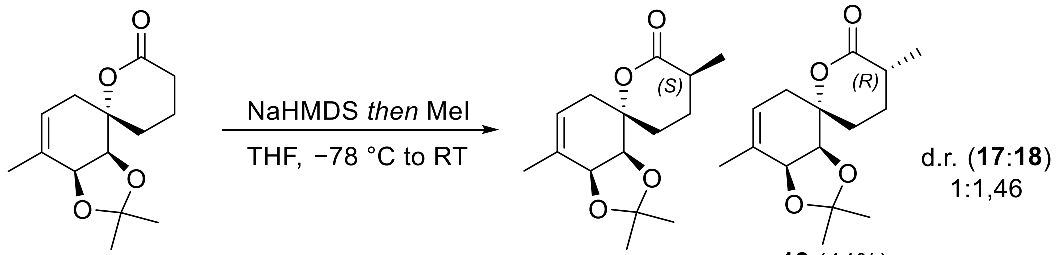


**16** **17** (30%) **18** (44%)

To a solution of **16** (1.00 g, 3.96 mmol, 1 eq.) in dry THF (32.5 mL) at −78 °C was added NaHMDS (*c* = 1.0 M in THF) (4.0 mL, 4.0 mmol, 1.01 eq.) dropwise over 5 min and stirring was continued for 50 min, after which MeI (500 µL, 8.03 mmol, 2 eq.) was added. The reaction mixture was warmed to −50 °C over 2 h and then to RT over 30 min. The mixture was concentrated *in vacuo* and 1.83 g of the residue was purified by column chromatography (10 % to 20 % EtOAc in Hex) to afford 315 mg (30 %) of pure **17** and 459 mg (44 %) of pure **18** as white solids.

**Compound 17:**

**$^1$H NMR** (400 MHz, $CDCl_3$) $\delta$/ppm: 5.34 (m, 1H), 4.54 (d, $J$ = 5.5 Hz, 1H), 4.11 (dd, $J_1$ = 5.7 Hz, $J_2$ = 0.6 Hz, 1H), 2.58–2.48 (m, 1H), 2.37–2.23 (m, 2H), 2.05–1.96 (m, 3H), 1.78 (m, 3H), 1.69–1.60 (m, 1H), 1.39 (s, 3H), 1.37 (s, 3H), 1.30 (d, $J$ = 7.2 Hz, 3H)

**$^{13}$C NMR** (100 MHz, $CDCl_3$) $\delta$/ppm: 174.2, 133.1, 119.1, 109.8, 83.5, 78.3, 76.4, 35.7, 33.4, 29.9, 27.5, 26.9, 24.8, 19.6, 17.8

**HRMS** (ESI) *m*/*z* $[M+H]^+$ calc. ($C_{15}H_{23}O_4^+$) 267.1591, found 267.1586

$R_f$ = 0.33 (30 % EtOAc/Hex, KMnO4), $[\alpha]_D^{23}$ +83.1 (*c* 0.77 $CHCl_3$)

**Compound 18:**

**$^1$H NMR** (400 MHz, $CDCl_3$) $\delta$/ppm: 5.32 (m, 1H), 4.52 (d, $J$ = 4.9 Hz, 1H), 4.15 (dd, $J_1$ = 5.4 Hz, $J_2$ = 1.0 Hz, 1H), 2.50–2.36 (m, 2H), 2.26–2.17 (m, 2H), 1.97–1.88 (m, 1H), 1.82–1.68 (m, 5H), 1.39 (s, 3H), 1.35 (s, 3H), 1.31 (d, $J$ = 7.1 Hz, 3H)

**$^{13}$C NMR** (100 MHz, $CDCl_3$) $\delta$/ppm: 174.1, 132.8, 119.0, 109.9, 83.4, 76.3, 75.7, 35.7, 34.5, 30.2, 27.7, 27.1, 24.7, 19.6, 17.6

**HRMS** (ESI) *m*/*z* $[M+H]^+$ calc. ($C_{15}H_{23}O_4^+$) 267.1591, found 267.1588

$R_f$ = 0.39 (30 % EtOAc/Hex, KMnO4)

**Preparation of compound 19**

((3*S*,6*S*,10*S*,11*S*)-3,9-dimethyl-10,11-bis((trimethylsilyl)oxy)-1-oxaspiro[5.5]undec-8-en-2-one)

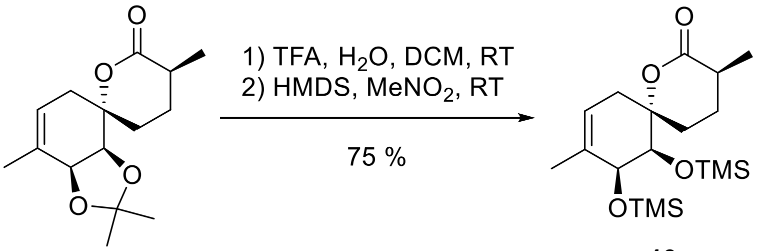


**17** **19**

To a solution of **17** (207 mg, 0.777 mmol, 1 eq.) in DCM (4 mL) were added water (100 µL) and TFA (4.0 mL) and the reaction mixture was stirred at RT for 1.5 h. The mixture was concentrated *in vacuo*, the crude residue was dissolved in $MeNO_2$ (4 mL) and HMDS (410 µL, 1.96 mmol, 2.5 eq.) was added. The reaction mixture was stirred at RT for 5 min, concentrated *in vacuo* and purified by column chromatography (0 % to 20 % EtOAc in Hex). 216 mg (75 %) of pure compound **19** was obtained as a white solid.

**$^1$H NMR** (400 MHz, $CDCl_3$) $\delta$/ppm: 5.24 (m, 1H), 4.05 (d, $J$ = 3.4 Hz, 1H), 3.79 (d, $J$ = 4.0 Hz, 1H), 2.47–2.41 (m, 1H), 2.40–2.30 (m, 1H), 2.25–2.21 (m, 1H), 2.10–2.04 (m, 1H), 1.93–1.86 (m, 2H), 1.70 (m, 3H), 1.57–1.46 (m, 1H), 1.26 (d, $J$ = 7.0 Hz, 3H), 0.15 (s, 9H), 0.15 (s, 9H)

**$^{13}$C NMR** (100 MHz, $CDCl_3$) $\delta$/ppm: 174.9, 135.0, 119.6, 85.8, 75.6, 73.0, 37.0, 36.3, 27.2, 25.5, 20.6, 17.5, 0.9, 0.8

**HRMS** (ESI) *m/z* $[M+Na]^+$ calc. ($C_{18}H_{34}O_4Si_2Na^+$) 393.1893, found 393.1884

$R_f$ = 0.60 (30 % EtOAc/Hex, KMnO4), $[\alpha]_D^{23}$ +124.9 (*c* 0.85 $CHCl_3$)

**Preparation of compound 20**

((3*S*,6*S*,7*S*,8*R*,9*R*)-9-hydroxy-3,9-dimethyl-7,8-bis((trimethylsilyl)oxy)-1-oxaspiro[5.5]undecan-2-one)

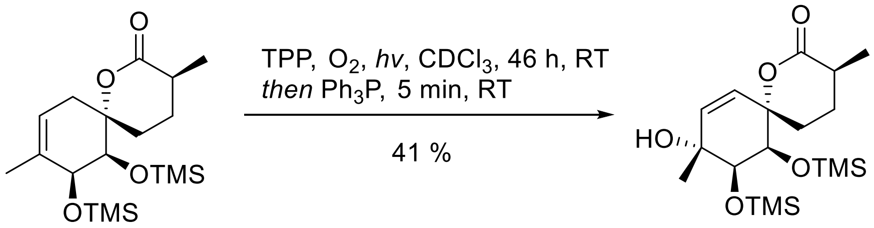


**19** **20**

A solution of **19** (194 mg, 0.513 mmol, 1 eq.) in $CDCl_3$ (20 mL) under an atmosphere of $O_2$ was charged with TPP (8 mg, 0.013 mmol, 2.5 mol %) and irradiated with a 250 W tungsten

halogen lamp projector at RT for 46 h. $PPh_3$ was added, the mixture was stirred for 5 min, filtered over a cotton plug and concentrated *in vacuo*. The crude residue was purified by flash column chromatography (20 % EtOAc in Hex). 80.8 mg (41 %) of pure compound **20** was obtained as a colourless oil.

**$^1$H NMR** (400 MHz, $CDCl_3$) $\delta$/ppm: 5.76 (brs, 1H), 5.58 (brs, 1H), 4.23 (brs, 1H), 3.75 (brs, 1H), 2.40–2.26 (m, 2H), 1.97–1.87 (m, 2H), 1.72–1.59 (m, 2H), 1.29–1.25 (m, 6H), 0.16 (s, 9H), 0.14 (s, 9H)

**HRMS** (ESI) *m/z* $[M+Na]^+$ calc. ($C_{18}H_{34}O_5Si_2Na^+$) 409.1842, found 409.1832

$R_f$ = 0.26 (25 % EtOAc/Hex, KMnO4), $[\alpha]_D^{23}$ +86.5 (*c* 0.18 $CHCl_3$)

**<u>Preparation of enone 21</u>**

((3*S*,6*R*,10*S*,11*S*)-3,9-dimethyl-10,11-bis((trimethylsilyl)oxy)-1-oxaspiro[5.5]undec-8-ene-2,7-dione)

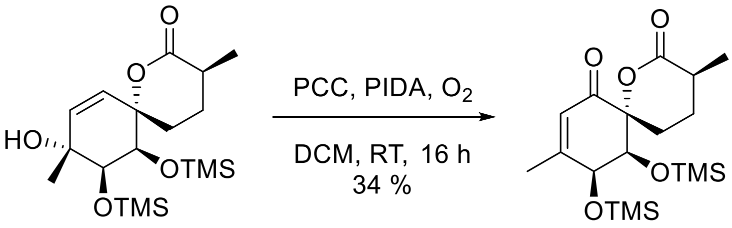


**20** **21**

A solution of **20** (80.8 mg, 0.209 mmol, 1 eq.) and PIDA (200 mg, 0.621 mmol, 3 eq.) in dry DCM (1.62 mL) was charged with PCC (5.4 mg, 0.025 mmol, 12 mol %) and the reaction mixture was stirred under an atmosphere of $O_2$ overnight at room temperature. The mixture was directly absorbed onto silica and purified by flash column chromatography (10 % to 40 % EtOAc in Hex). 27.2 mg (34 %) of pure compound **21** was obtained as a white solid and 31.0 mg (38 %) of the starting material **20** was recovered. Recrystallization of **21** from $Et_2O$ afforded high quality single crystals which were used to determine its crystal structure by SCXRD. General and crystallographic data are presented in Table S1.

**$^1$H NMR** (400 MHz, $CDCl_3$) $\delta$/ppm: 5.87 (m, 1H), 4.18 (d, $J$ = 3.5 Hz, 1H), 4.02 (d, $J$ = 4.1 Hz, 1H), 2.41–2.31 (m, 1H), 2.25–2.11 (m, 2H), 2.02 (d, $J$ = 1.3 Hz, 3H), 1.81–1.74 (m, 1H), 1.58–1.44 (m, 1H), 1.30 (d, $J$ = 6.9 Hz, 3H), 0.19 (s, 9H), 0.18 (s, 9H)

**$^{13}$C NMR** (100 MHz, $CDCl_3$) $\delta$/ppm: 195.8, 174.3, 159.5, 124.8, 73.7, 73.0, 35.9, 25.19, 25.17 22.1, 17.0, 0.85, 0.79

**HRMS** (ESI) *m/z* $[M+H]^+$ calc. ($C_{18}H_{33}O_5Si_2^+$) 385.1867, found 385.1857

$R_f$ = 0.48 (25 % EtOAc/Hex, KMnO4), $[\alpha]_D^{23}$ +193.6 (*c* 0.37 $CHCl_3$)

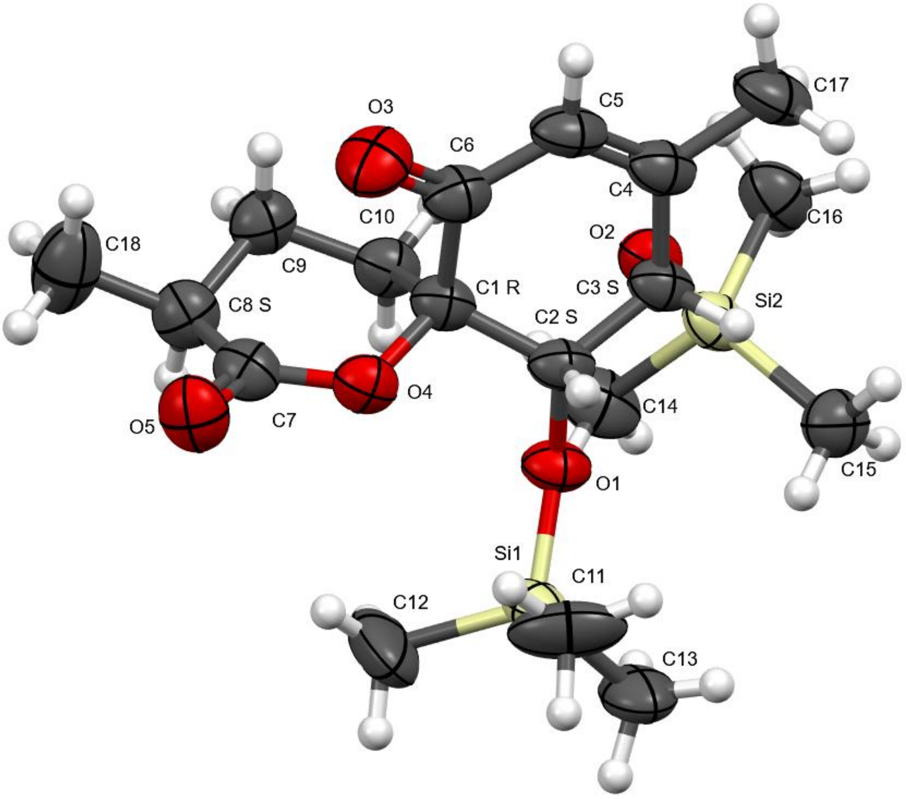


**Figure S2.** Asymmetric unit of compound **21**. Non-hydrogen atoms are shown as thermal ellipsoids at 30% probability level. Atoms C1, C2, C3 and C8 are stereocenters, and their stereochemistry is included in the label.

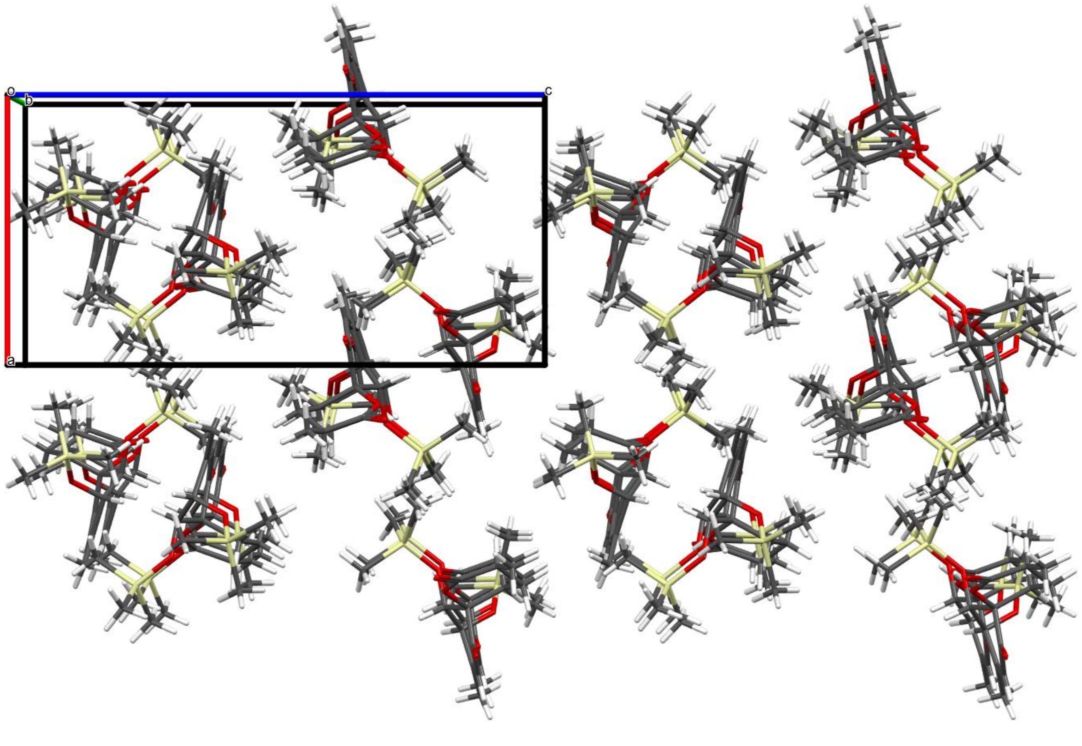

**Figure S3.** Packing of molecules in the crystal structure of **21** shown along crystallographic axis *b*.

**Table S1.** General and crystallographic data for compound **21**

| Identifier | **Compound 21** |
|---|---|
| Empirical formula | $C_{18}H_{32}O_5Si_2$ |
| $M_r$ | 384.61 |
| $T$ / K | 298.4(4) |
| Crystal system | orthorhombic, colourless needle |
| Space group | $P2_12_12_1$ |
| $a$ / Å | 9.5802(8) |
| $b$ / Å | 12.4686(11) |
| $c$ / Å | 19.0884(18) |
| $\alpha$ / ° | 90 |
| $\beta$ / ° | 90 |
| $\gamma$ / ° | 90 |
| $V$ / Å$^3$ | 2280.1(4) |
| $Z$ | 4 |
| $\rho_{calc}$ / g cm$^{-3}$ | 1.120 |
| $\mu$ / mm$^{-1}$ | 1.596 |
| $F$(000) | 832 |
| Crystal size / mm$^3$ | 0.202 × 0.043 × 0.039 |
| Radiation | CuKα ($\lambda$ = 1.54184 Å) |
| 2$\Theta$ range / ° | 8.47 to 135.972 |
| Index ranges | $-11 \leq h \leq 11$<br>$-10 \leq k \leq 14$<br>$-22 \leq l \leq 22$ |
| Reflections collected | 14354 |
| Independent reflections | 4137 ($R_{int}$ = 8.61%, $R_{sigma}$ = 9.34%) |
| Data/restraints/parameters | 4137/0/234 |
| $g_1$, $g_2$ in $w^a$ | 0.1410, 0 |
| Goodness-of-fit on $F^2$, $S^b$ | 0.989 |
| Final $R$ and $wR^c$ values [$I \geq 2\sigma(I)$] | $R_1$ = 7.33%, $wR_2$ = 19.0% |
| Final $R$ and $wR^c$ values [all data] | $R_1$ = 9.48%, $wR_2$ = 21.27% |
| Largest diff. peak/hole / e Å$^{-3}$ | 0.482/−0.337 |
| Flack parameter, $x^d$ | 0.03(4) |

[a] $w = 1/[\sigma F_o^2 + (g_1P)^2 + g_2P]$ where $P = (F_o^2 + 2F_c^2)/3$

[b] $S = \{\Sigma[w(F_o^2 - F_c^2)^2]/(N_r - N_p)\}^{1/2}$ where $N_r$ = number of independent reflections, $N_p$ = number of refined parameters.

[c] $R = \Sigma||F_o| - |F_c||/\Sigma|F_o|$; $wR = \{\Sigma[w(F_o^2 - F_c^2)^2]/\Sigma[w(F_o2)2]\}^{1/2}$

[d] $I(hkl) = (1 - x)|F(hkl)|^2 + x|F(-h-k-l)|^2$, where $x$ is the refined Flack parameter.

**Table S2.** Selected bond lengths in the crystal structure of compound **21**

| Atoms | Bond length/Å | Atoms | Bond length/Å | Atoms | Bond length/Å |
|---|---|---|---|---|---|
| C1–C2 | 1.511(9) | O2–C3 | 1.419(7) | Si1–C13 | 1.829(9) |
| C1–C6 | 1.531(9) | O3–C6 | 1.206(10) | Si1–O1 | 1.644(4) |
| C1–C10 | 1.525(8) | O4–C1 | 1.455(7) | Si2–C14 | 1.872(8) |
| C2–C3 | 1.519(9) | O4–C7 | 1.347(8) | Si2–C15 | 1.866(8) |
| C3–C4 | 1.520(8) | O5–C7 | 1.185(9) | Si2–C16 | 1.864(8) |
| C4–C5 | 1.313(12) | Si1–C11 | 1.857(11) | Si2–O2 | 1.645(4) |
| O1–C2 | 1.418(7) | Si1–C12 | 1.836(12) | | |

**Preparation of (+)-1**

((3*S*,6*R*,10*S*,11*S*)-10,11-dihydroxy-3,9-dimethyl-1-oxaspiro[5.5]undec-8-ene-2,7-dione)

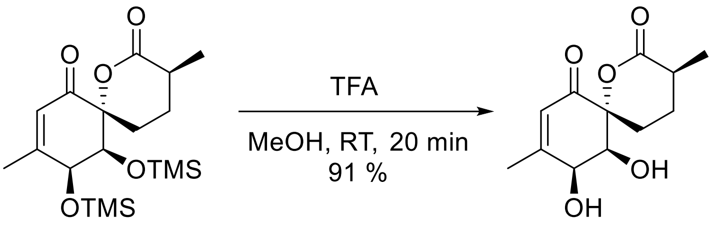


**21** (+)-**1**

To a solution of **21** (13.7 mg, 0.0356 mmol, 1 eq.) in MeOH (1 mL) was added TFA (10 µL, 0.13 mmol, 3.7 eq.) and the reaction mixture was stirred at room temperature for 20 min, concentrated *in vacuo* and purified by flash column chromatography (EtOAc). 7.77 mg (91 %) of pure (+)-**1** was obtained as a white solid.

**$^1$H NMR** (600 MHz, DMSO-d$_6$) $\delta$/ppm: 5.88 (m, 1H), 5.69 (d, $J$ = 5.7 Hz, 1H), 5.62 (d, $J$ = 6.4 Hz, 1H), 4.16 (t, $J$ = 5.0 Hz, 1H), 3.83 (dd, $J_1$ = 6.3 Hz, $J_2$ = 4.6 Hz, 1H), 2.44 – 2.37 (m, 1H), 2.22 (td, $J_1$ = 14.8 Hz, $J_2$ = 4.0 Hz, 1H), 2.02 (d, $J$ = 1.2 Hz, 3H), 2.00 (dt, $J_1$ = 15.2 Hz, $J_2$ = 3.6 Hz, 1H), 1.79 – 1.74 (m, 1H), 1.27 – 1.20 (m, 1H), 1.12 (d, $J$ = 6.9 Hz, 3H)

**$^{13}$C NMR** (151 MHz, DMSO-d$_6$) $\delta$/ppm: 196.1, 173.8, 162.0, 123.4, 70.8, 69.3, 34.7, 24.6, 21.6, 16.9

**HRMS** (ESI) *m/z* [M+Na]$^+$ calc. ($C_{12}H_{16}O_5Na^+$) 263.0895, found 263.0886

$R_f$ = 0.33 (EtOAc, KMnO4), $[\alpha]_D^{23}$ +201.2 ($c$ 0.24 $CHCl_3$)

### 1.4. Preparation of synthetic derivatives 22 – 24

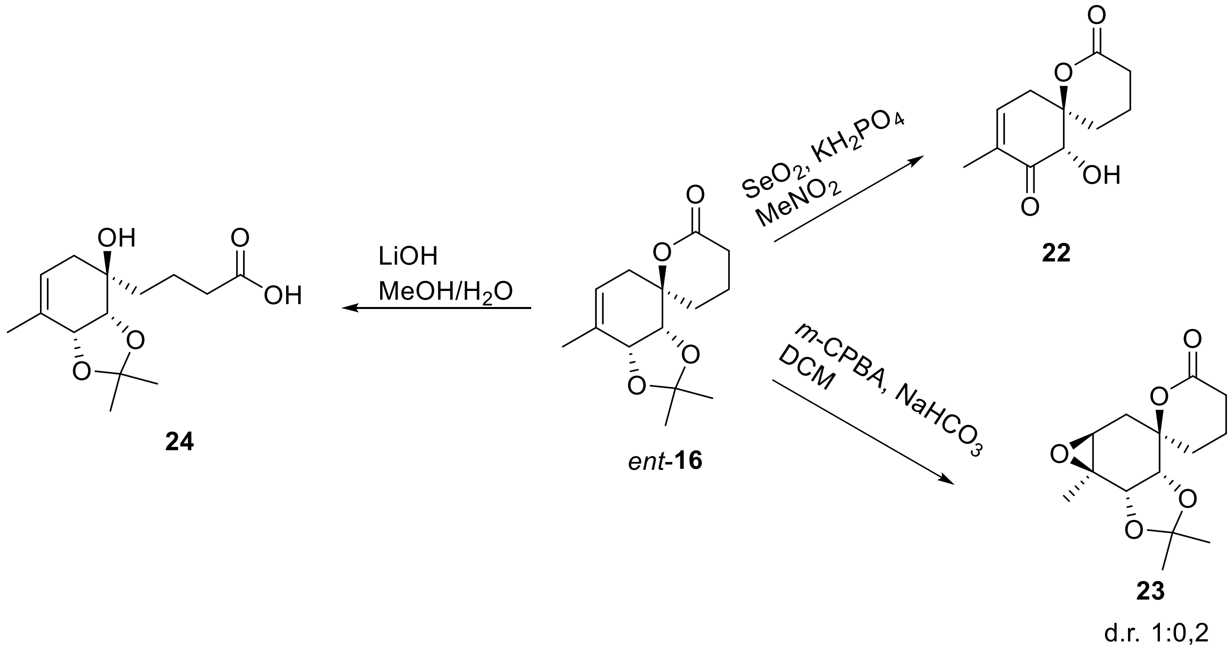


**Compound 22** ((6*S*,11*S*)-11-hydroxy-9-methyl-1-oxaspiro[5.5]undec-8-ene-2,7,10-trione)

To a solution of spirolactone *ent*-**16** (20 mg, 0.079 mmol, 1 eq.) in $MeNO_2$ (600 μL) were added $KH_2PO_4$ (34 mg, 0.250 mmol, 3.2 eq.), then $SeO_2$ (19.4 mg, 0.175 mmol, 2.2 eq.) and the reaction mixture was heated at 60 °C for 2 h. The reaction mixture was cooled to room temperature, filtered through a 0.20 μm syringe filter and concentrated *in vacuo*. The crude mixture was purified by column chromatography (20 % to 50 % EtOAc in DCM) to afford 5.4 mg (30 %) of pure **22** as a white solid.

**$^1$H NMR** (600 MHz, $CDCl_3$) $\delta$/ppm: 6.66–6.64 (m, 1H), 4,46 (d, $J$ = 2.4 Hz, 1H), 3,78 (d, $J$ = 2.4 Hz, 1H), 2.87–2.78 (m, 2H), 2.59–2.55 (m, 1H), 2.48–2.42 (m, 1H), 1.98–1.88 (m, 2H), 1,88–1.87 (m, 3H), 1.77–1.70 (m, 1H), 1.60–1.56 (m, 1H)

**$^{13}$C NMR** (151 MHz, $CDCl_3$) $\delta$/ppm: 197.8, 170.8, 141.9, 134.2, 86.5, 79.6, 37.7, 30.3, 23.1, 17.0, 15.3

**HRMS** (ESI) *m*/*z* $[M+Na]^+$ calc. ($C_{11}H_{14}O_4Na^+$) 233.0790, found 233.0781

**Compound 23** ((3a*R*,4*R*,6b*S*)-2,2,6a-trimethylhexahydro-5H-spiro[oxireno[2',3':3,4]benzo[1,2-d][1,3]dioxole-4,2'-pyran]-6'(3'H)-one)

To a solution of spirolactone *ent*-**16** (20 mg, 0.079 mmol, 1 eq.) in DCM (500 μL) were added $NaHCO_3$ (12 mg, 0.14 mmol, 1.8 eq.), then *m*-CPBA (21.3 mg, 0.123 mmol, 1.2 eq.) and the

reaction mixture was stirred at room temperature for 24 h. The reaction was quenched by addition of a saturated solution of $NaHCO_3$ (1 mL) and DCM (1 mL), stirred for 10 minutes, transferred to a separatory funnel and the phases were separated. The aqueous layer was extracted with DCM (3×3 mL), dried over anhydrous $Na_2SO_4$, filtered and concentrated *in vacuo*. The crude mixture was purified by column chromatography (20 % to 50 % EtOAc in DCM) to yield 6.3 mg (30 %) of **23** as an inseparable mixture of diastereomers (d.r. 1:0.2).

**$^1$H NMR** (600 MHz, $CDCl_3$) $\delta$/ppm:
**MAJOR:** 4.47 (d, $J$ = 5.8 Hz, 1H), 4.10–4.09 (m, 1H), 3.09 (m, 1H), 2.59–2.46 (m, 2H), 2.29–2.12 (m, 2H), 2.01–1.84 (m, 3H), 1.80–1.74 (m, 1H), 1.46 (s, 3H), 1.41 (s, 3H), 1.38 (s, 3H)
**MINOR:** 4.45 (d, $J$ = 6.4 Hz, 1H), 4.10–4.09 (m, 1H), 3.14 (d, $J$ = 4.9 Hz, 1H), 2.59–2.46 (m, 2H), 2.29–2.12 (m, 2H), 2.08–2.03 (m, 1H), 2.01–1.84 (m, 3H), 1.54 (s, 3H), 1.46 (s, 3H), 1.37 (s, 3H)
**$^{13}$C NMR** (151 MHz, $CDCl_3$) $\delta$/ppm:
**MAJOR:** 169.8, 109.6, 80.4, 76.8, 74.7, 58.4, 56.3, 32.5, 30.6, 29.5, 27.5, 26.0, 19.4, 16.0
**MINOR:** 76.5, 76.0, 58.7, 33.8, 30,3 29.9, 26.4, 26.2, 21.7, 16.3
**HRMS** (ESI) *m/z* $[M+Na]^+$ calc. ($C_{14}H_{20}O_5Na^+$) 291.1208, found 291.1998

<u>**Compound 24**</u>

(4-((3a*R*,4*R*,7a*R*)-4-hydroxy-2,2,7-trimethyl-3a,4,5,7a-tetrahydrobenzo[d][1,3]dioxol-4-yl)butanoic acid)

To a solution of spirolactone *ent*-**16** (300 mg, 1.19 mmol, 1 eq.) in MeOH (5 mL) was added solution of $LiOH \times H_2O$ (225 mg, 5.36 mmol, 4.5 eq.) in water (500 µL). The reaction mixture was stirred at room temperature for 16 h, concentrated *in vacuo* and diluted with water (1 mL). HCl ($c$ = 1 M, 5 mL) was added dropwise at 0 °C, the mixture was transferred to a separatory funnel, extracted with $Et_2O$ (3×10 mL), dried over anhydrous $Na_2SO_4$, filtered and concentrated *in vacuo* to afford 321 mg (>99 %) of pure **25** as a viscous colourless oil.

**$^1$H NMR** (400 MHz, $CDCl_3$) $\delta$/ppm: 5.41–5.40 (m, 1H), 4.46–4.45 (m, 1H), 4.05 (dd, $J_1$ = 5.4 Hz, $J_2$ = 1.2 Hz, 1H), 2.44 (td, $J_1$ = 7.6 Hz, $J_2$ = 2.0 Hz, 2H), 2.36–2.30 (m, 1H), 2.08–2.02 (m, 1H), 1.90–1.62 (m, 5H), 1.81 (s, 3H), 1.40 (s, 3H), 1.35 (s, 3H)
**$^{13}$C NMR** (100 MHz, $CDCl_3$) $\delta$/ppm: 179.3, 133.8, 120.1, 109.9, 78.5, 76.3, 72.9, 38.3, 34.3, 33.1, 27.9, 27.0, 19.8, 17.8
**HRMS** (ESI) *m/z* $[M+Na]^+$ calc. ($C_{14}H_{22}O_5Na^+$) 293.1359, found 293.1382

# References


[1] Rigaku Oxford Diffraction. CrysAlisPro Software System, Version 1.171.42.49; Rigaku Oxford Diffraction: Oxford, UK, 2020.

[2] G. M. Sheldrick, SHELXT– Integrated space-group and crystal-structure determination. *Acta Crystallogr. A Found. Adv.* 2015, **71**, 3–8.

[3] G. M. Sheldrick, Crystal structure refinement with SHELXL. *Acta Crystallogr. C Struct. Chem.* 2015, **71**, 3-8. doi:10.1107/s2053229614024218.

[4] O. V. Dolomanov, L. J. Bourhis, R. J. Gildea, J. A. K. Howard, Puschmann, H. OLEX$^2$: a complete structure solution, refinement and analysis program. *J. Appl. Crystallogr.* 2009, **42**, 339–341.

[5] A. L. Spek, Structure validation in chemical crystallography. *Acta Crystallogr. D Biol. Crystallogr.* 2009, **65**, 148–155.

[6] C. R. Groom, I. J. Bruno, M. P. Lightfoot, S. C. Ward, The Cambridge Structural Database. *Acta Crystallogr. B Struct. Sci. Cryst. Eng. Mater.* 2016, **72**, 171–179.

[7] M. Nakata, M. Arai, K. Tomooka, N. Ohsawa, M. Kinoshita, Total synthesis of Erythronolide A. *Bull. Chem. Soc. Jpn.* 1989, **62**, 2618–2635.

[8] A. V. Subrahmanyam, K. Palanichamy, K. P. Kaliappan, Application of an Enyne Metathesis/Diels–Alder Cycloaddition Sequence: A New Versatile Approach to the Syntheses of *C*-Aryl Glycosides and Spiro-*C*-Aryl Glycosides. *Chem. Eur. J.* 2010, **16**, 8545–8556.

[9] W.-S. Song, S.-X. Liu. C.-C. Chang, Synthesis of L-Deoxyribonucleosides from D-Ribose. *J. Org. Chem.* 2018, **83**, 14923–14932.

[10] J. Zeng, S. Vedachalam, S. Xiang, X.-W. Liu, Direct *C*-Glycosylation of Organotrifluoroborates with Glycosyl Fluorides and Its Application to the Total Synthesis of (+)-Varitriol. *Org. Lett.* 2011, **13**, 42–45.

[11] S. Jana, V. A. Sarpe, S. S. Kulkarni, Total Synthesis of Emmyguyacins A and B, Potential Fusion Inhibitors of Influenza Virus. *Org. Lett.* 2018, **20**, 6938–6942.

[12] C. Aïssa, Improved Julia−Kocienski Conditions for the Methylenation of Aldehydes and Ketones. *J. Org. Chem.* 2006, **71**, 360–363.

## 2. NMR spectra

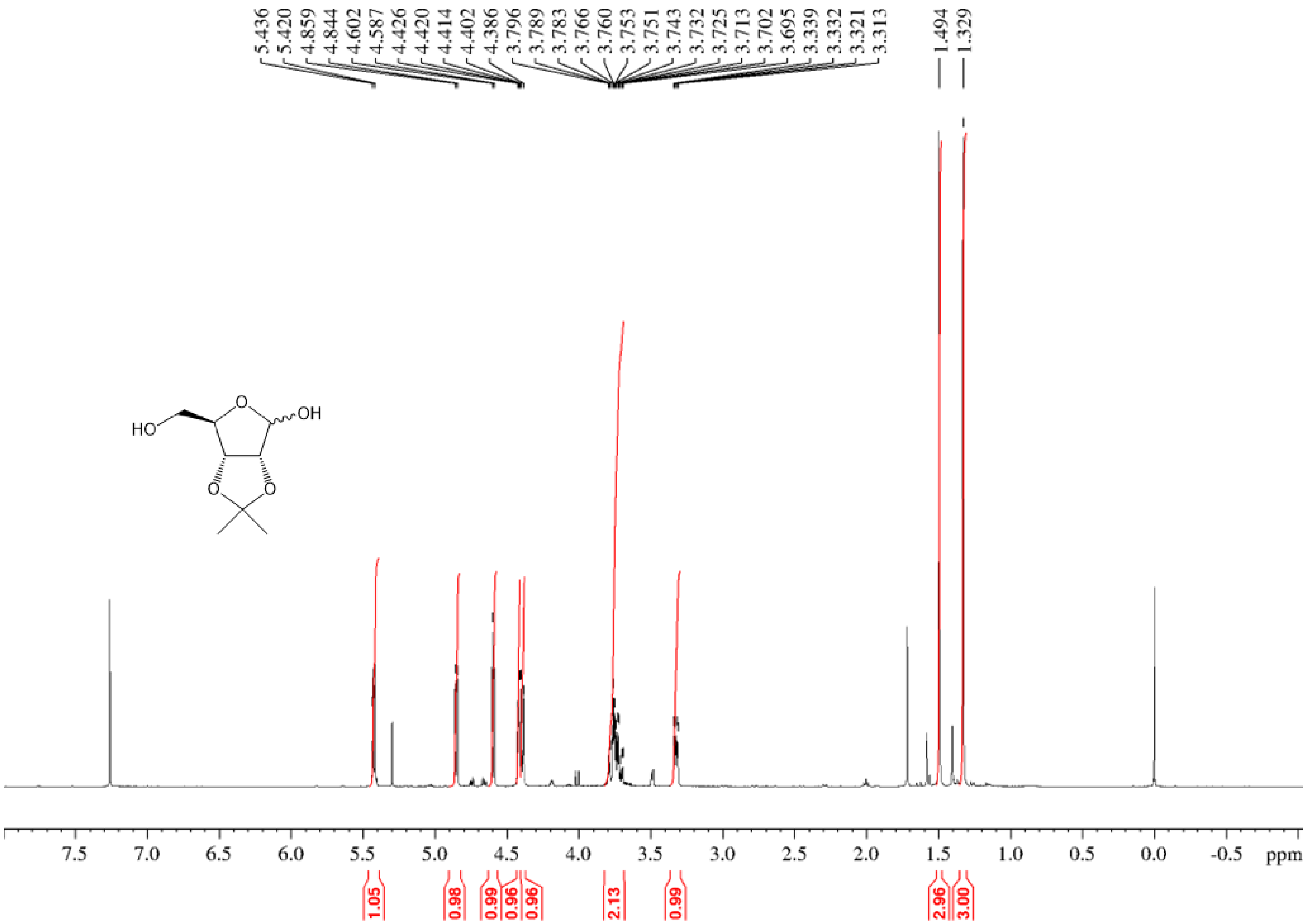


**Figure S4.** $^{1}H$ NMR spectrum of compound **6** in $CDCl_3$.

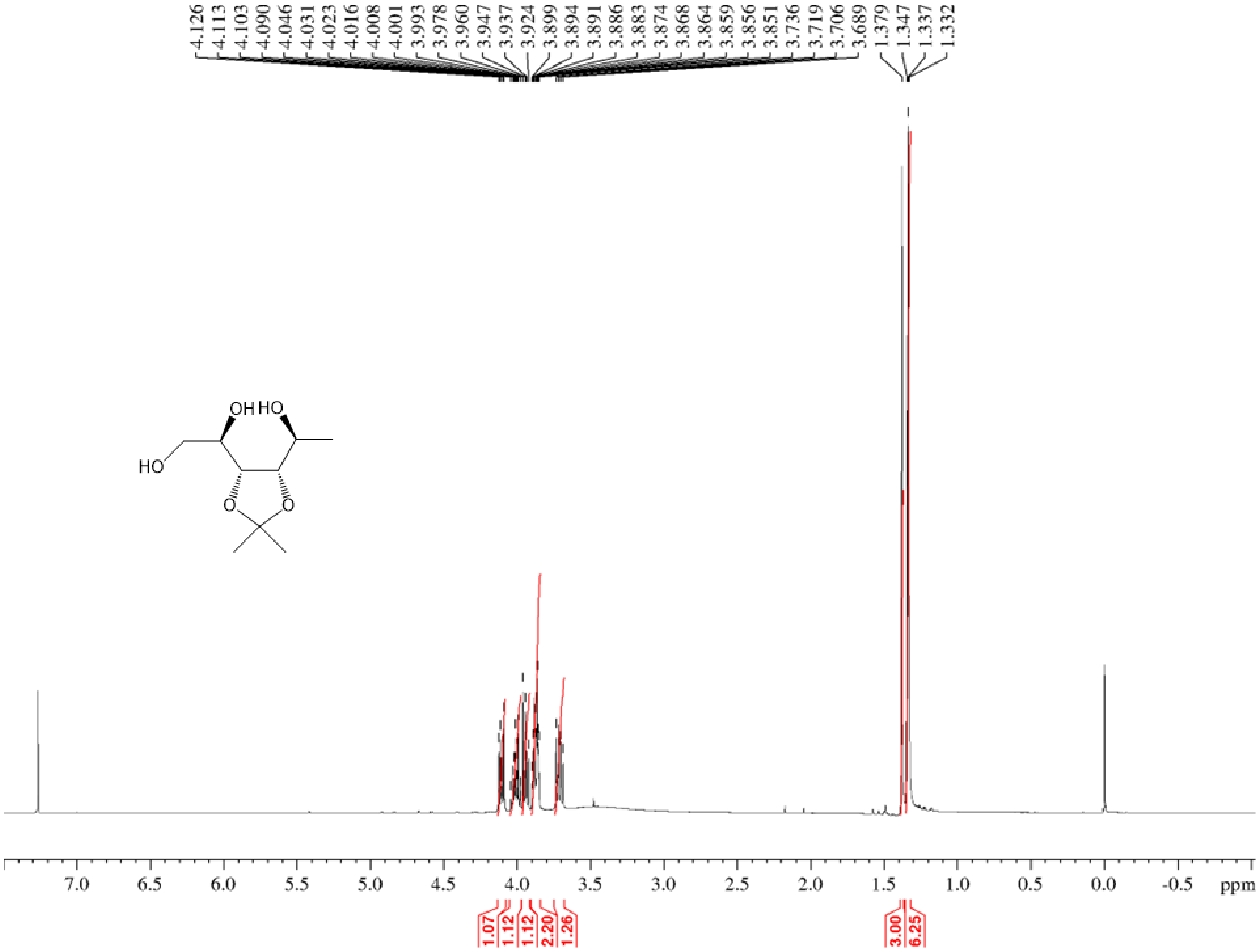

**Figure S5.** $^1$H NMR spectrum of compound **7** in $CDCl_3$.

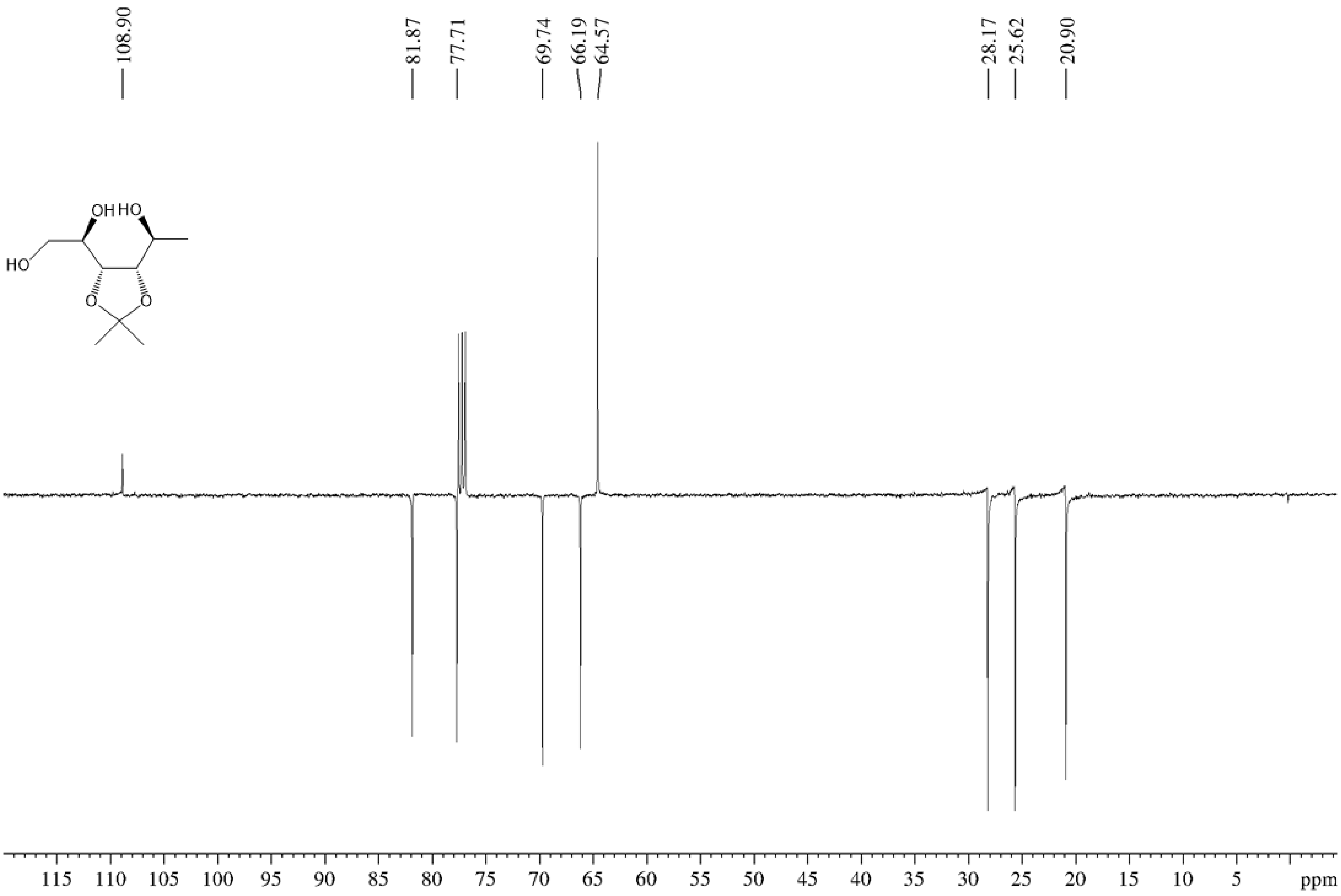


**Figure S6.** $^{13}$C NMR spectrum of compound **7** in $CDCl_3$.

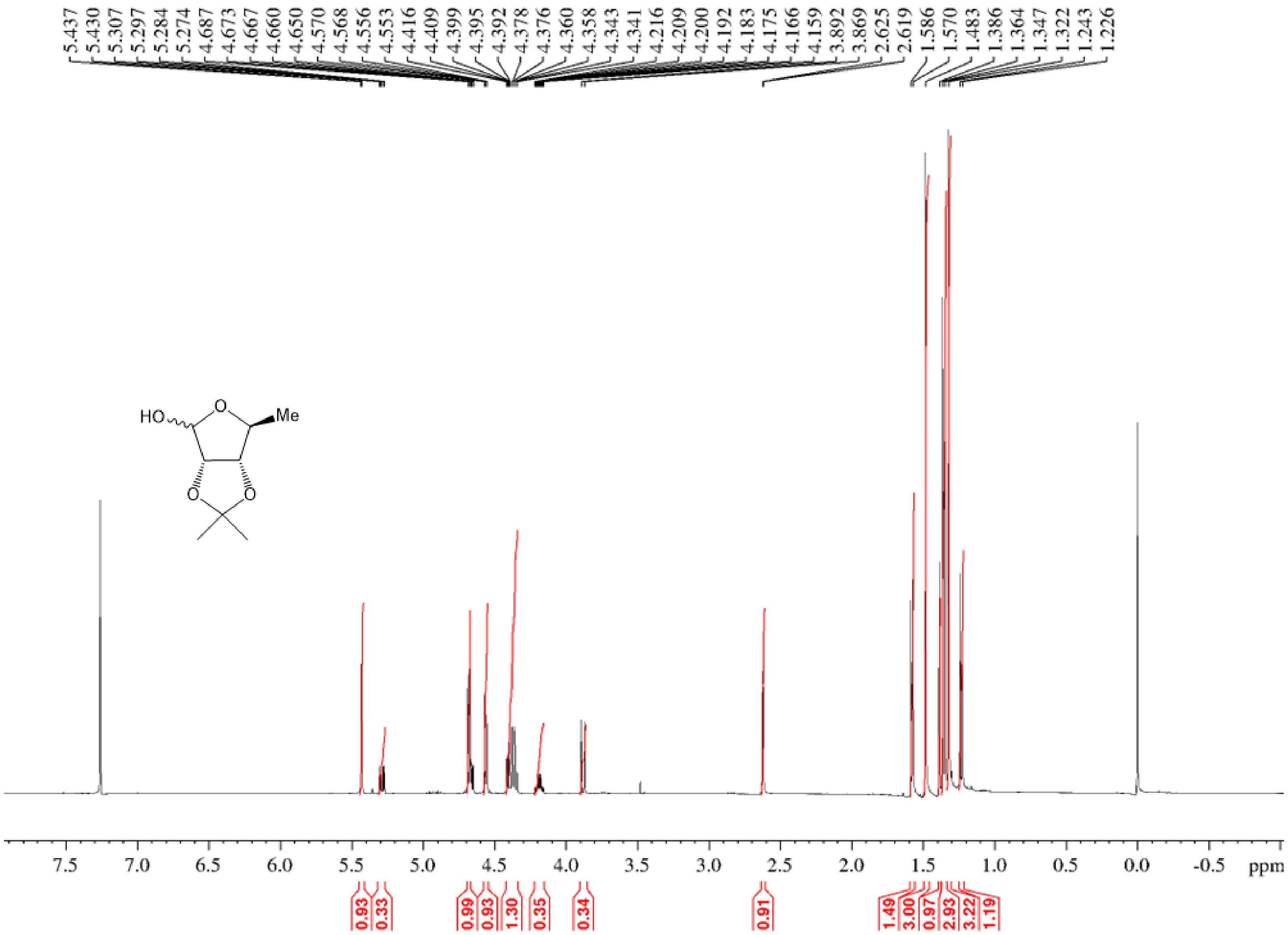

**Figure S7.** $^{1}$H NMR spectrum of compound **8** in $CDCl_3$.

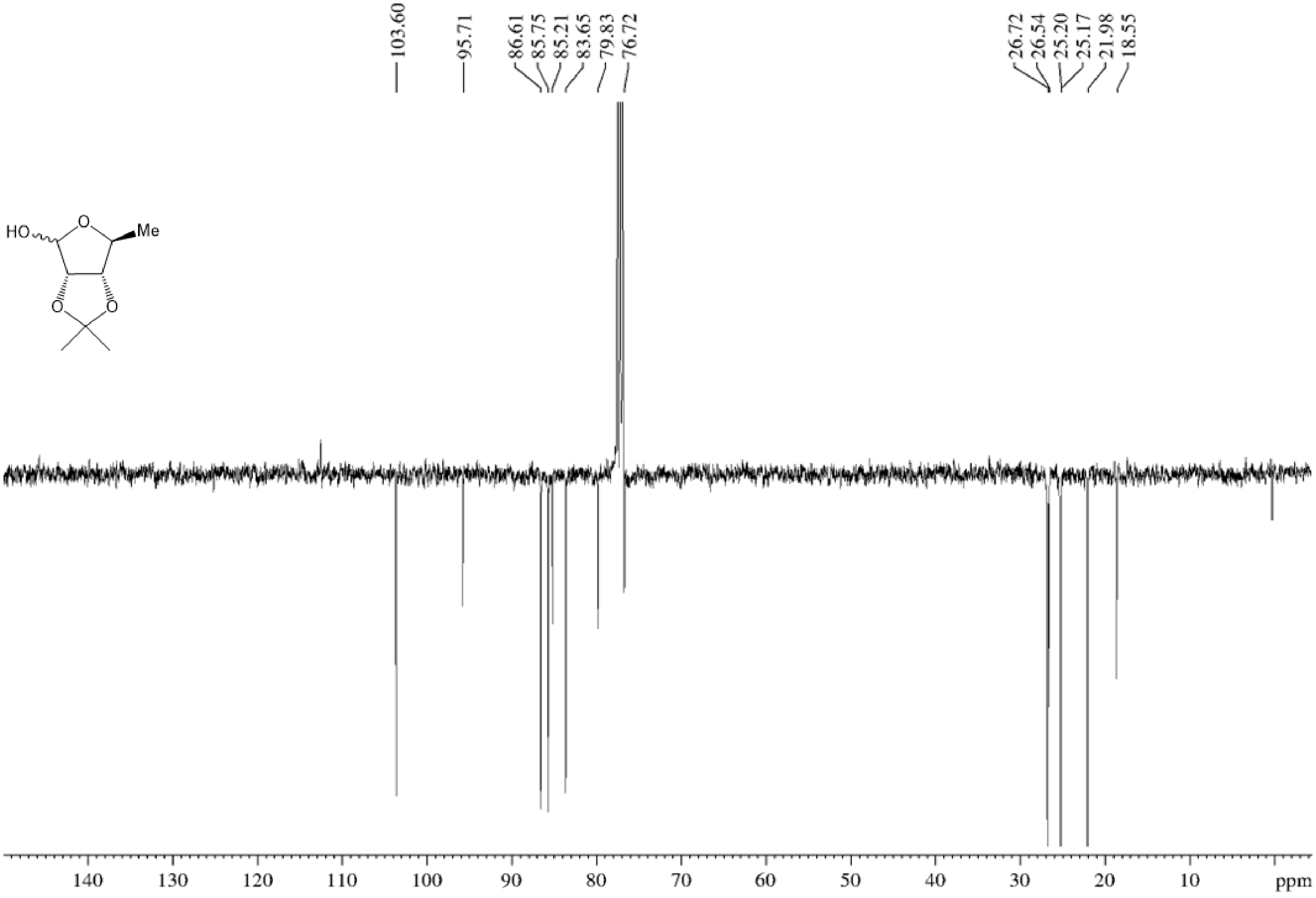


**Figure S8.** $^{13}C$ NMR spectrum of compound **8** in $CDCl_3$.

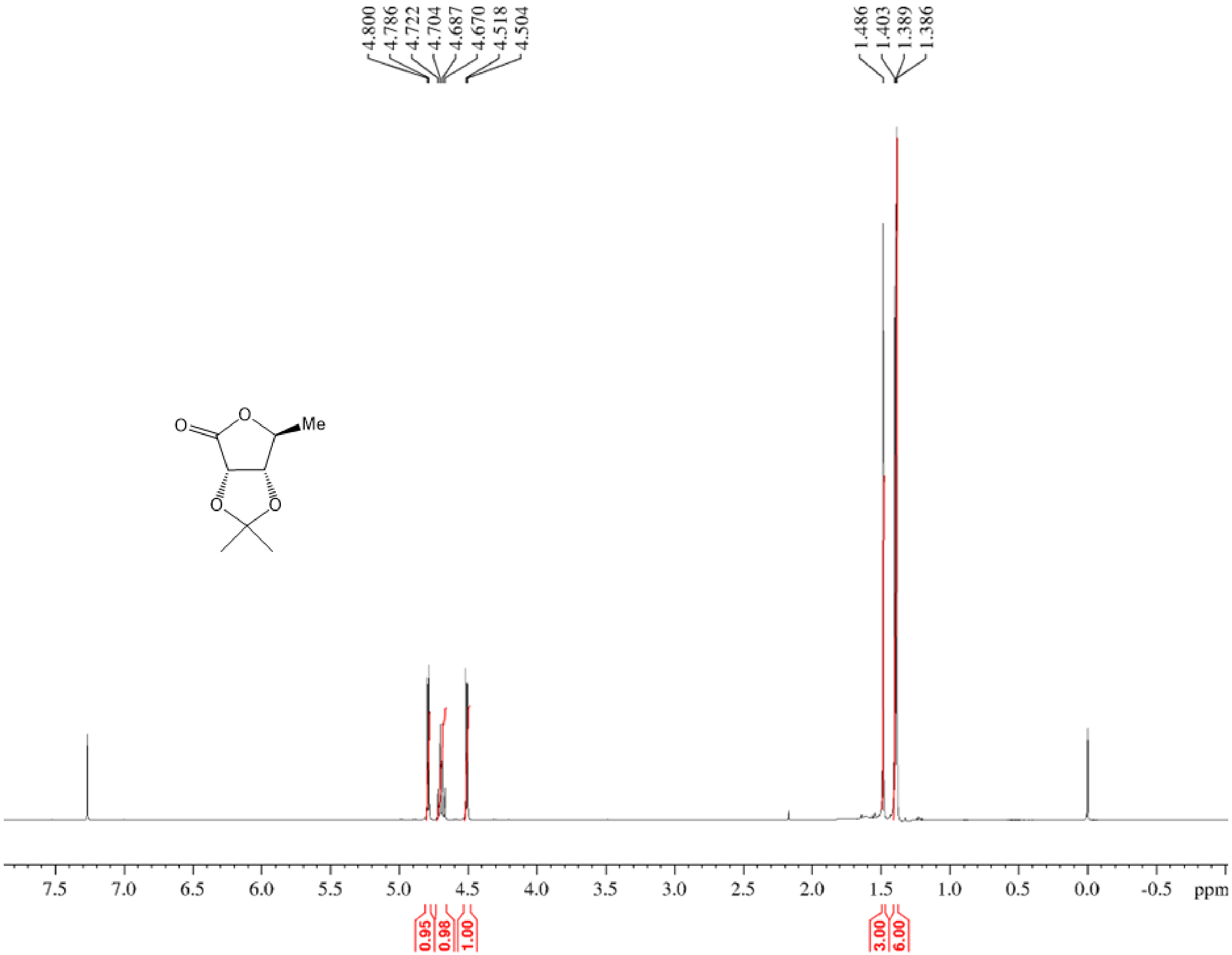


**Figure S9.** $^{1}$H NMR spectrum of compound **9** in $CDCl_3$.

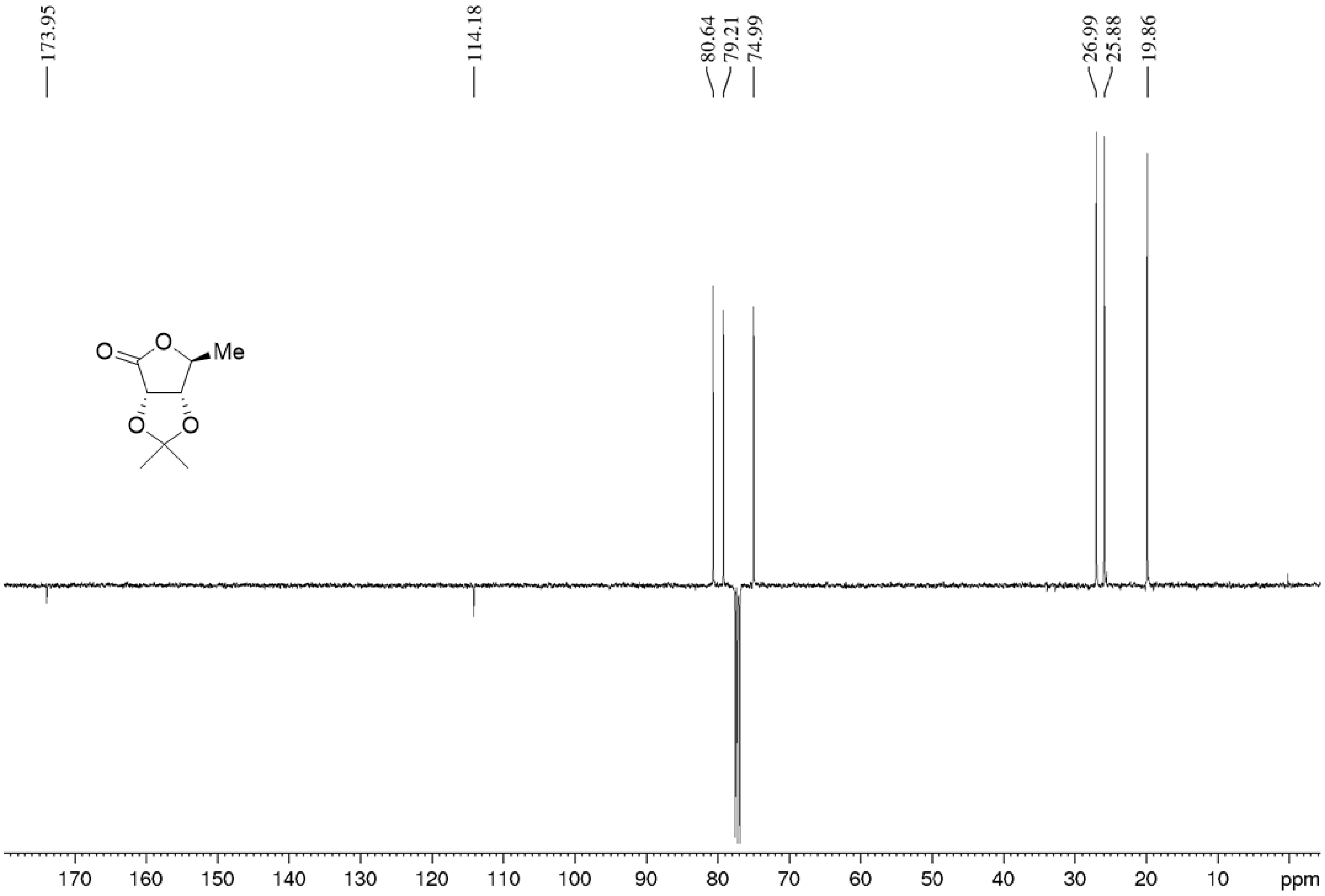


**Figure S10.** $^{13}$C NMR spectrum of compound **9** in $CDCl_3$.

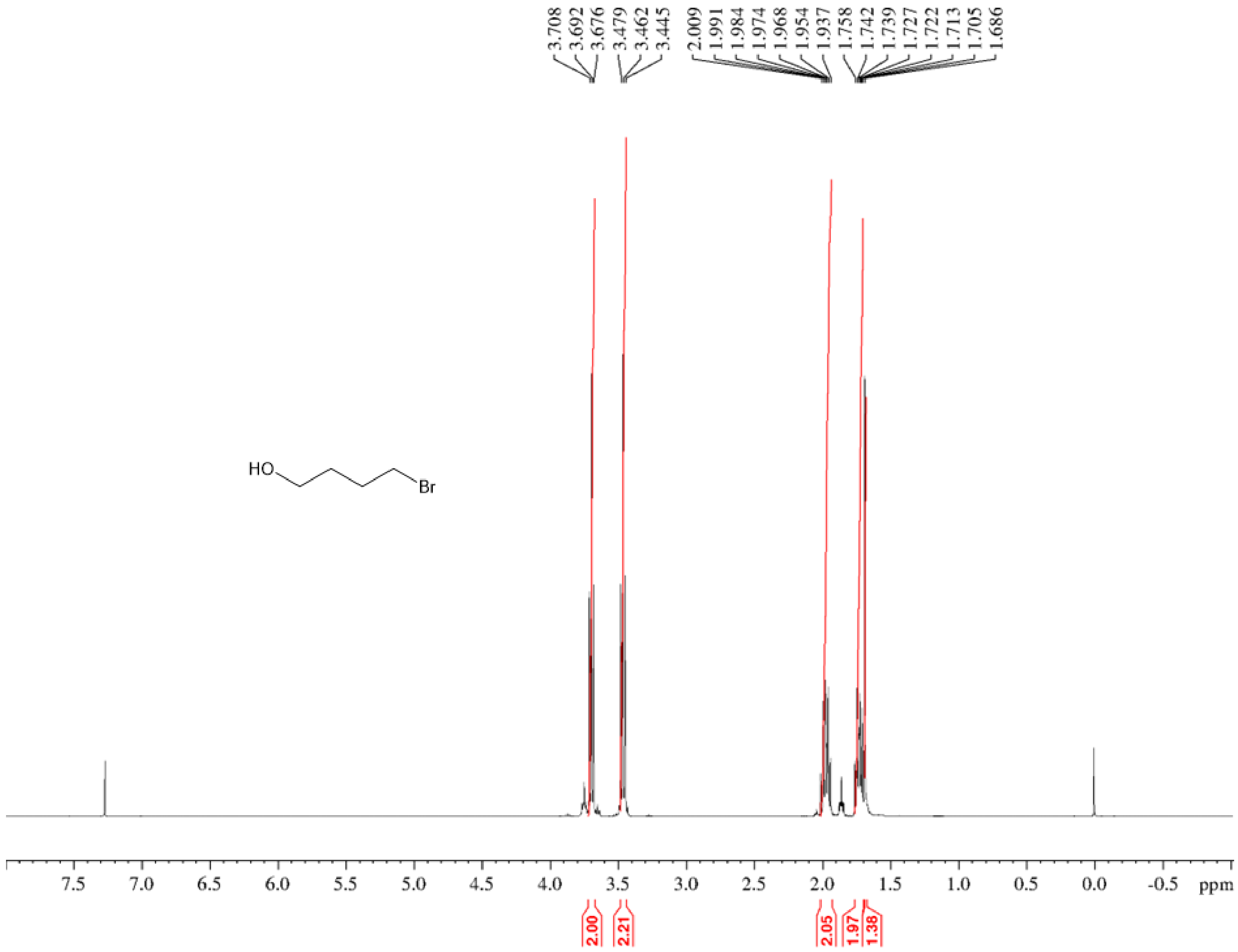


**Figure S11.** $^1$H NMR spectrum of compound **S1** in $CDCl_3$.

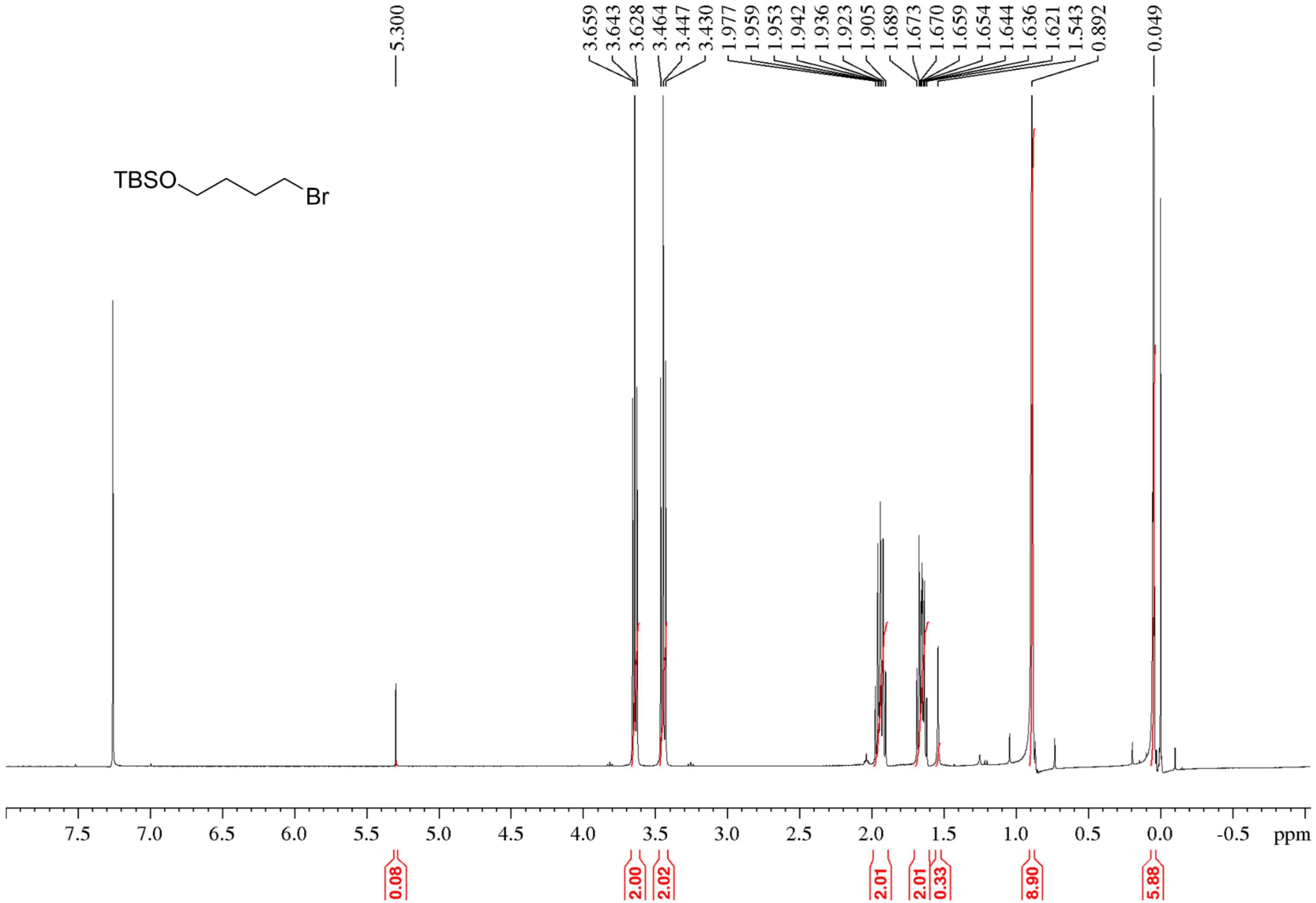


**Figure S12.** $^{1}H$ NMR spectrum of compound **S2** in $CDCl_3$.

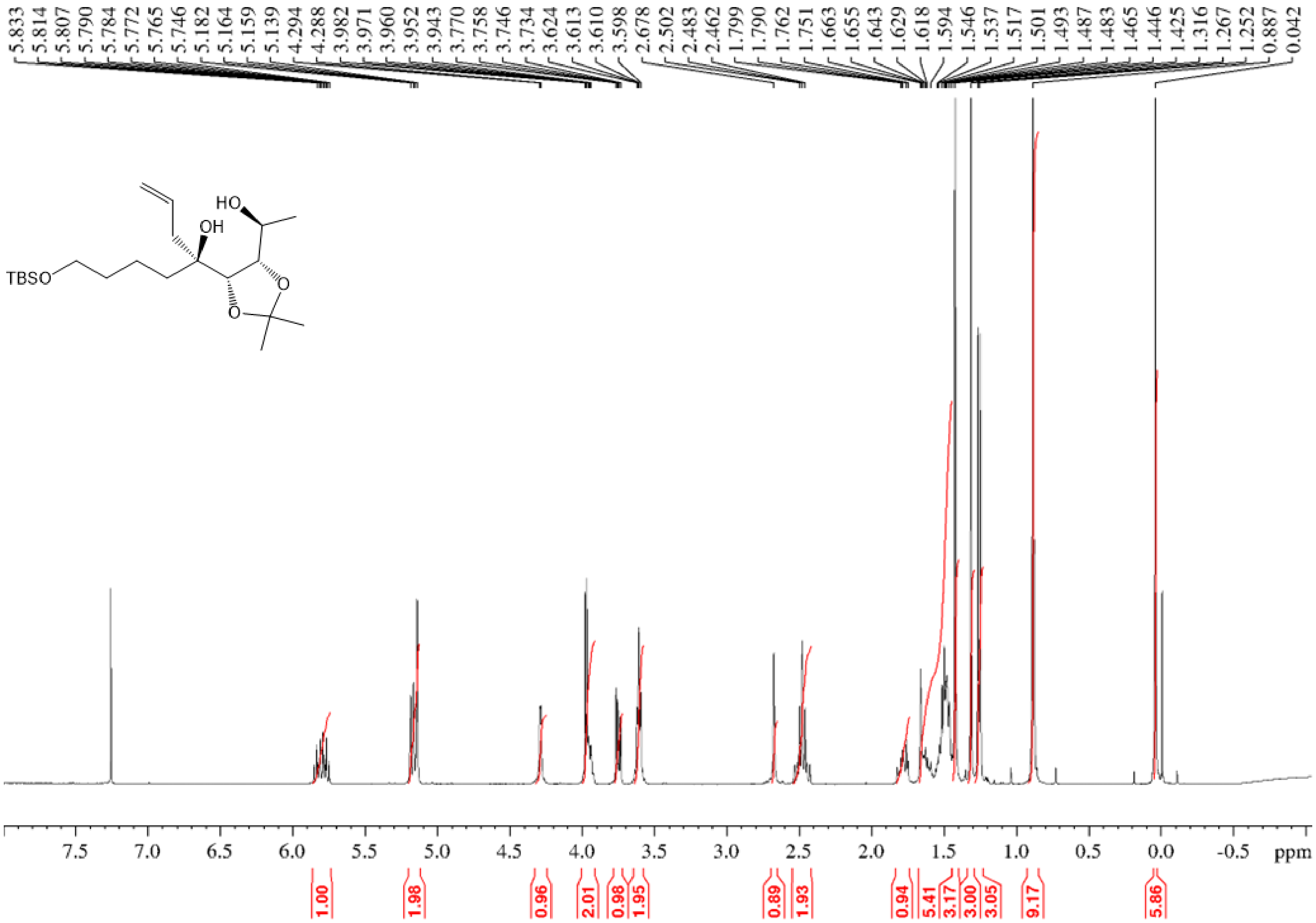

**Figure S13.** $^{1}$H NMR spectrum of compound **11** in $CDCl_3$.

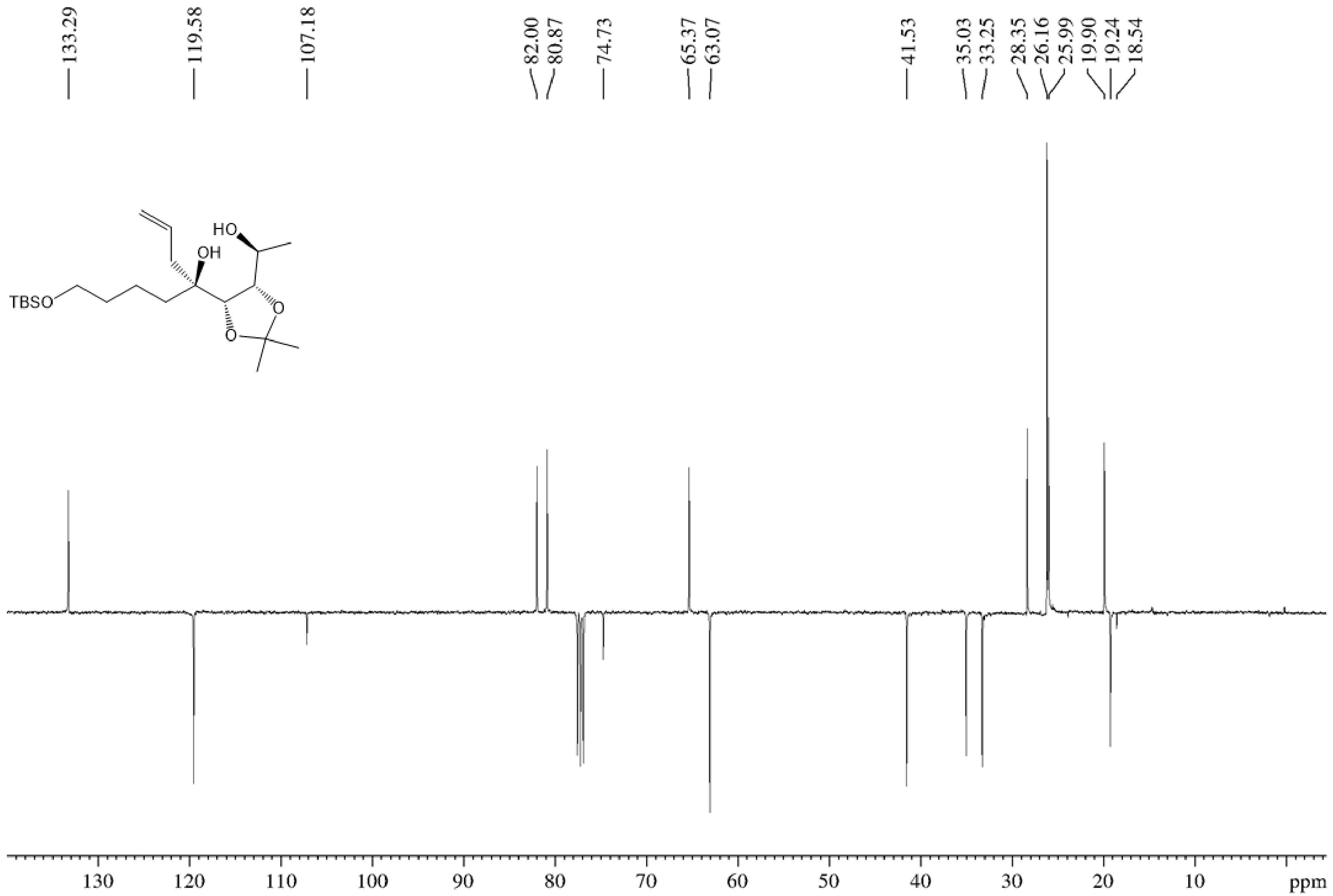


**Figure S14.** $^{13}$C NMR spectrum of compound **11** in $CDCl_3$.

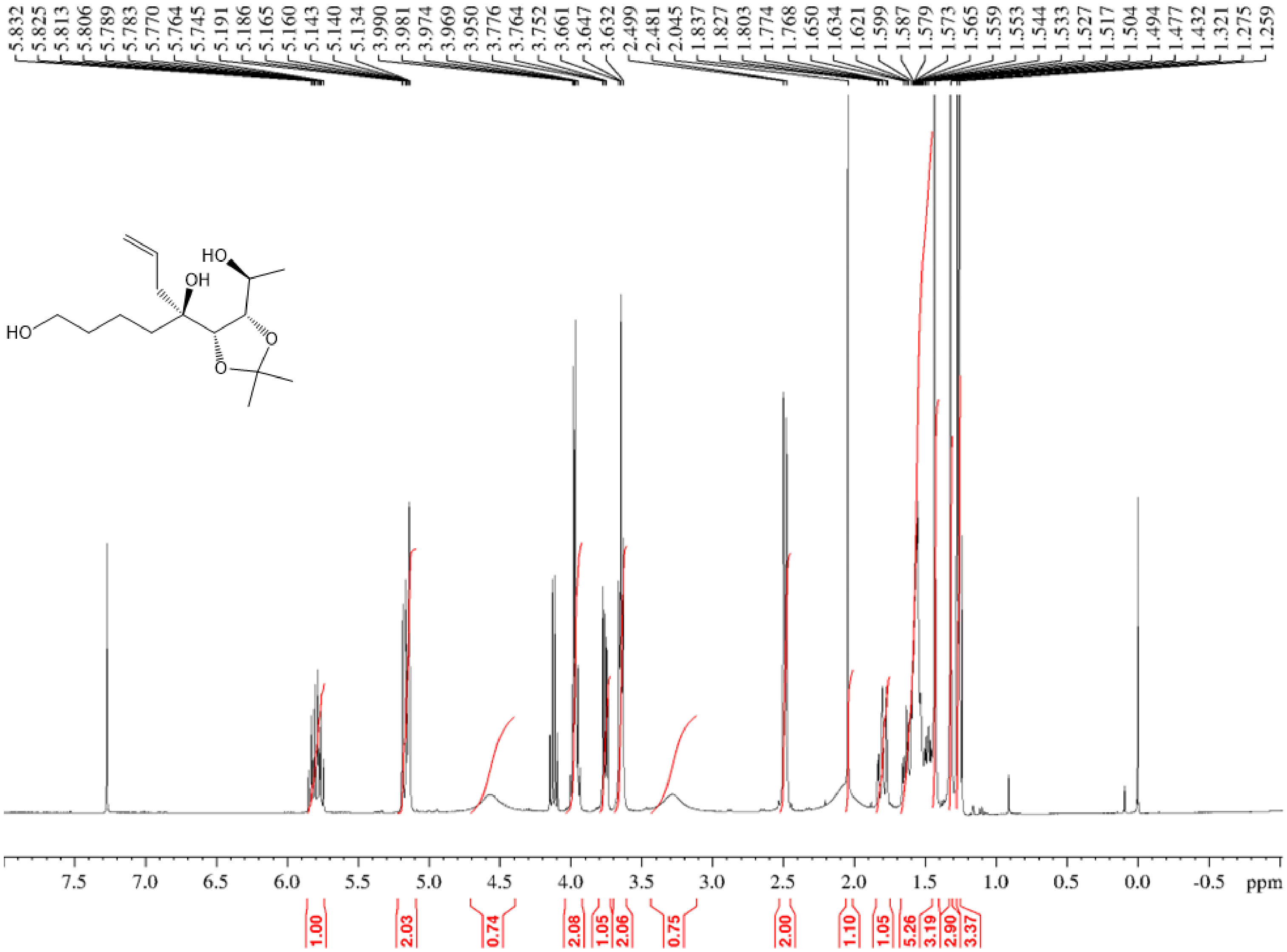


**Figure S15.** $^{1}$H NMR spectrum of compound **12** in $CDCl_3$.

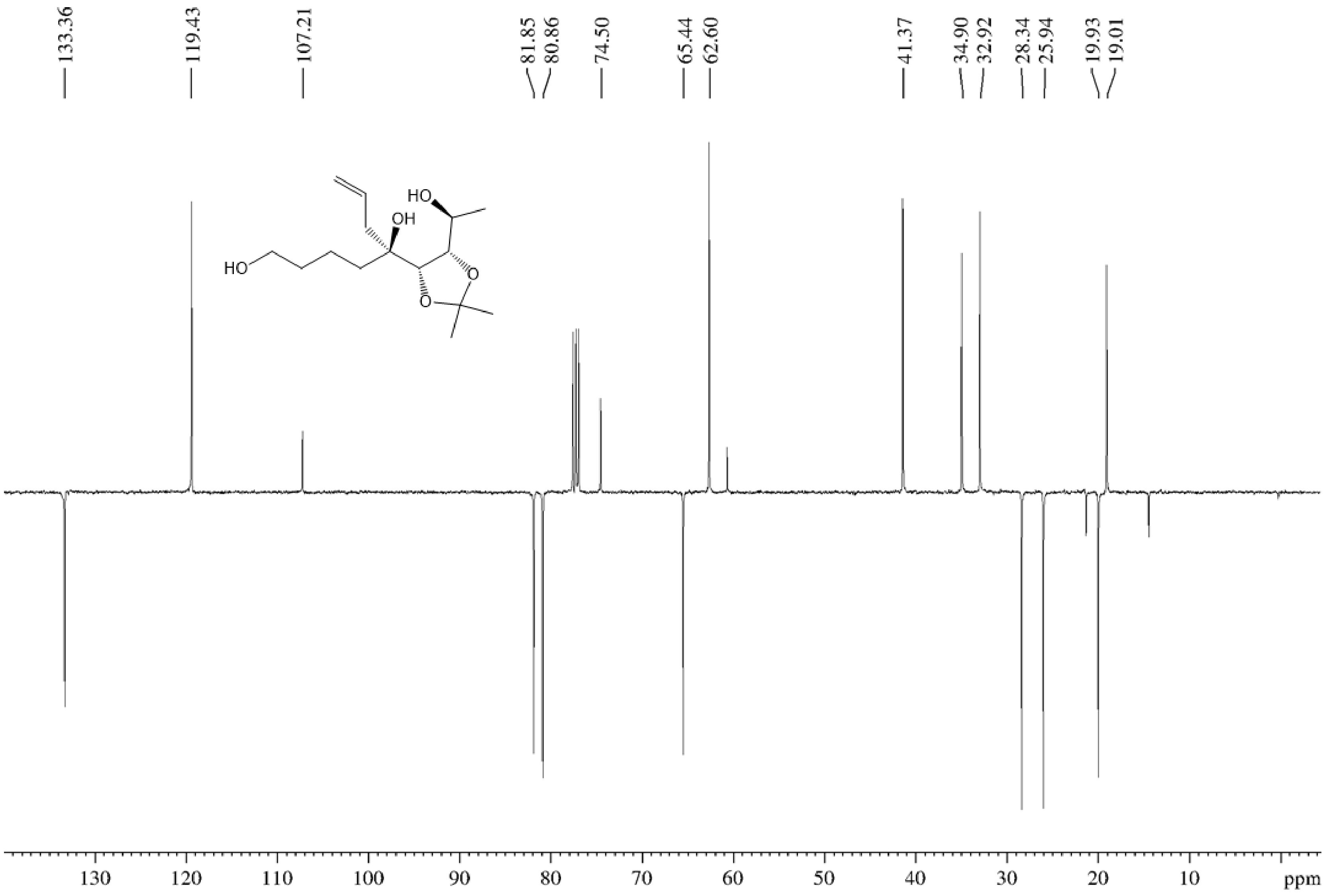


**Figure S16.** $^{13}C$ NMR spectrum of compound **12** in $CDCl_3$.

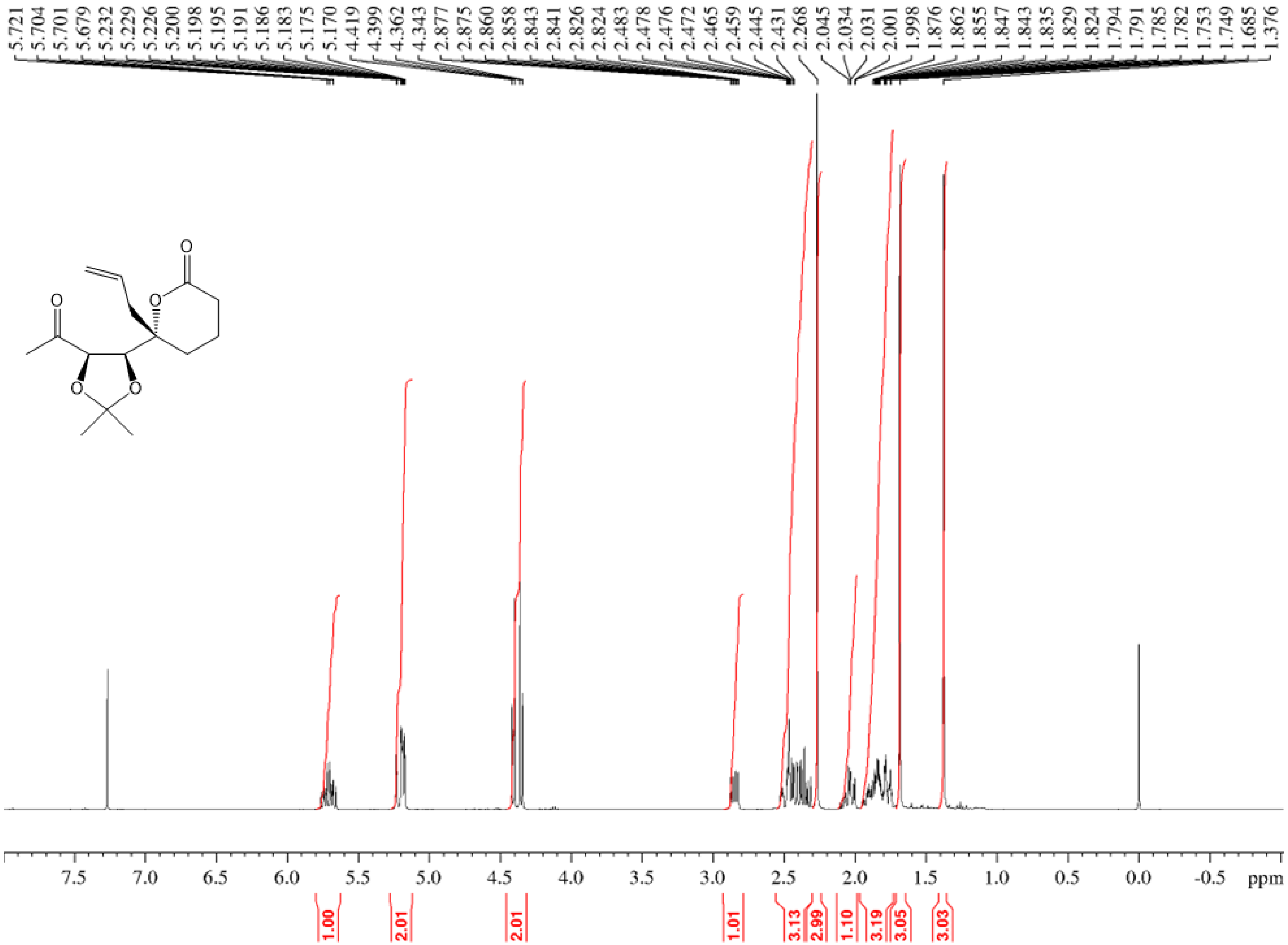

**Figure S17.** $^{1}$H NMR spectrum of compound **13** in $CDCl_3$.

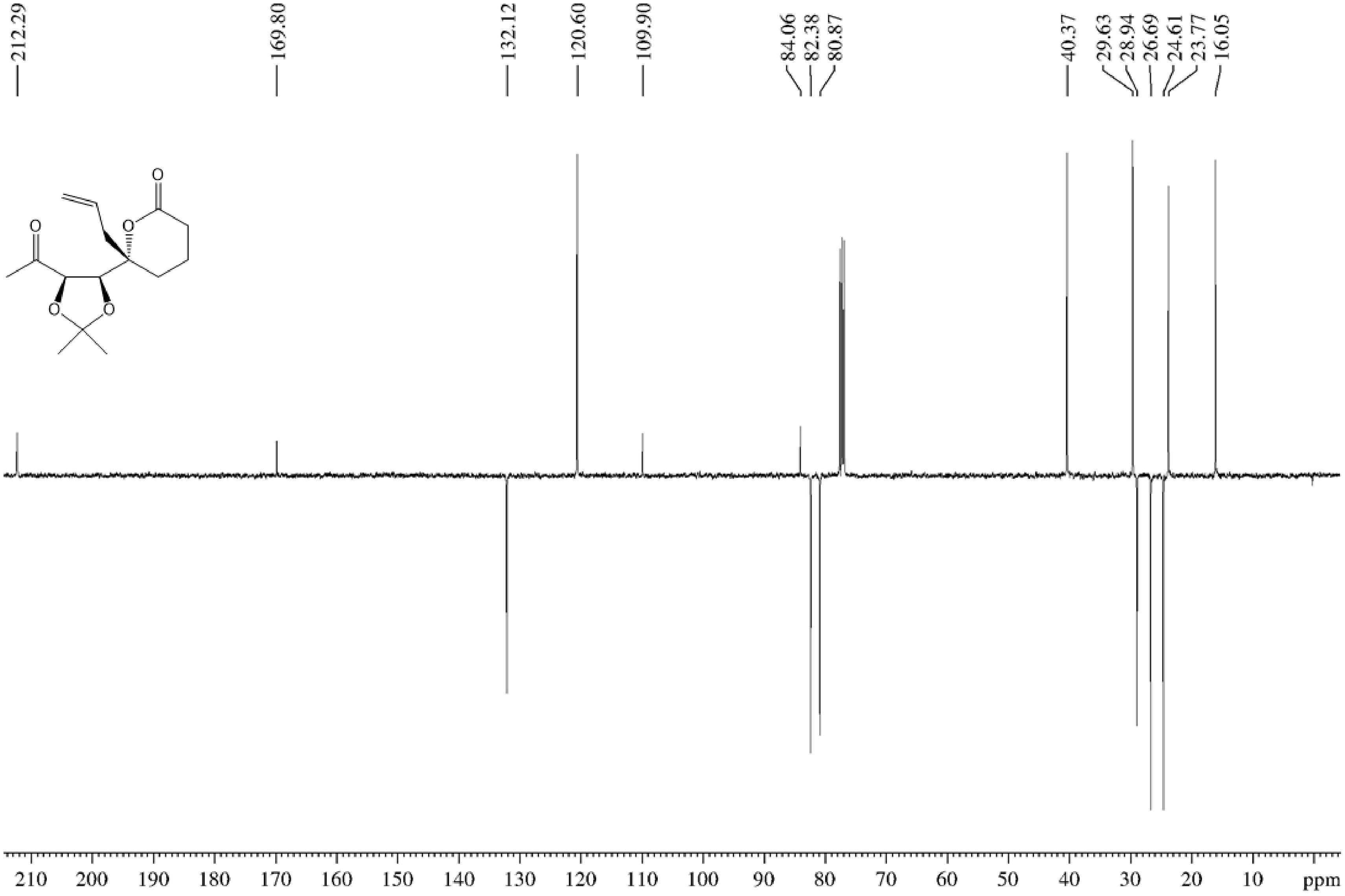


**Figure S18.** $^{13}$C NMR spectrum of compound **13** in $CDCl_3$.

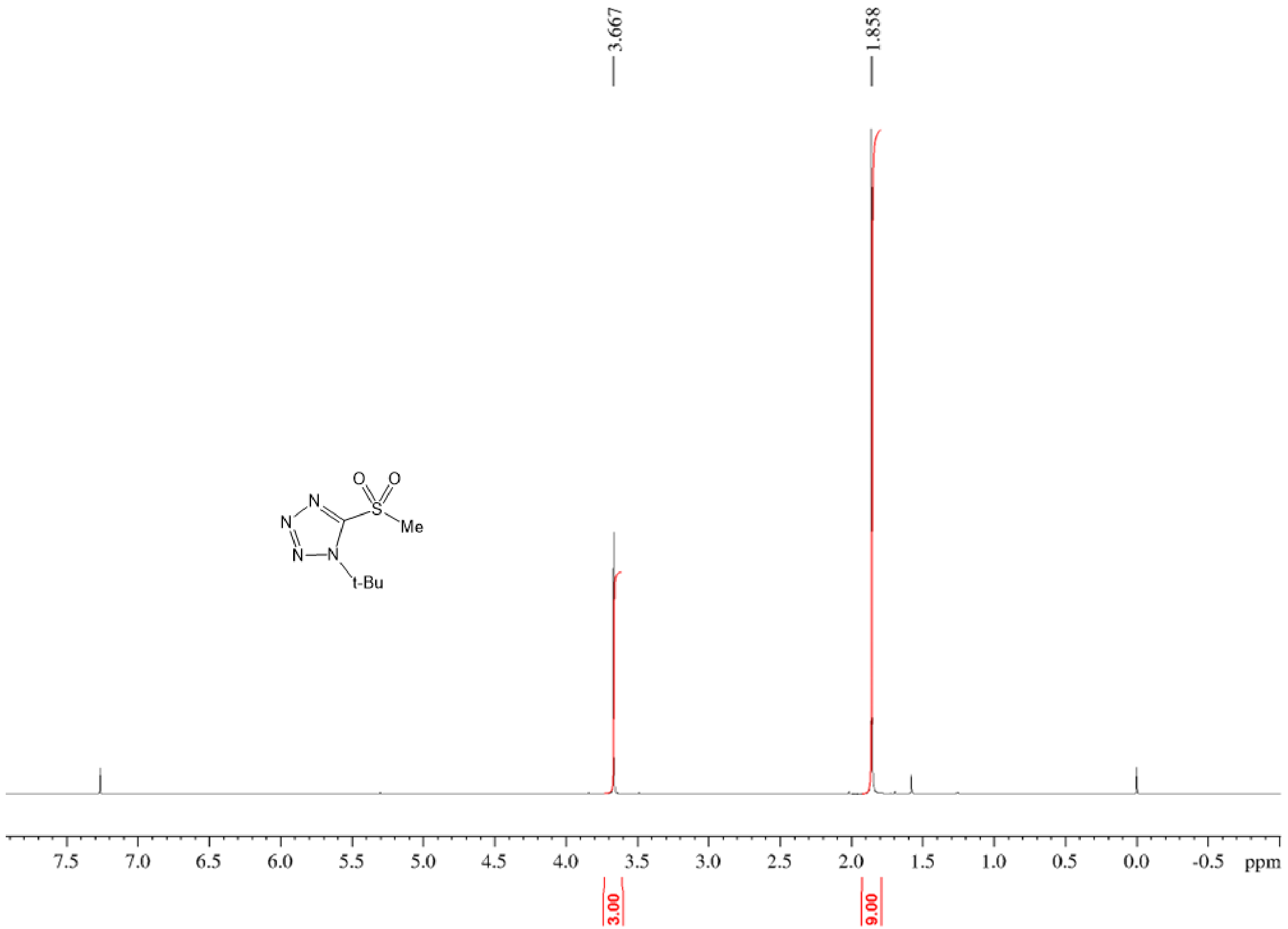


**Figure S19.** $^{1}$H NMR spectrum of compound **14** in $CDCl_3$.

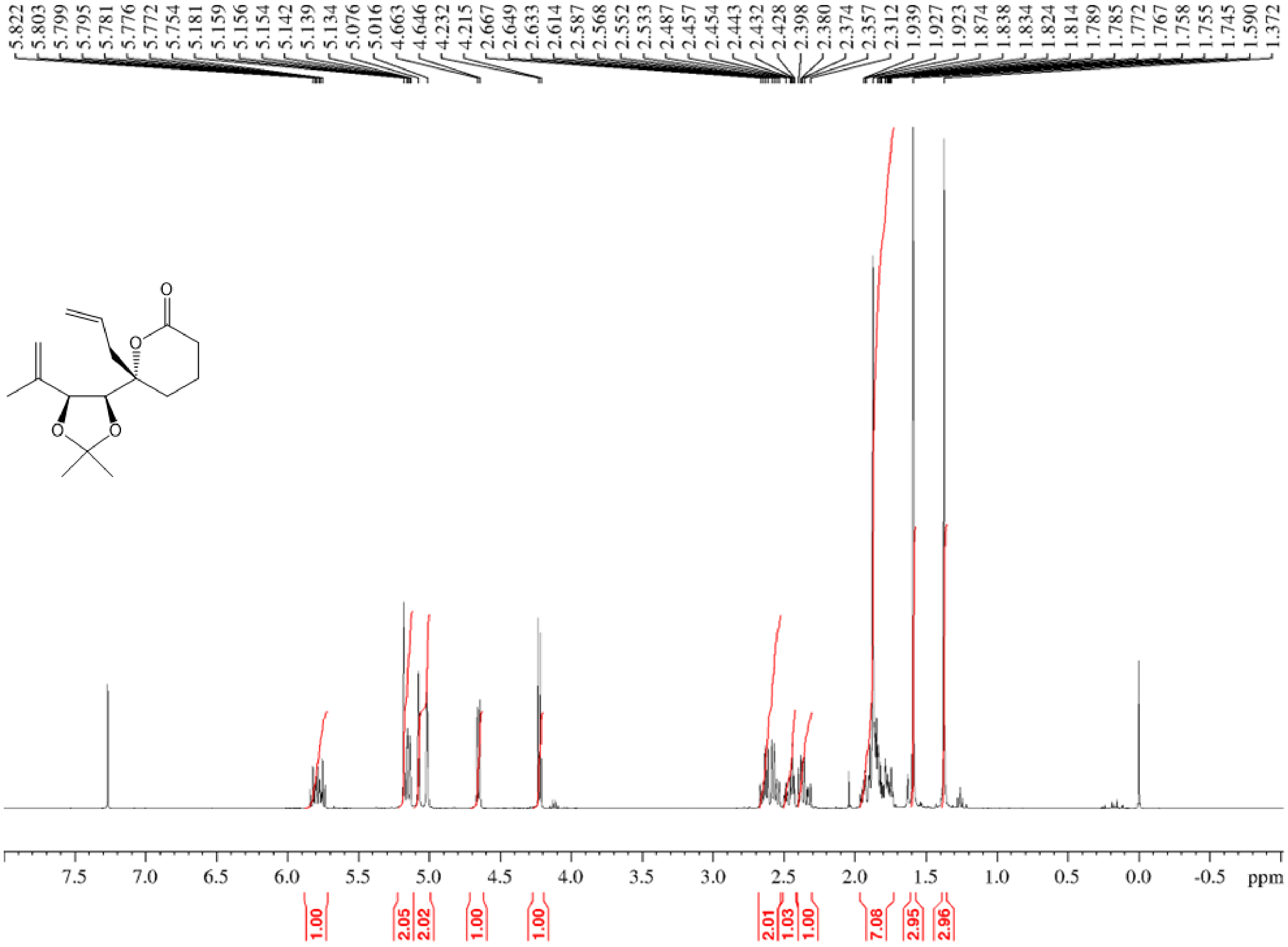

**Figure S20.** $^{1}H$ NMR spectrum of compound **15** in $CDCl_3$.

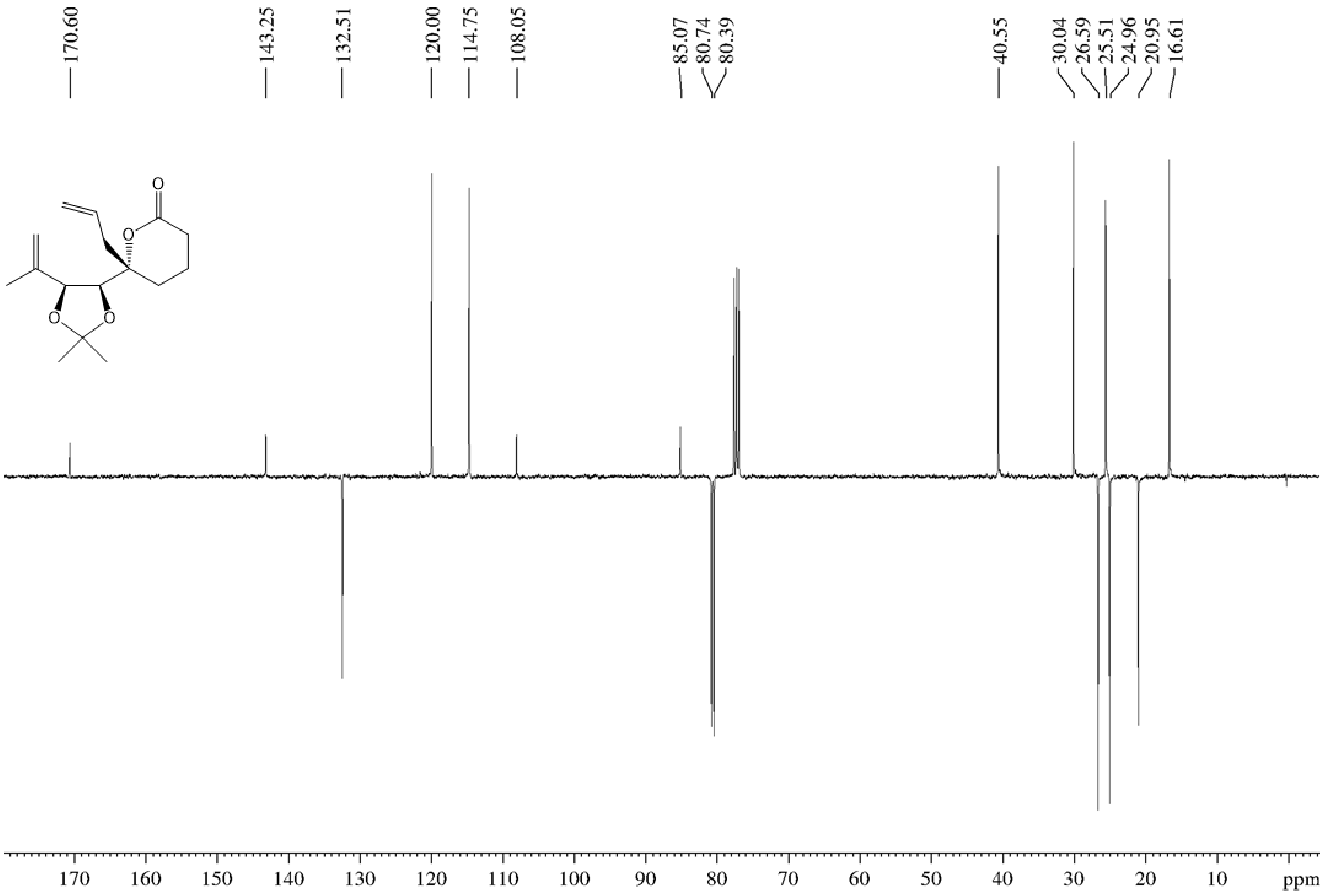


**Figure S21.** $^{13}C$ NMR spectrum of compound **15** in $CDCl_3$.

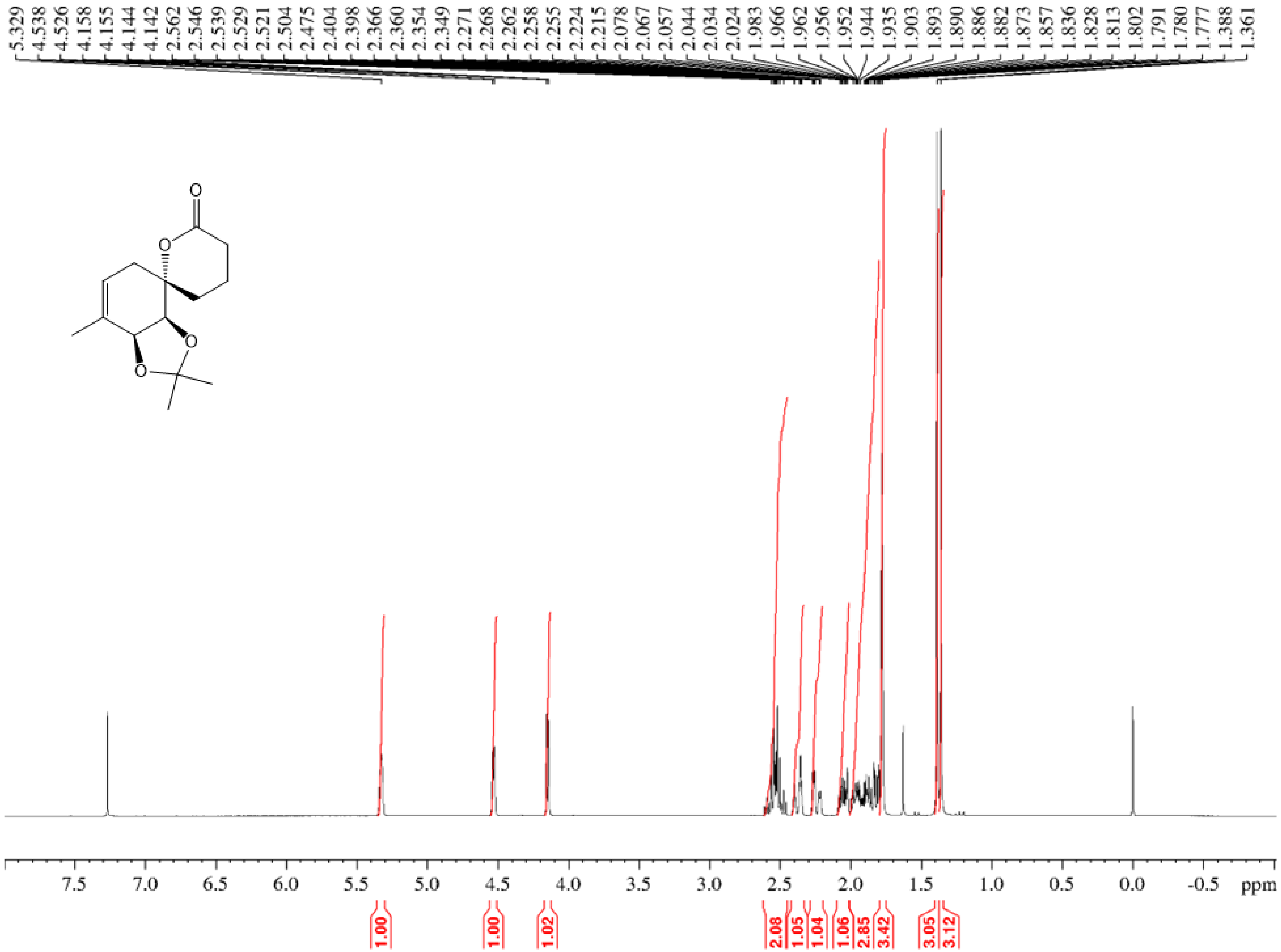

**Figure S22.** $^{1}$H NMR spectrum of compound **16** in $CDCl_3$.

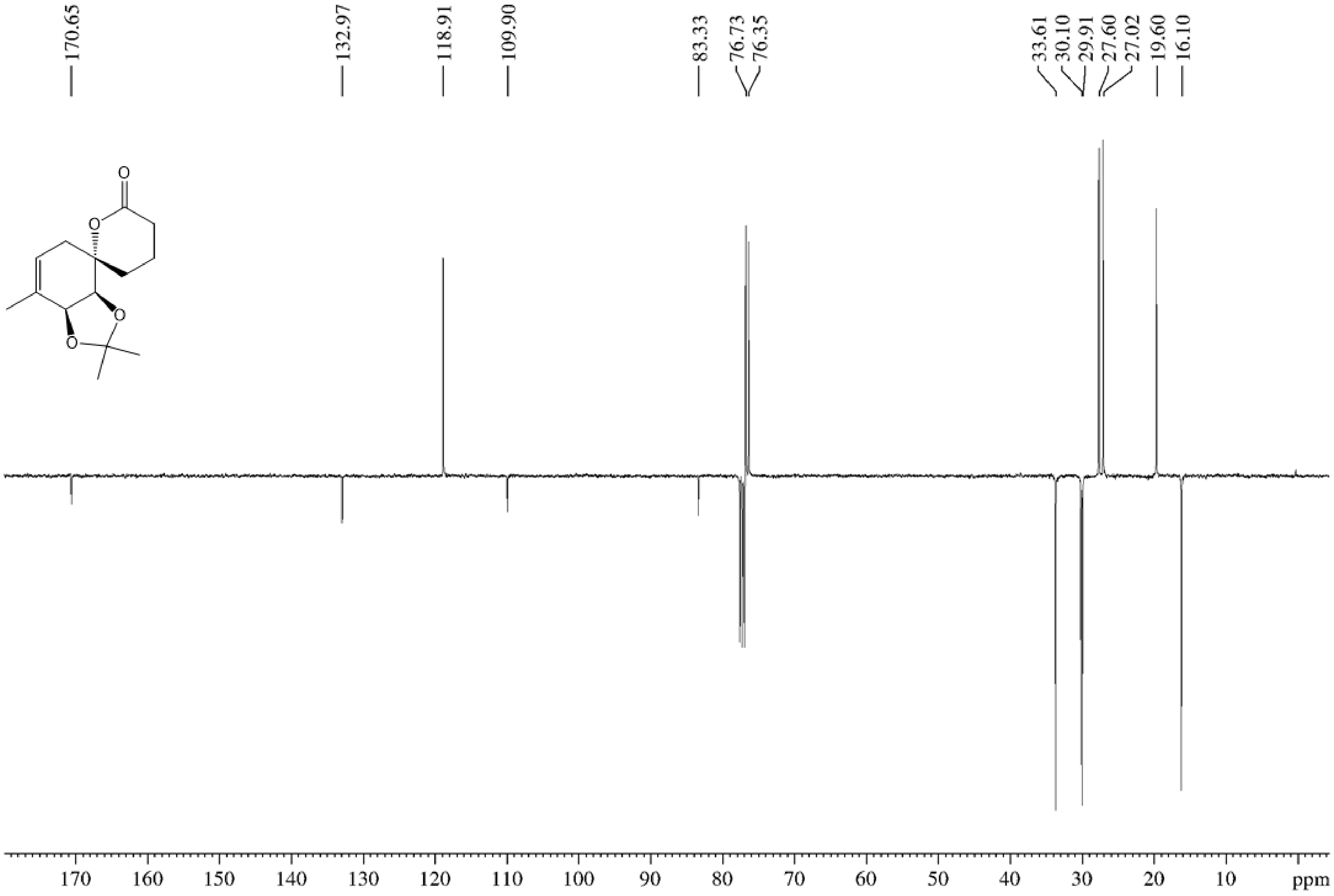


**Figure S23.** $^{13}$C NMR spectrum of compound **16** in $CDCl_3$.

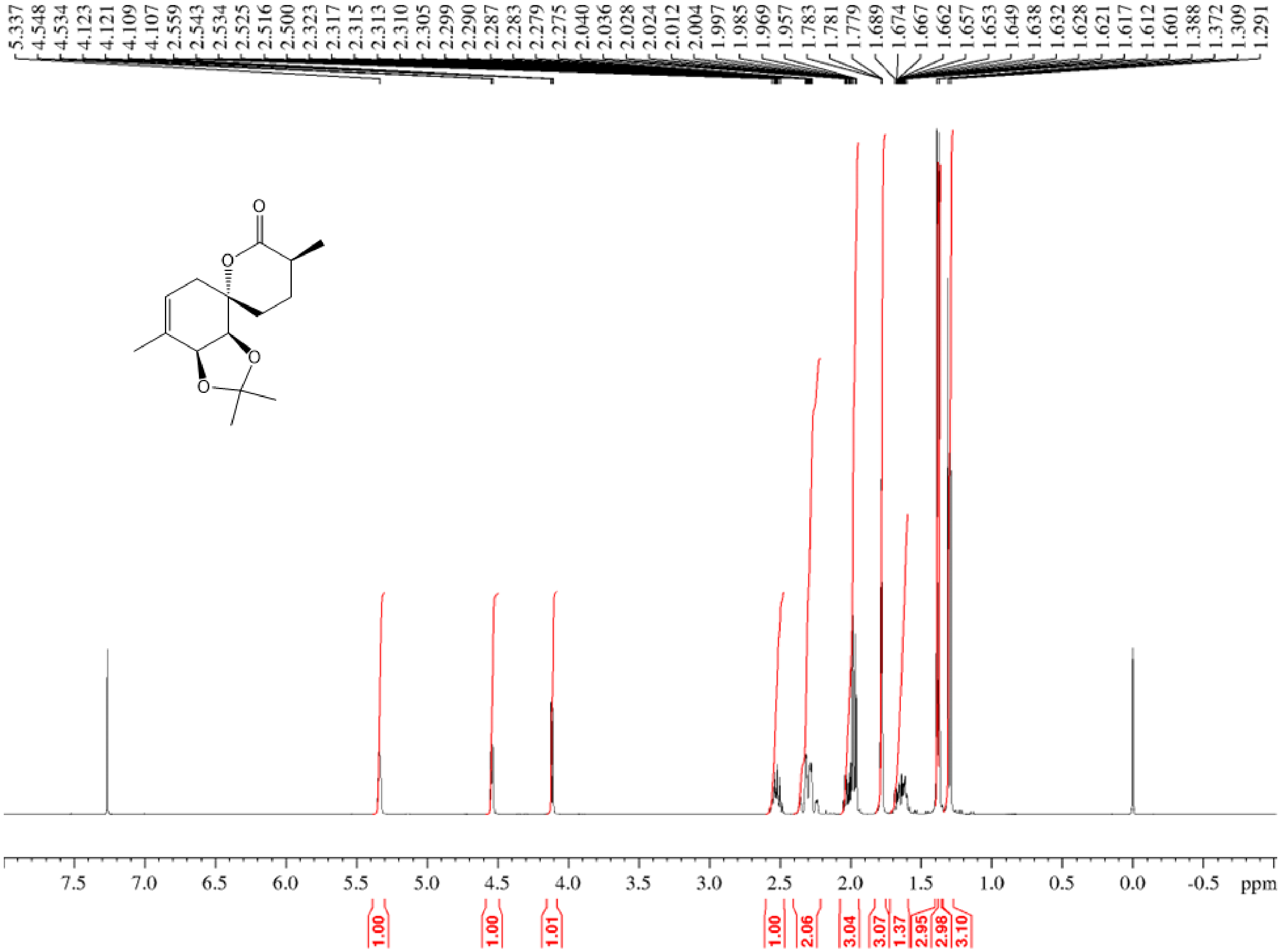

**Figure S24.** $^{1}$H NMR spectrum of compound **17** in $CDCl_3$.

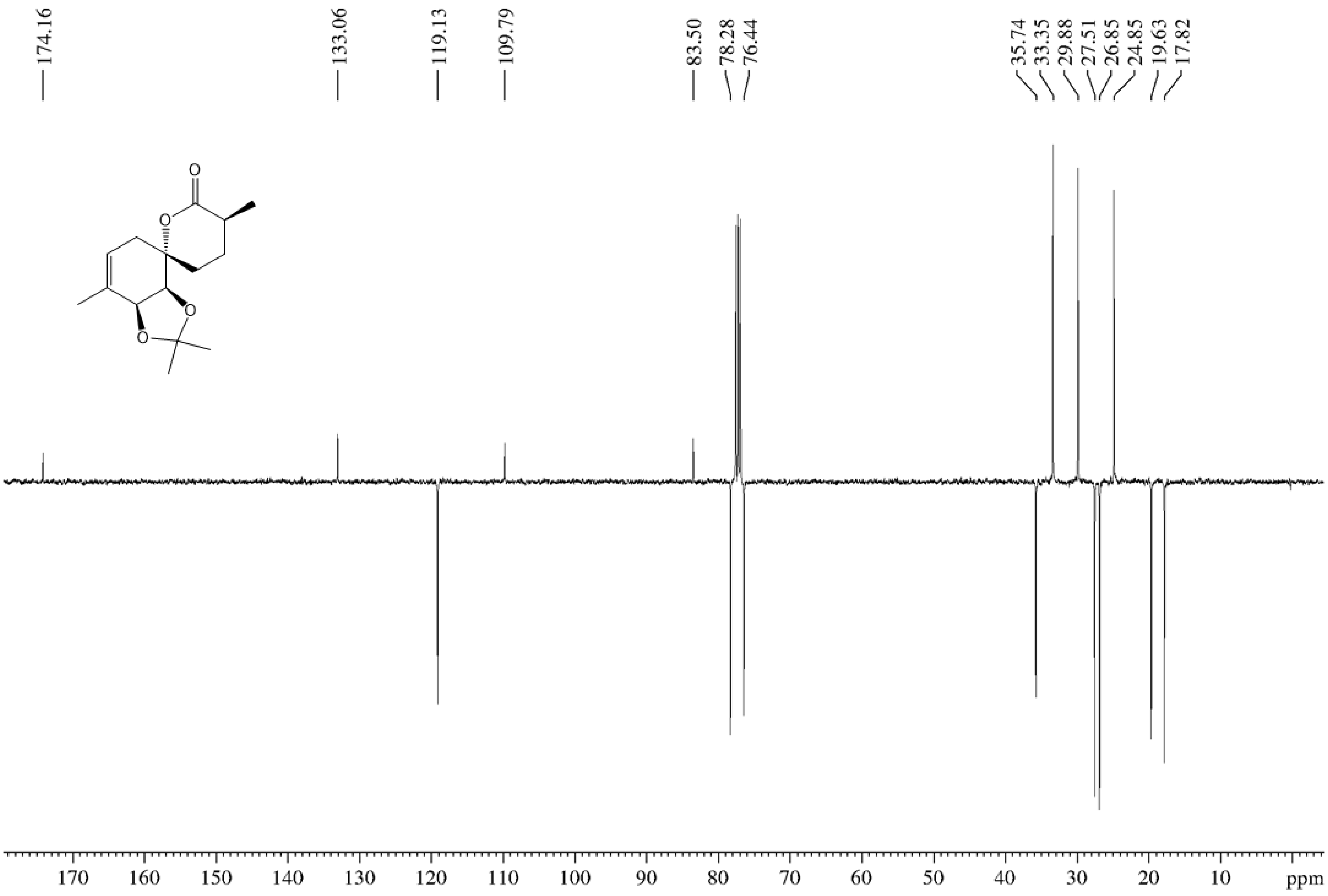


**Figure S25.** $^{13}C$ NMR spectrum of compound **17** in $CDCl_3$.

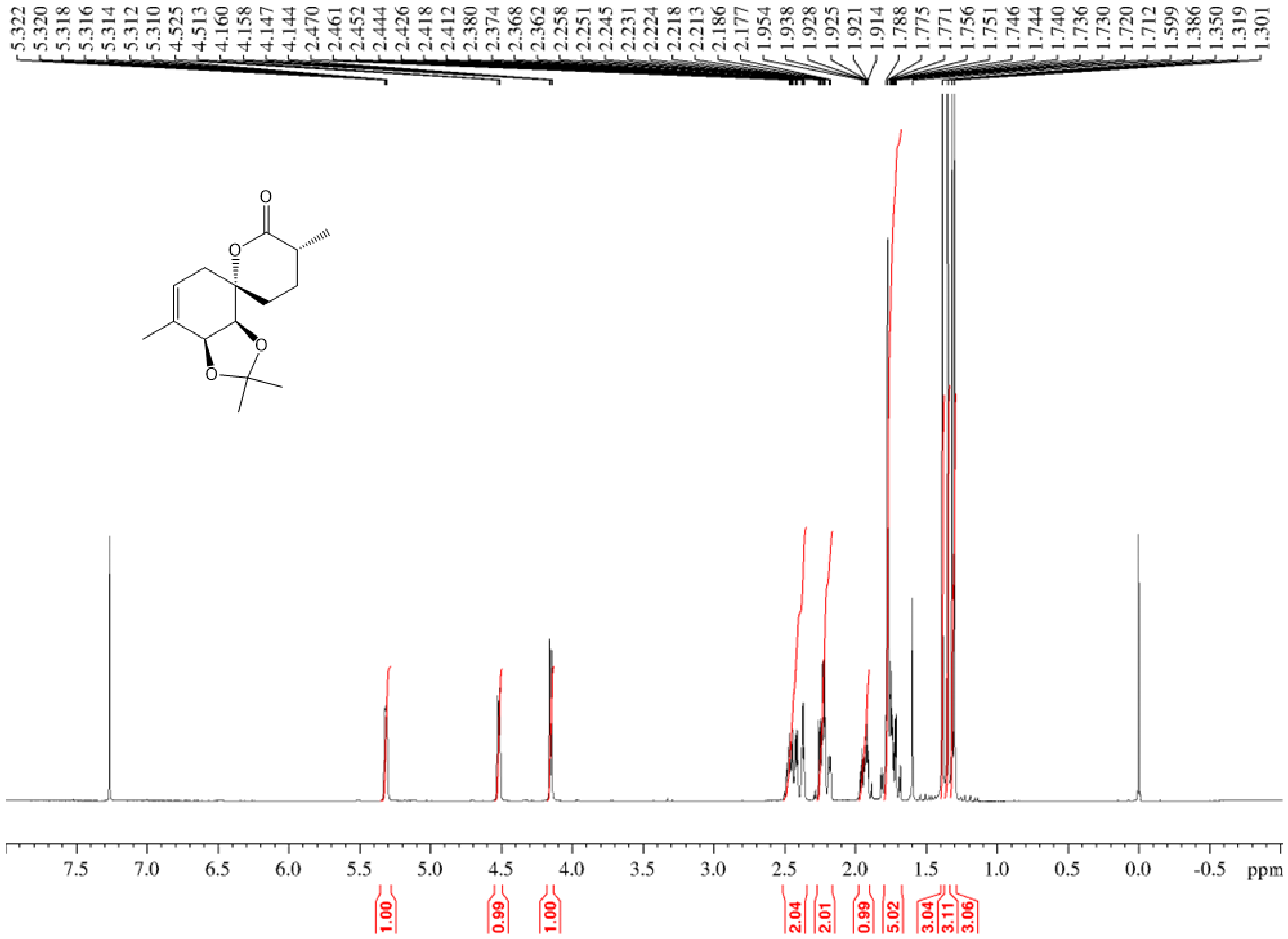

**Figure S26.** $^{1}$H NMR spectrum of compound **18** in $CDCl_3$.

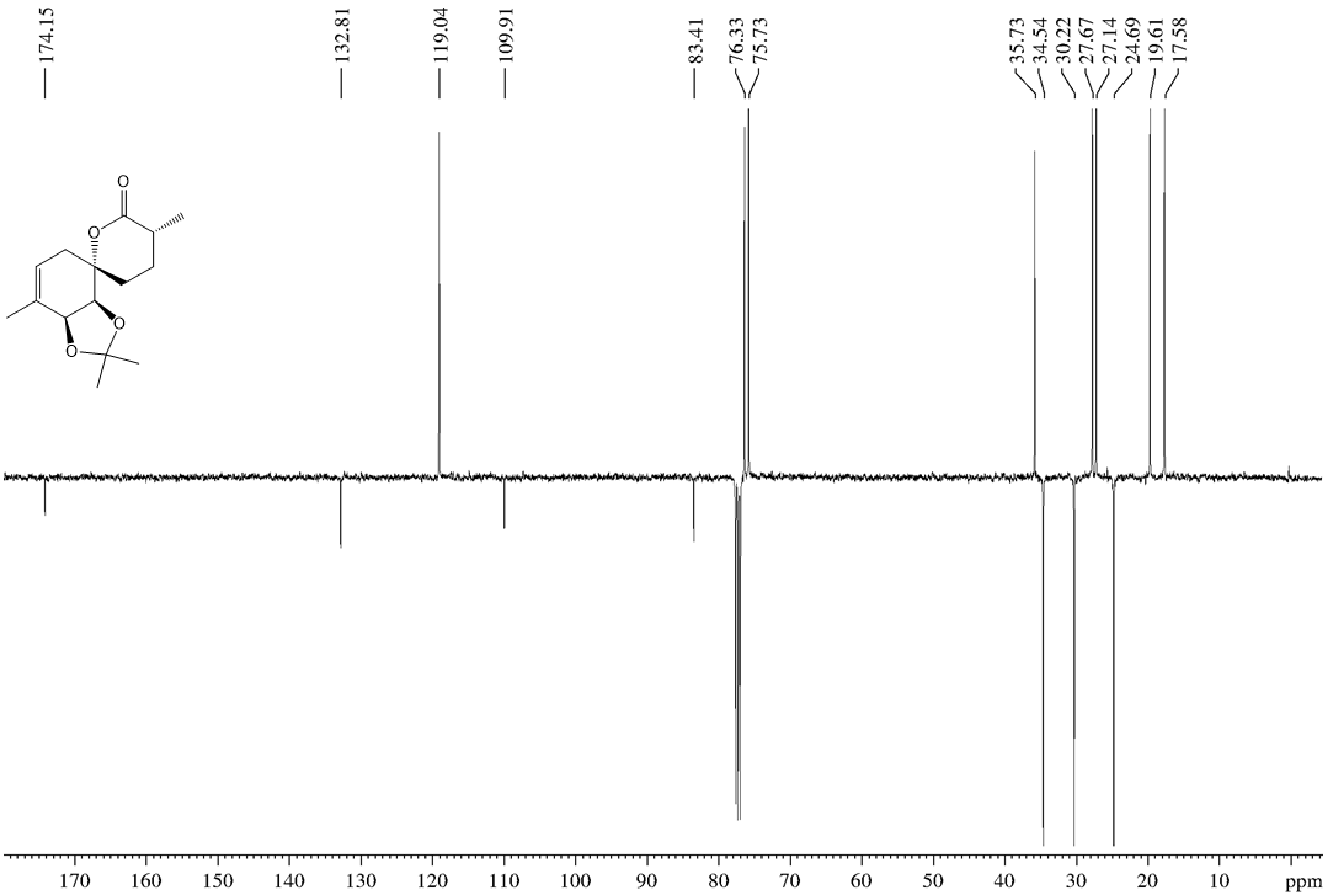


**Figure S27.** $^{13}$C NMR spectrum of compound **18** in $CDCl_3$.

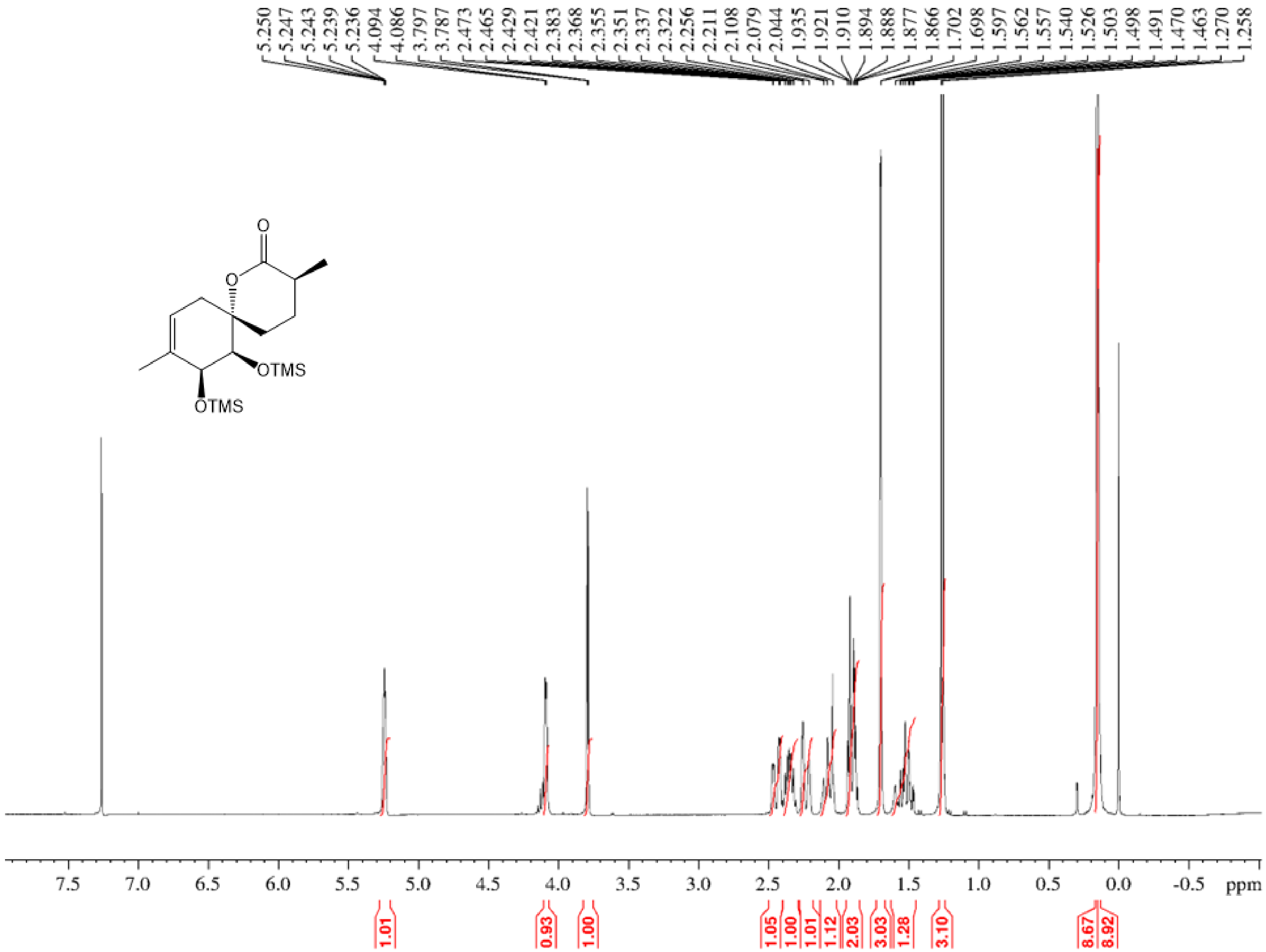


**Figure S28.** $^{1}H$ NMR spectrum of compound **19** in $CDCl_3$.

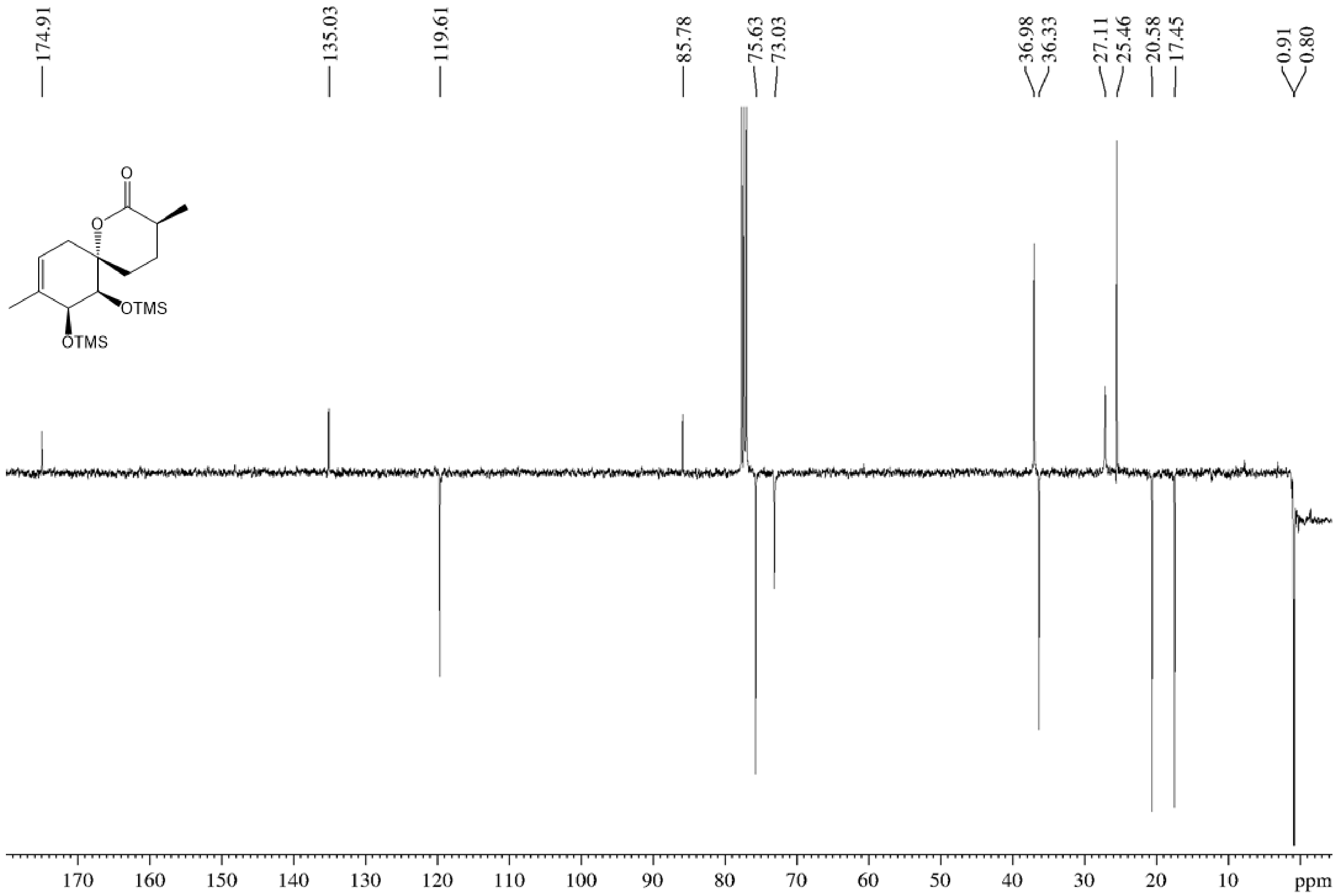


**Figure S29.** $^{13}C$ NMR spectrum of compound **19** in $CDCl_3$.

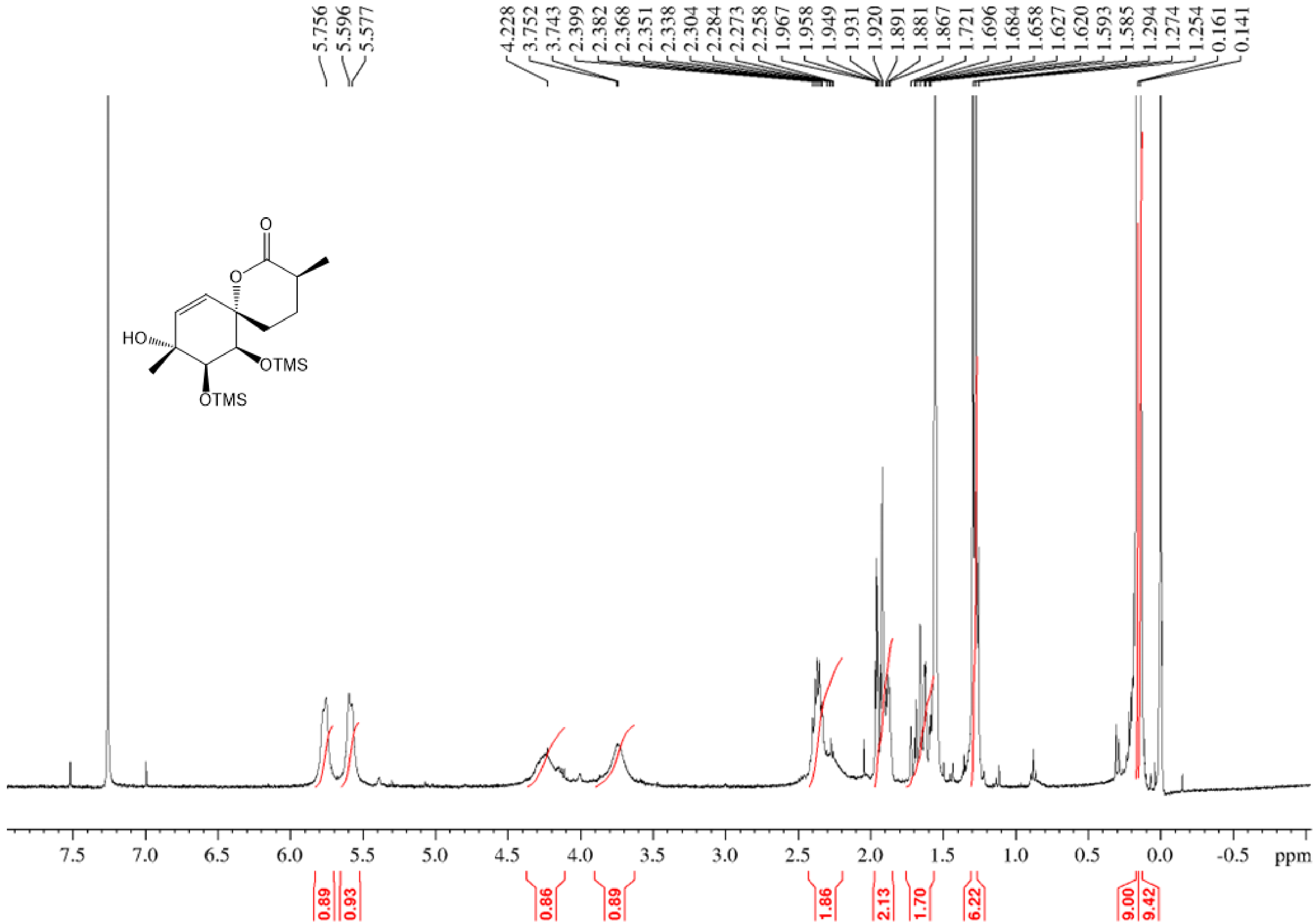


**Figure S30.** $^{1}$H NMR spectrum of compound **20** in $CDCl_3$.

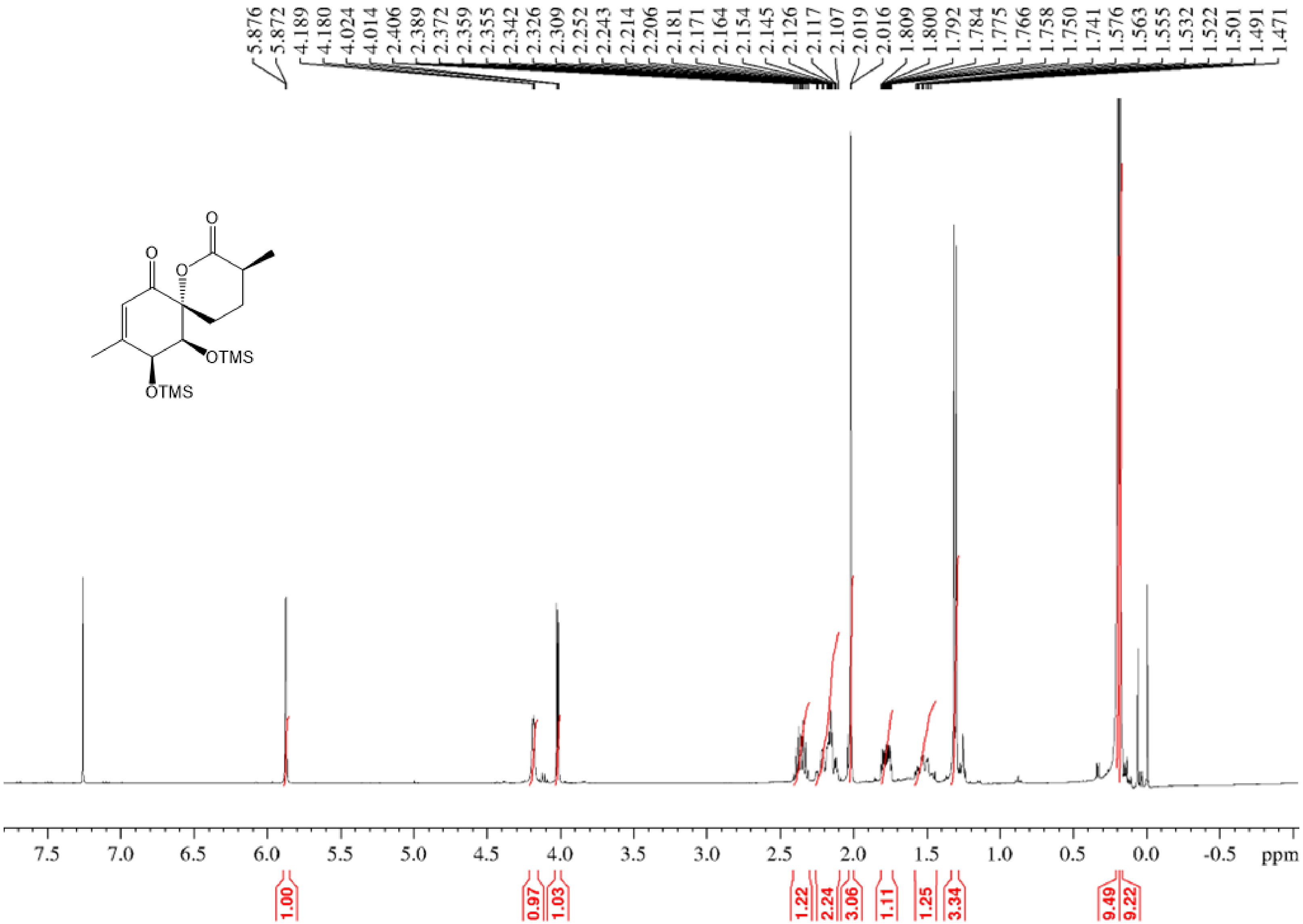


**Figure S31.** $^1$H NMR spectrum of compound **21** in $CDCl_3$.

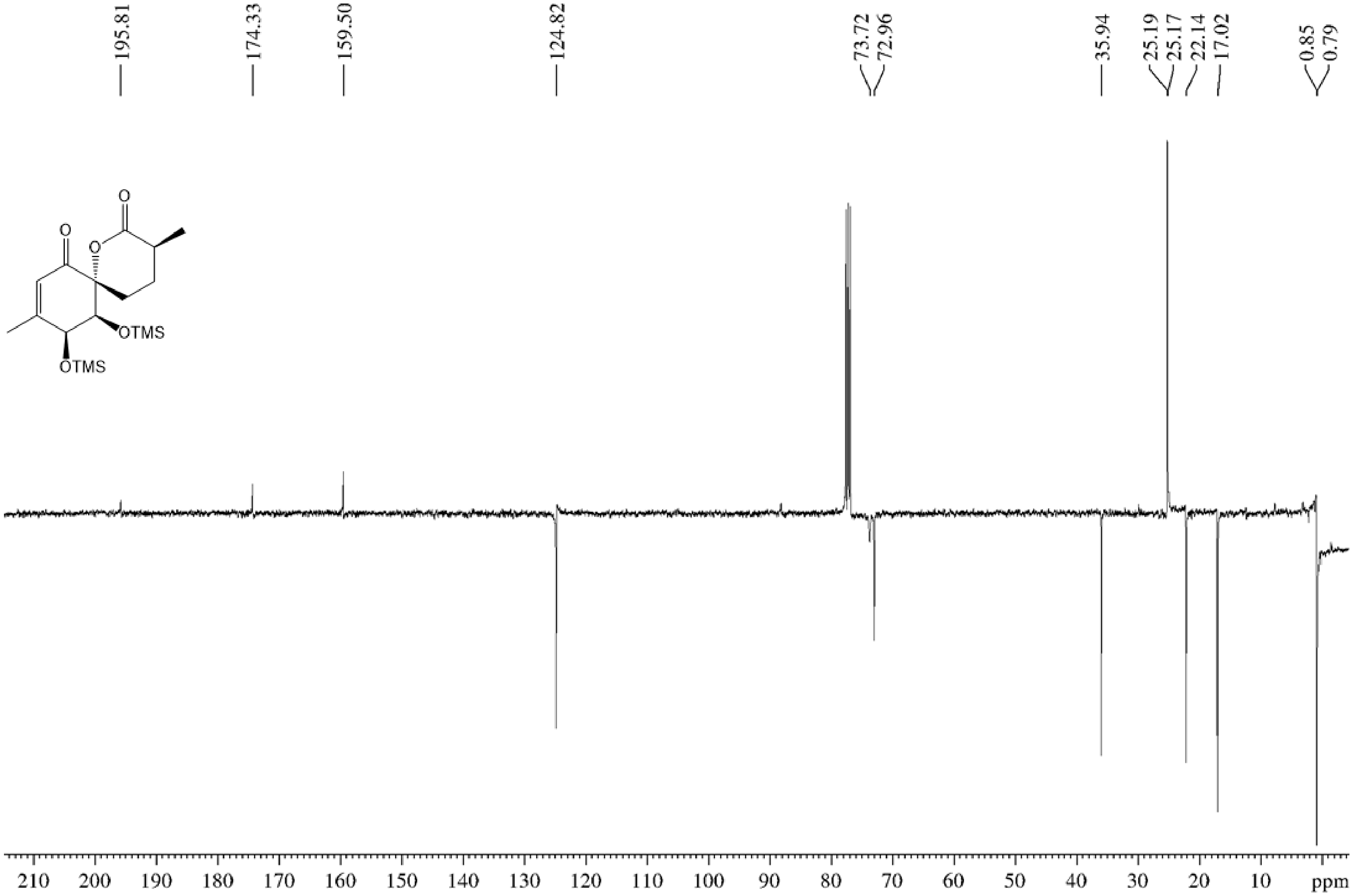


**Figure S32.** $^{13}C$ NMR spectrum of compound **21** in $CDCl_3$.

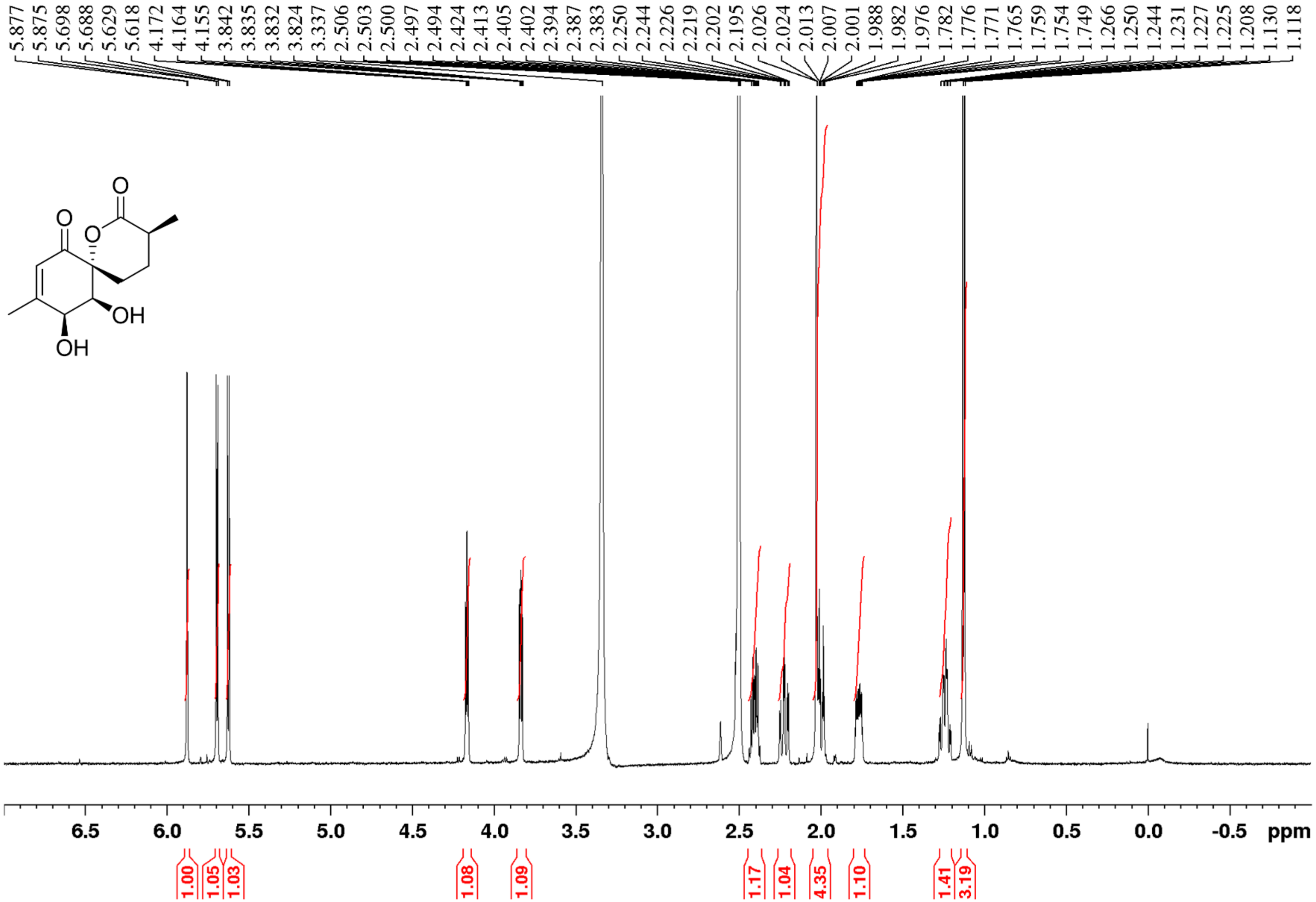


**Figure S33.** $^{1}$H NMR spectrum of compound (+)-**1** in DMSO-d$_6$.

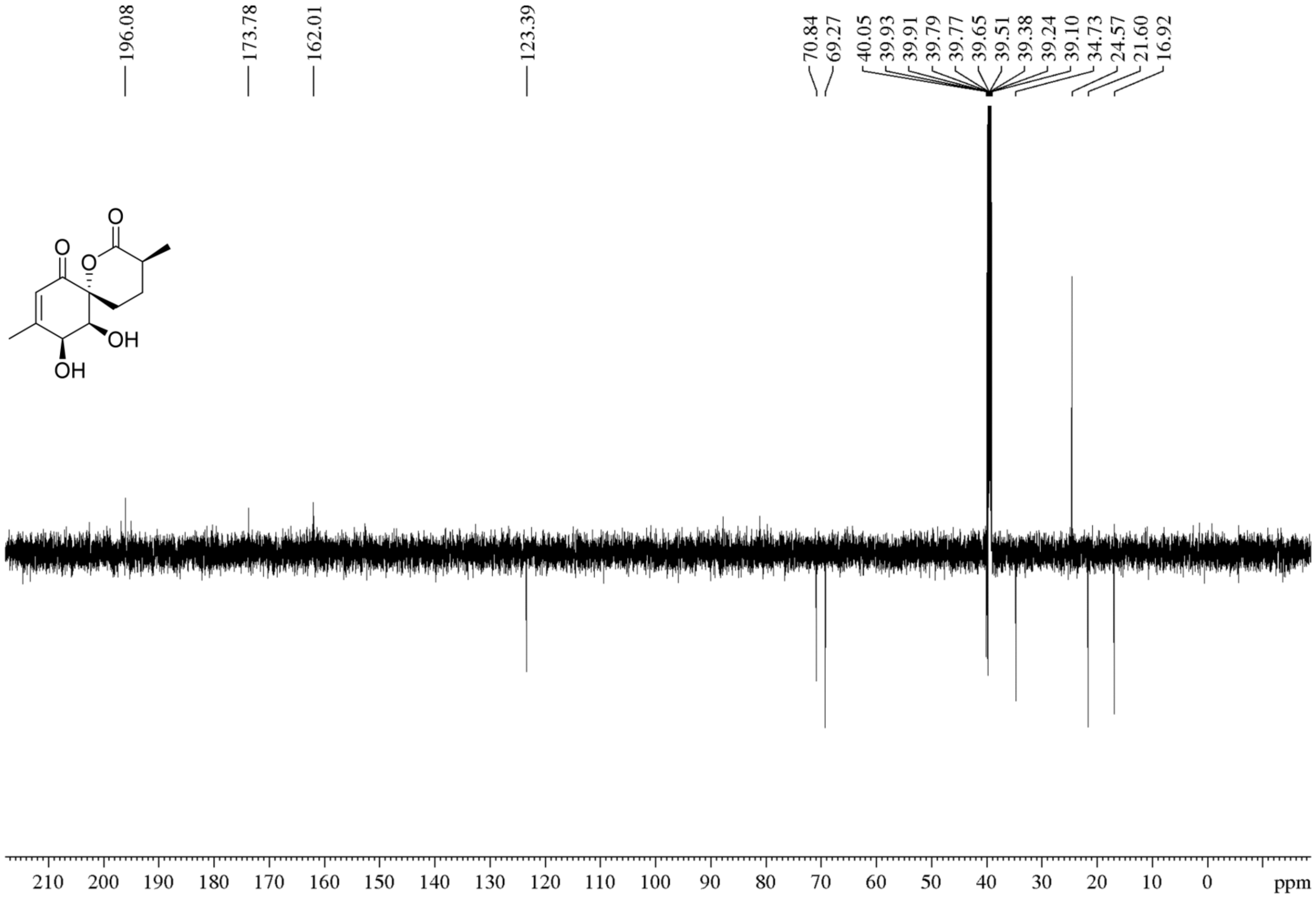


**Figure S34.** $^{13}C$ NMR spectrum of compound (+)-**1** in DMSO-$d_6$.

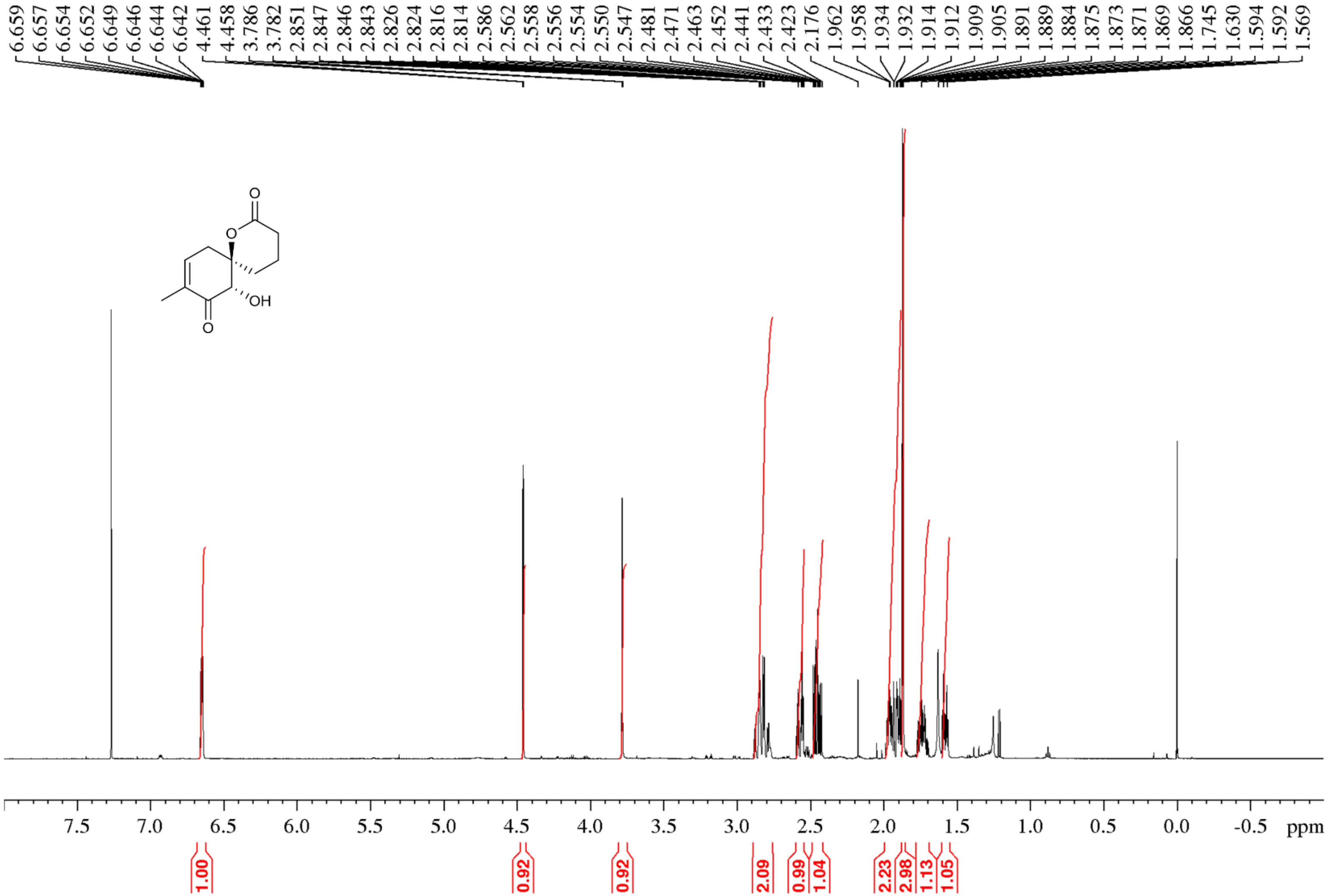


**Figure S35.** $^{1}$H NMR spectrum of compound **22** in $CDCl_3$.

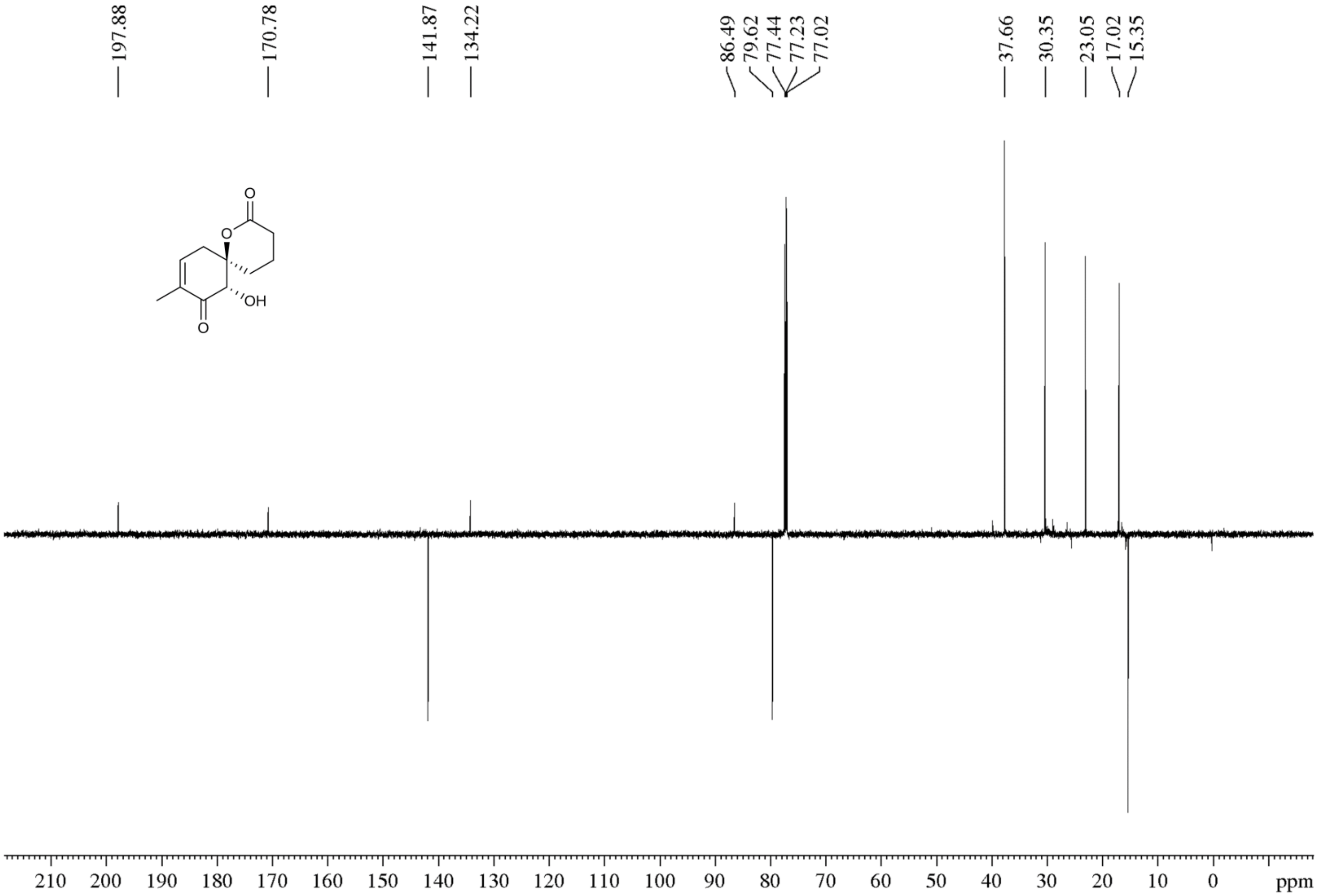


**Figure S36.** $^{13}$C NMR spectrum of compound **22** in $CDCl_3$.

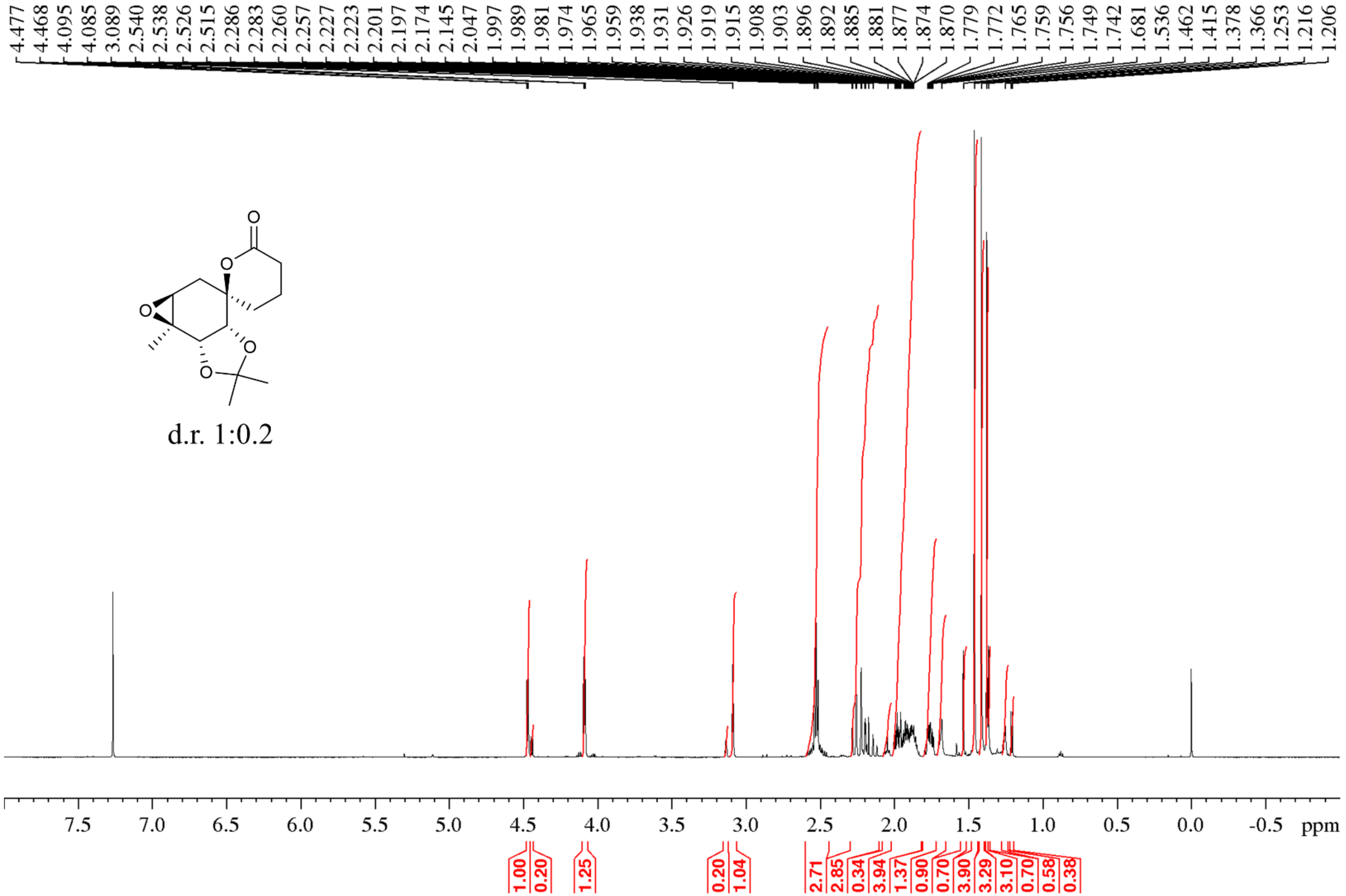

**Figure S37.** $^{1}$H NMR spectrum of compound **23** in $CDCl_3$.

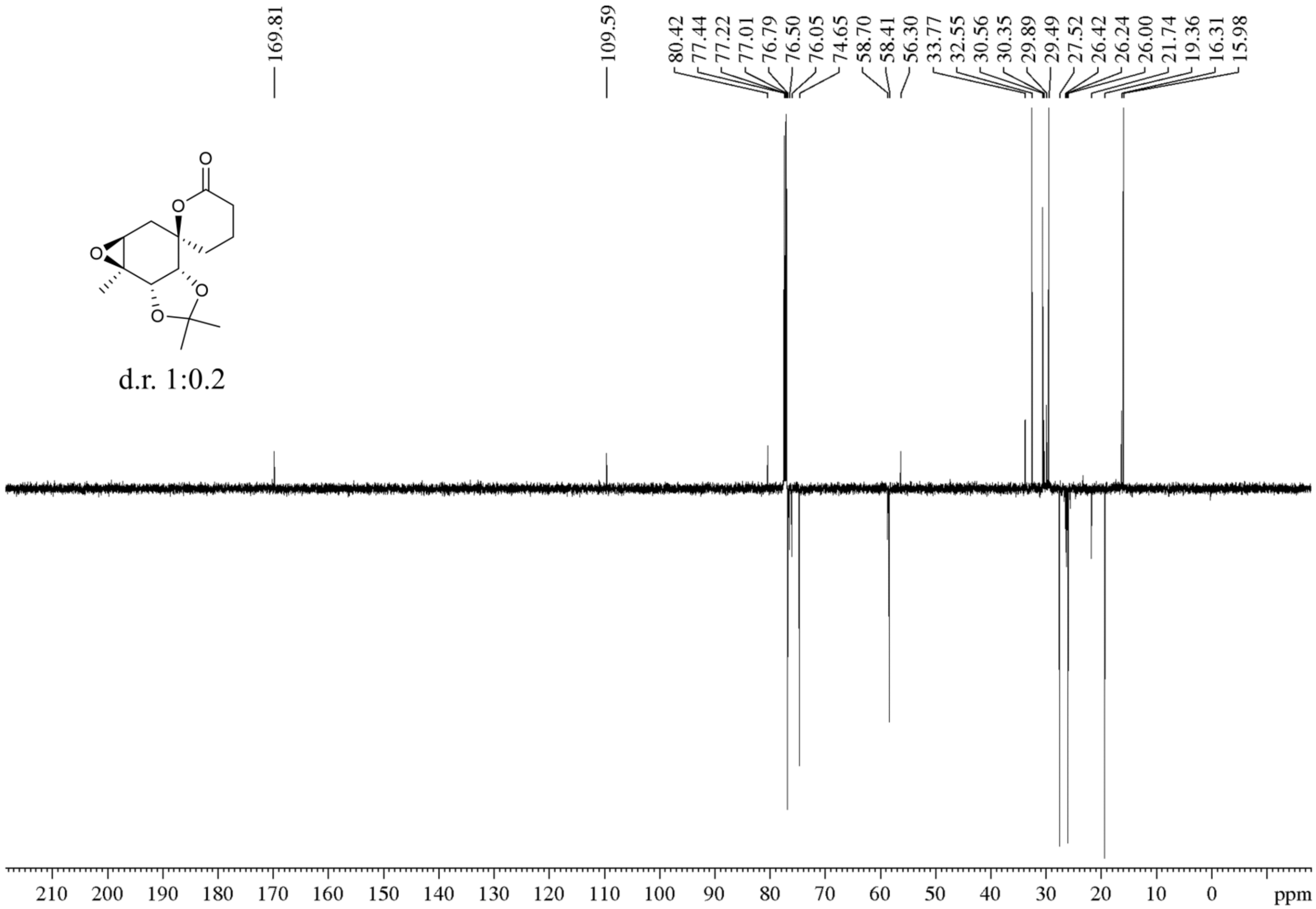


**Figure S38.** $^{13}C$ NMR spectrum of compound **23** in $CDCl_3$.

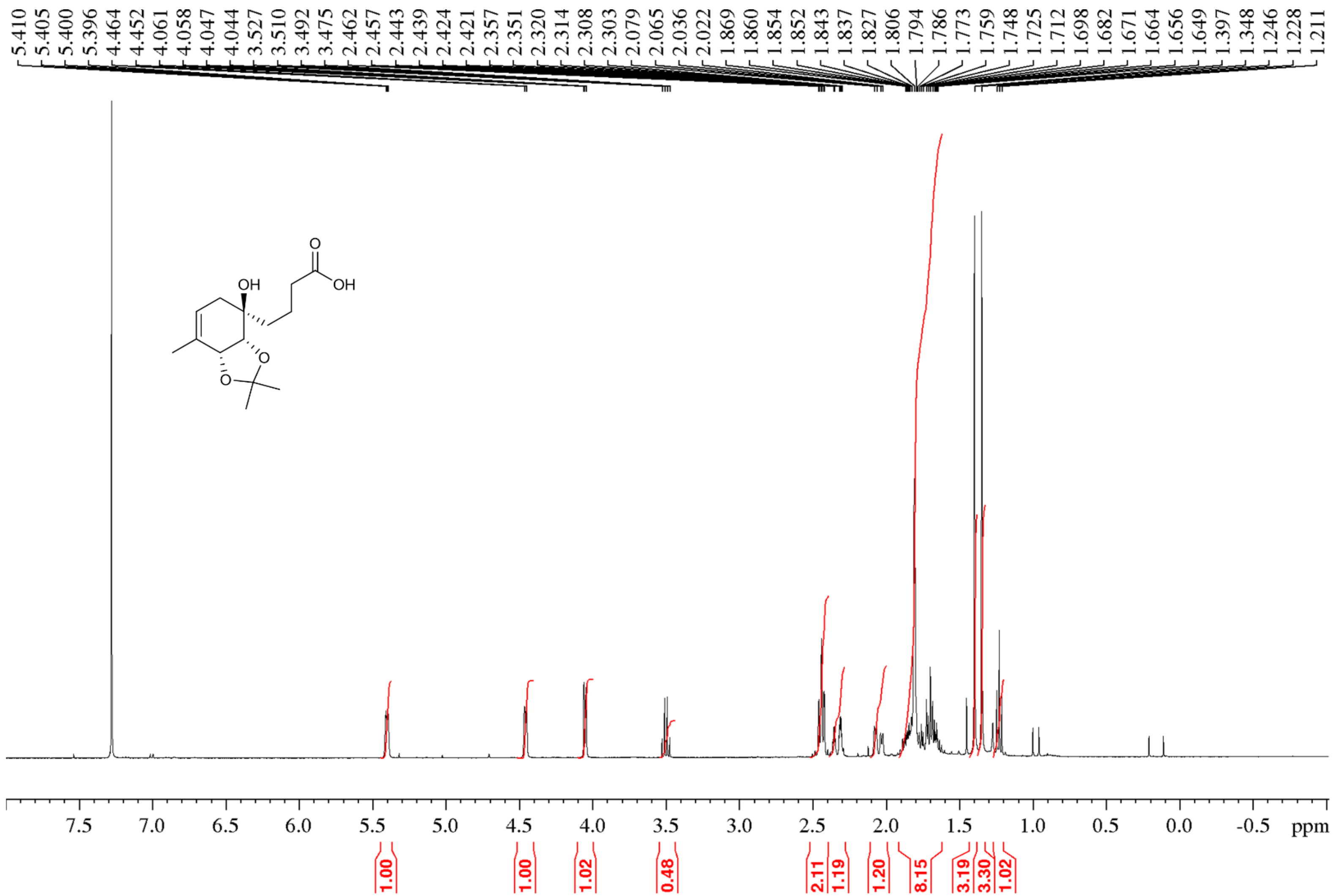

**Figure S39.** $^{1}H$ NMR spectrum of compound **24** in $CDCl_3$.

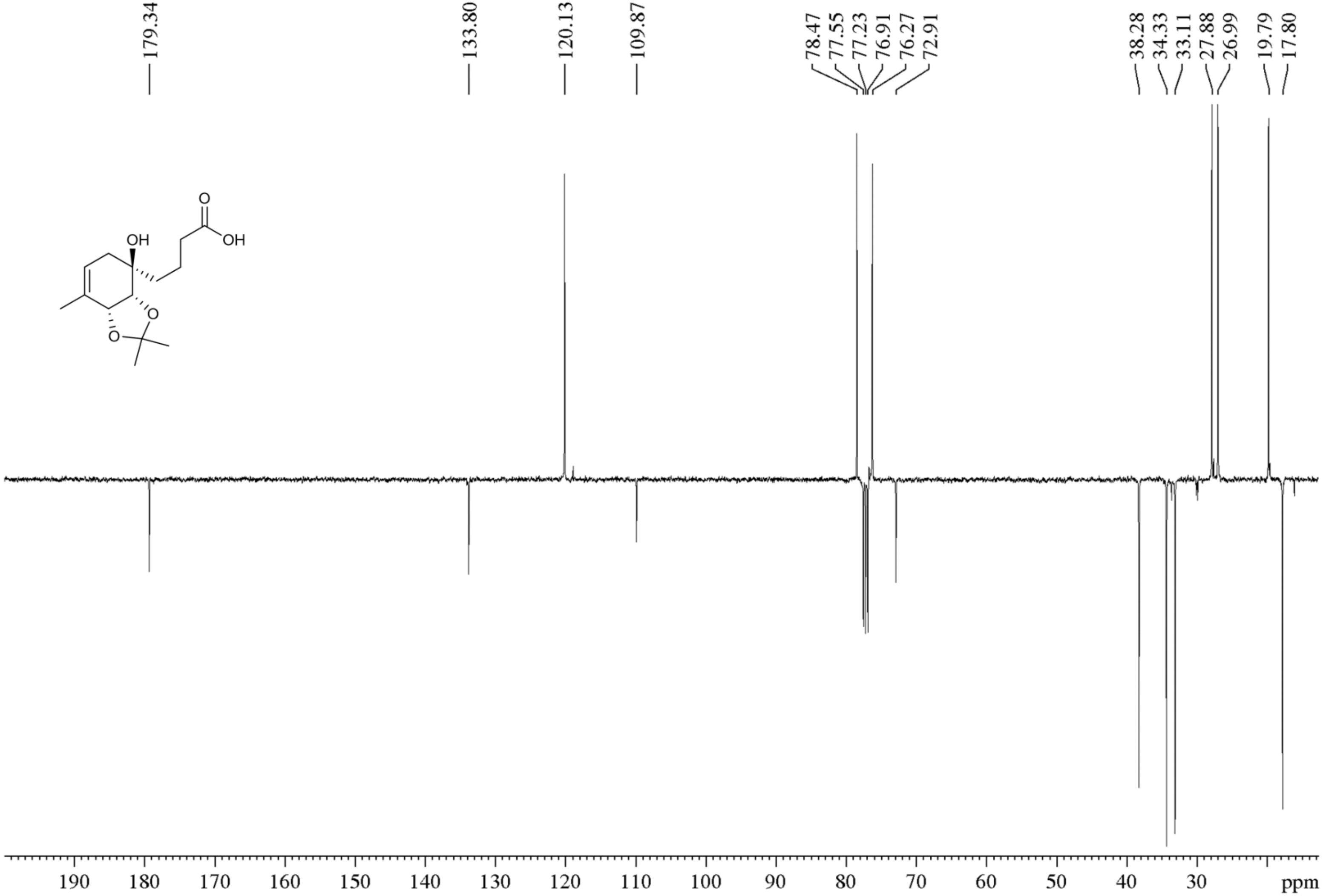


**Figure S40.** $^{13}$C NMR spectrum of compound **24** in $CDCl_3$.

## 3. HRMS spectra

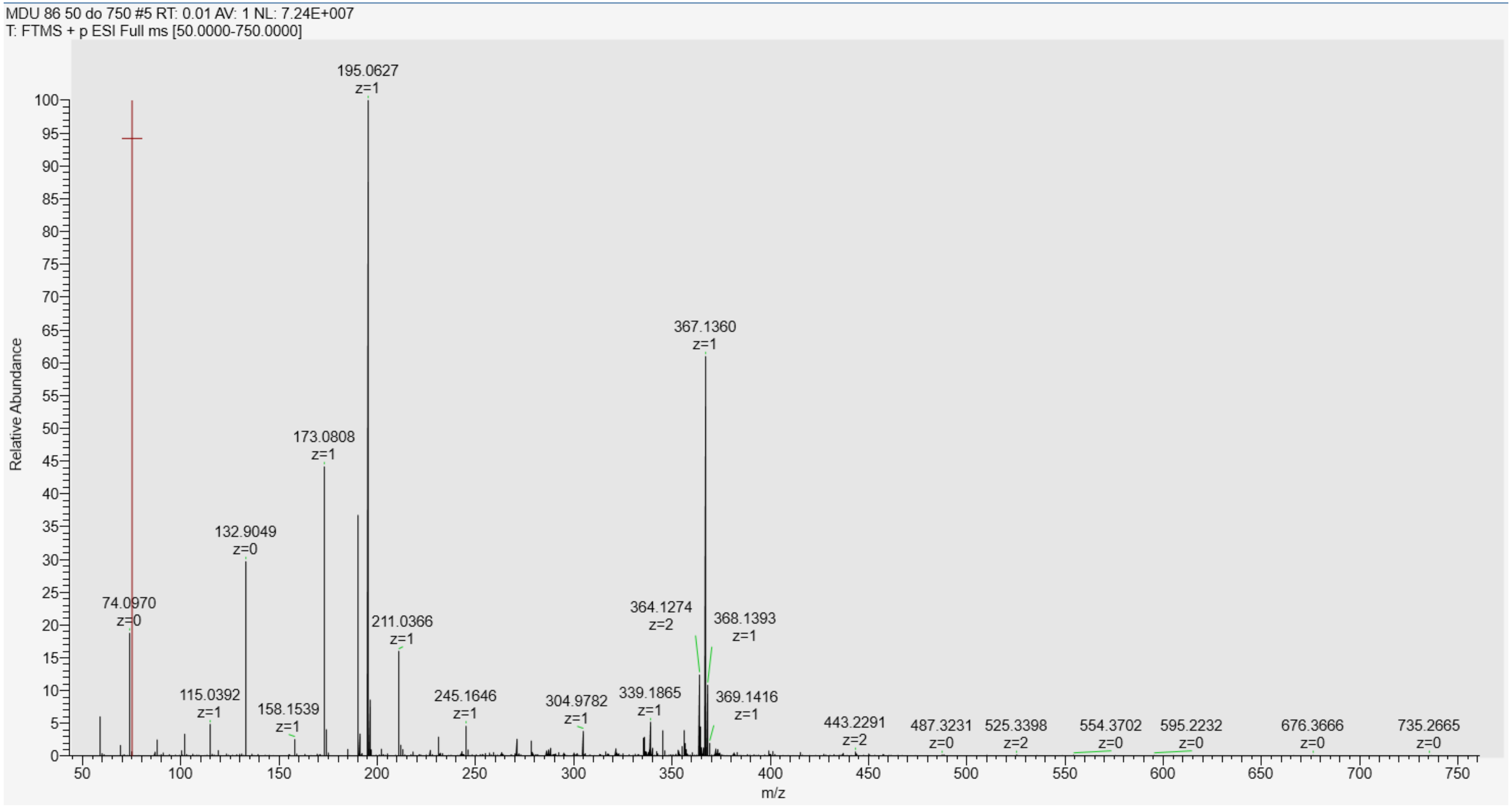


**Figure S41.** HRMS spectrum of compound **9**.

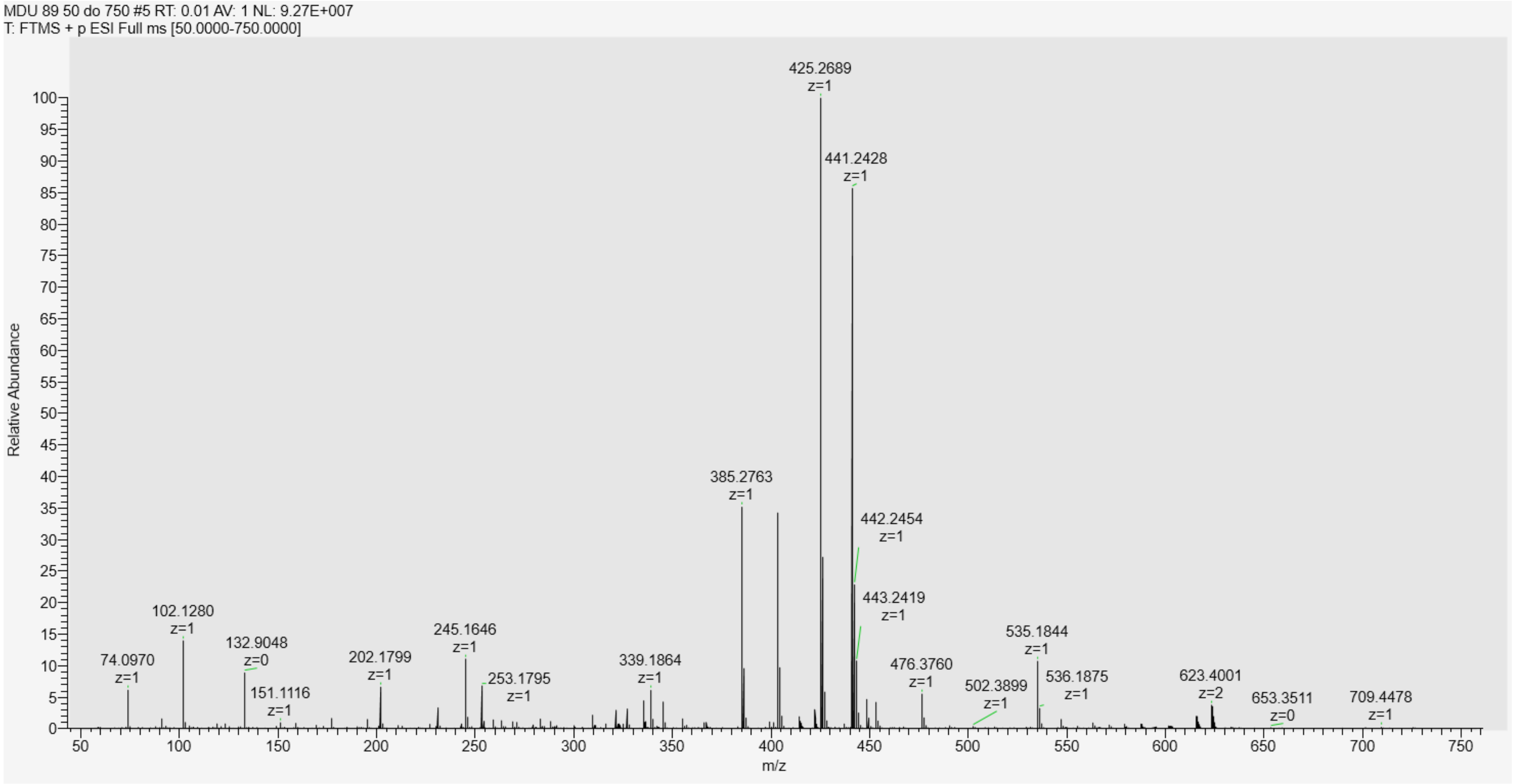


**Figure S42.** HRMS spectrum of compound **11**.

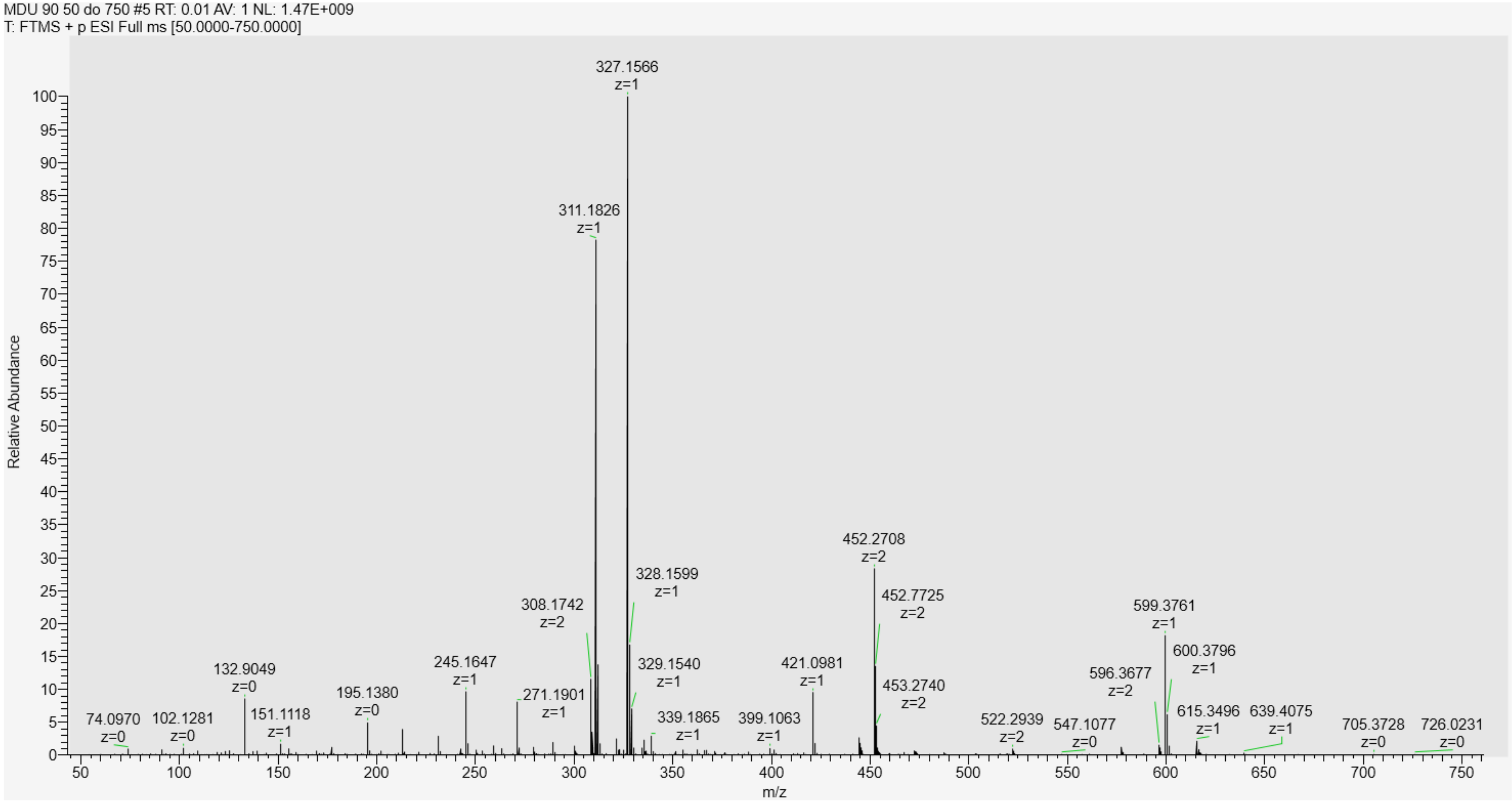


**Figure S43.** HRMS spectrum of compound **12**.

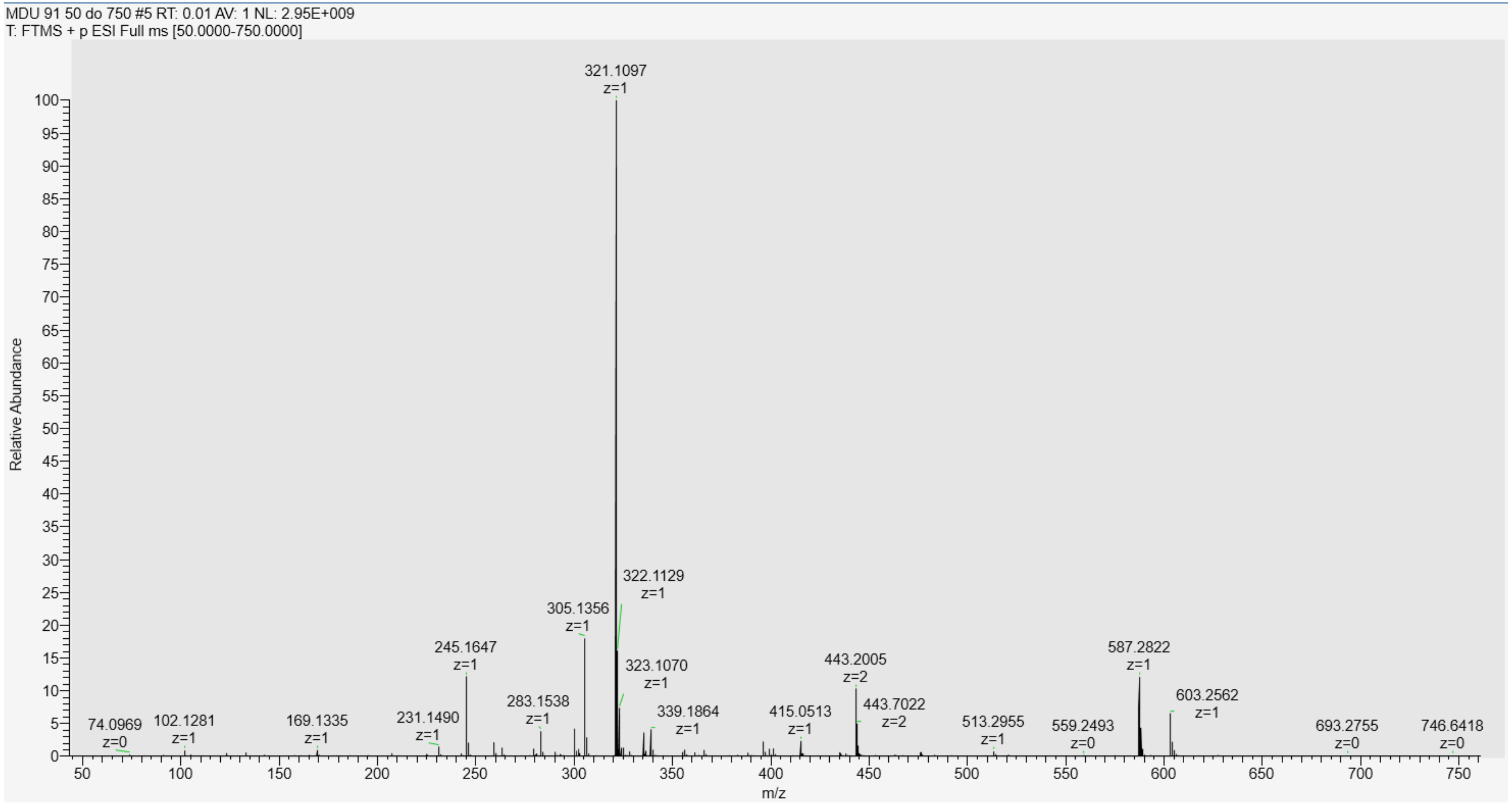


**Figure S44.** HRMS spectrum of compound **13**.

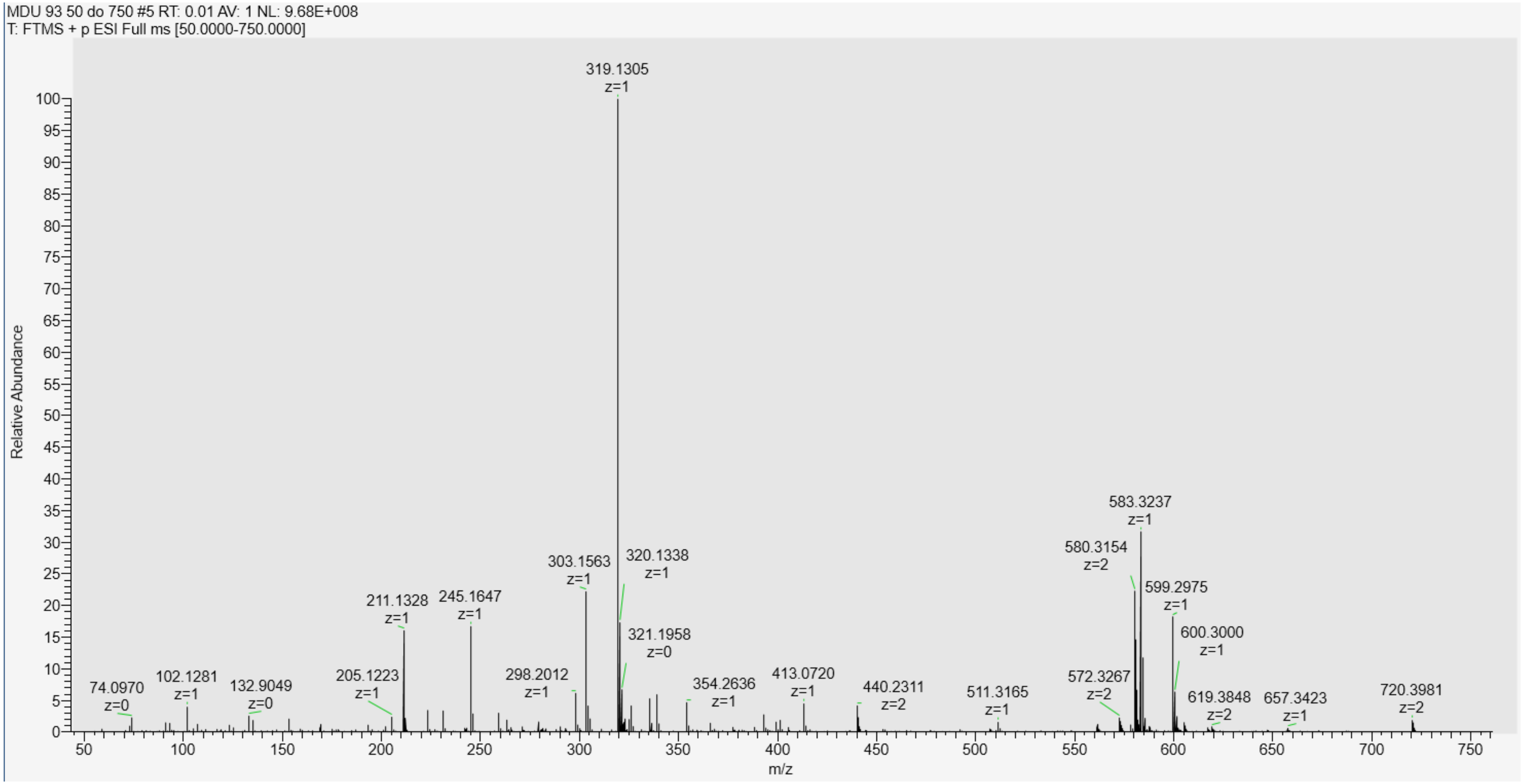


**Figure S45.** HRMS spectrum of compound **15**.

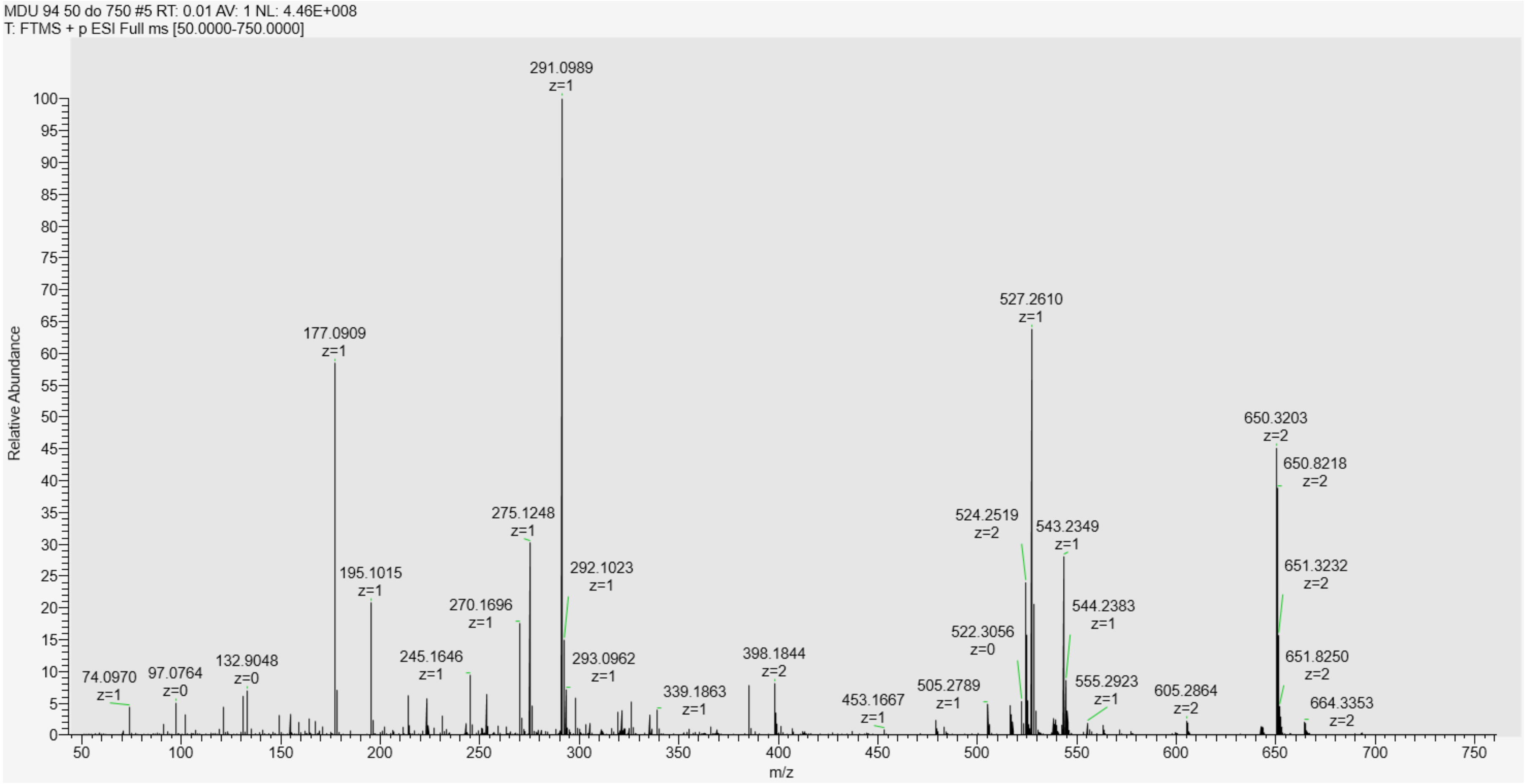


**Figure S46.** HRMS spectrum of compound **16**.

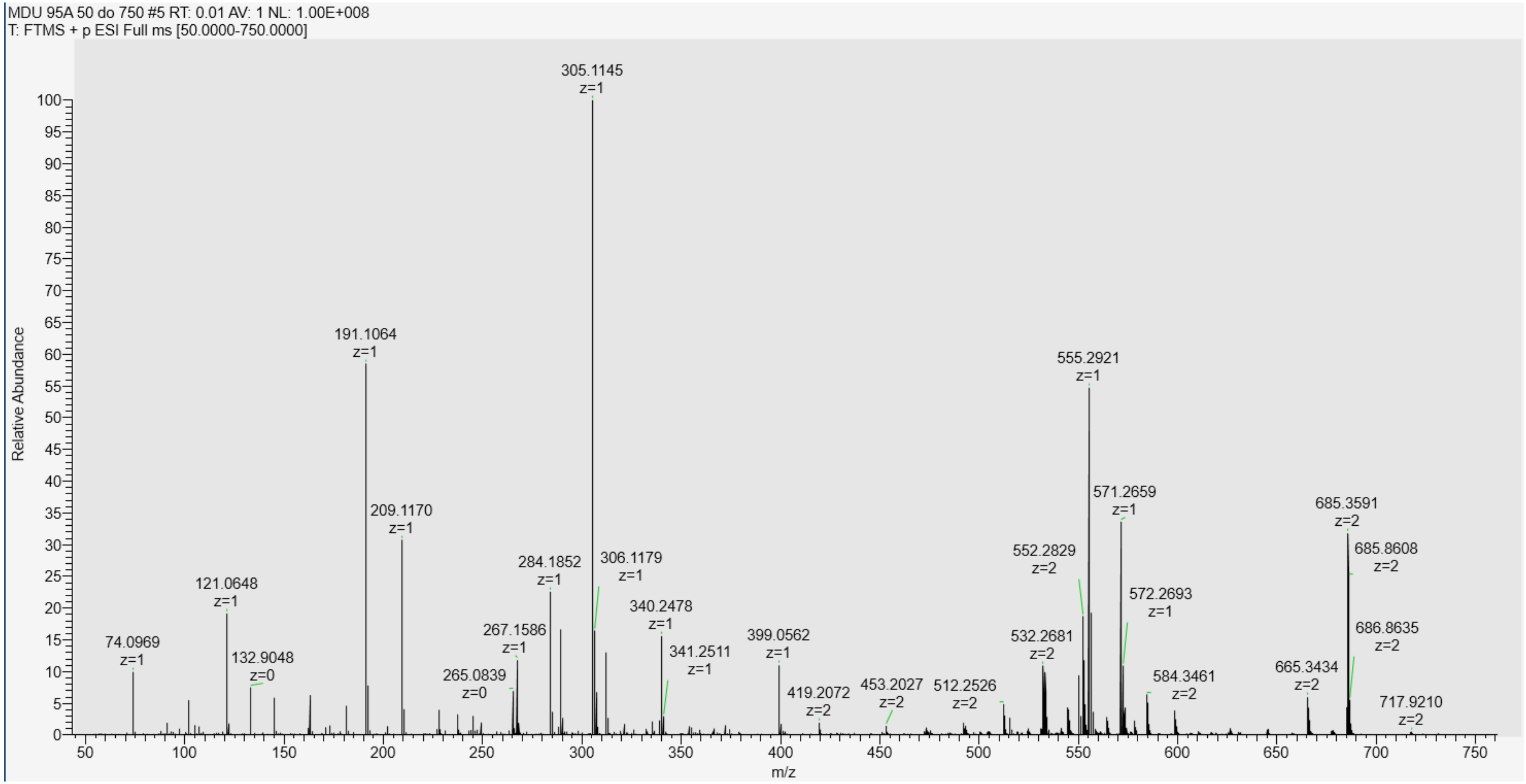


**Figure S47.** HRMS spectrum of compound **17**.

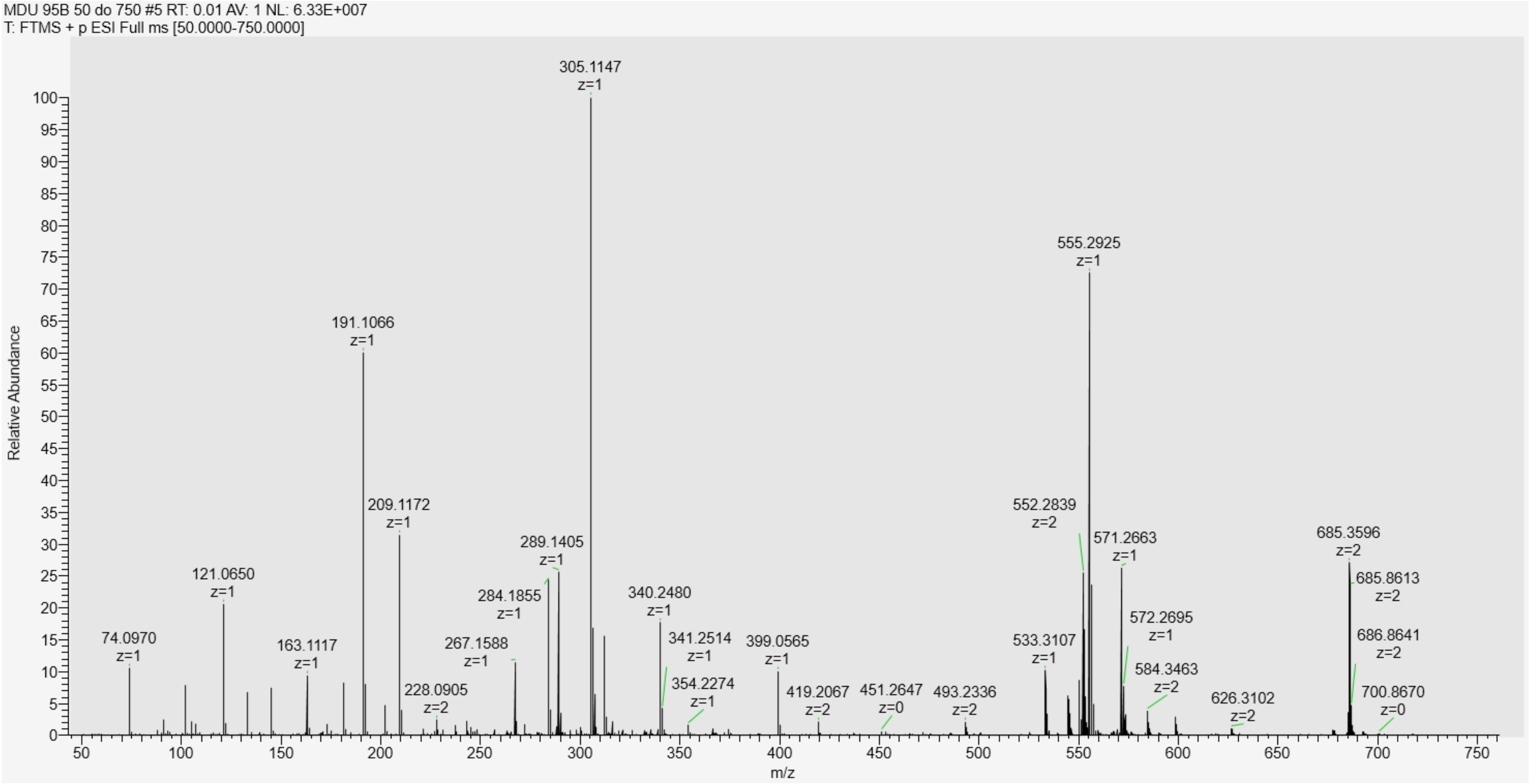


**Figure S48.** HRMS spectrum of compound **18**.

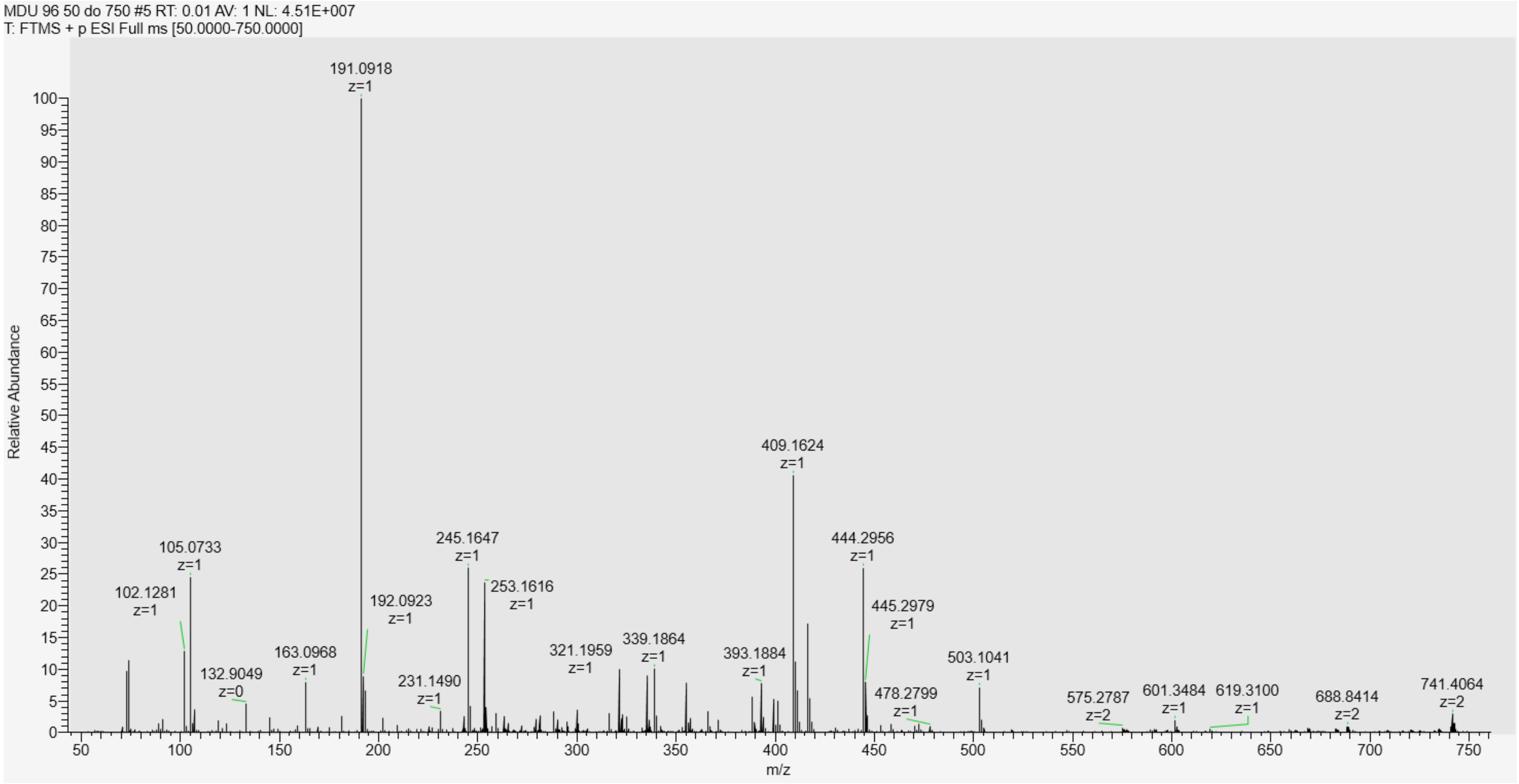


**Figure S49.** HRMS spectrum of compound **19**.

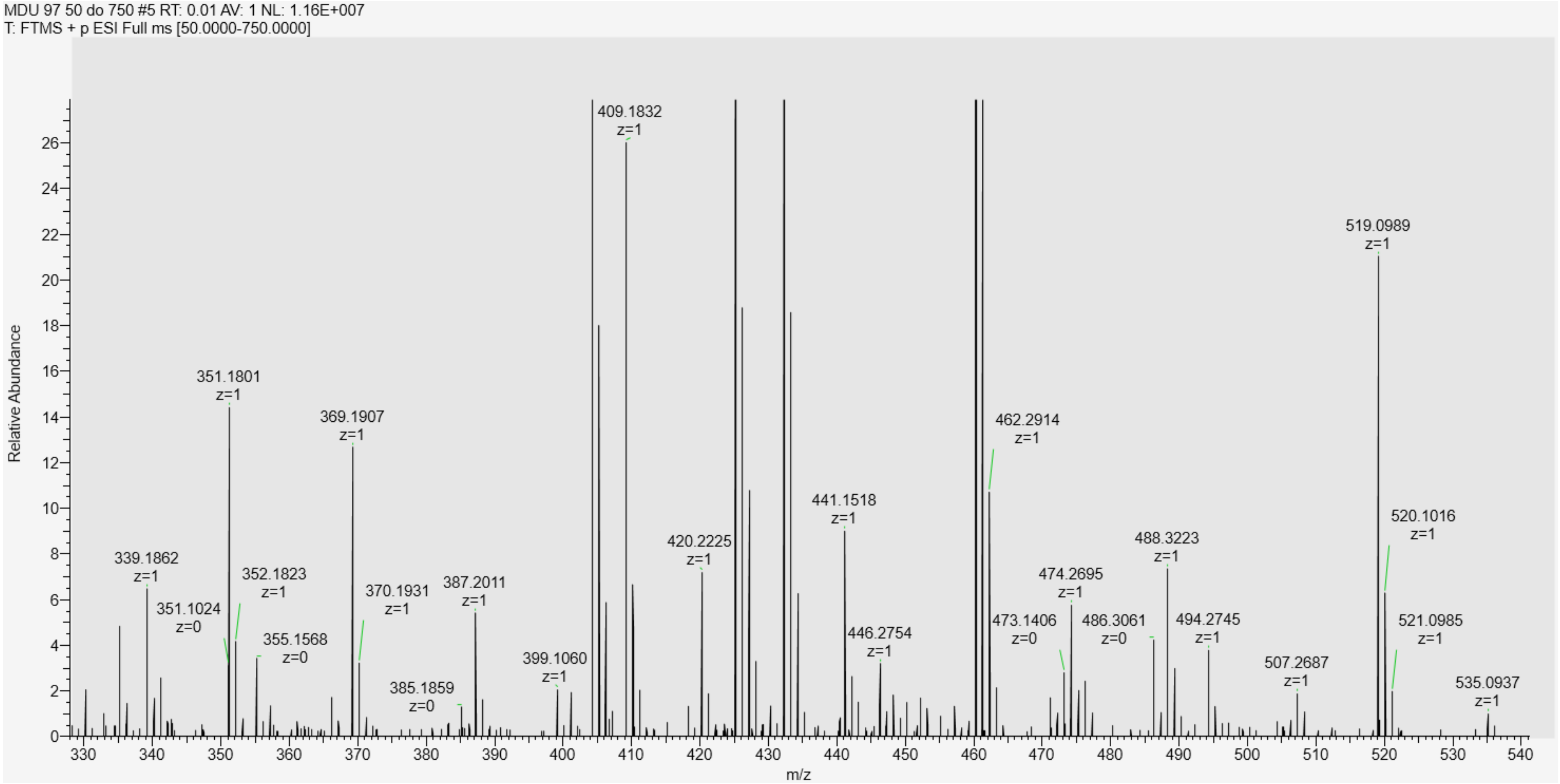


**Figure S50.** HRMS spectrum of compound **20**.

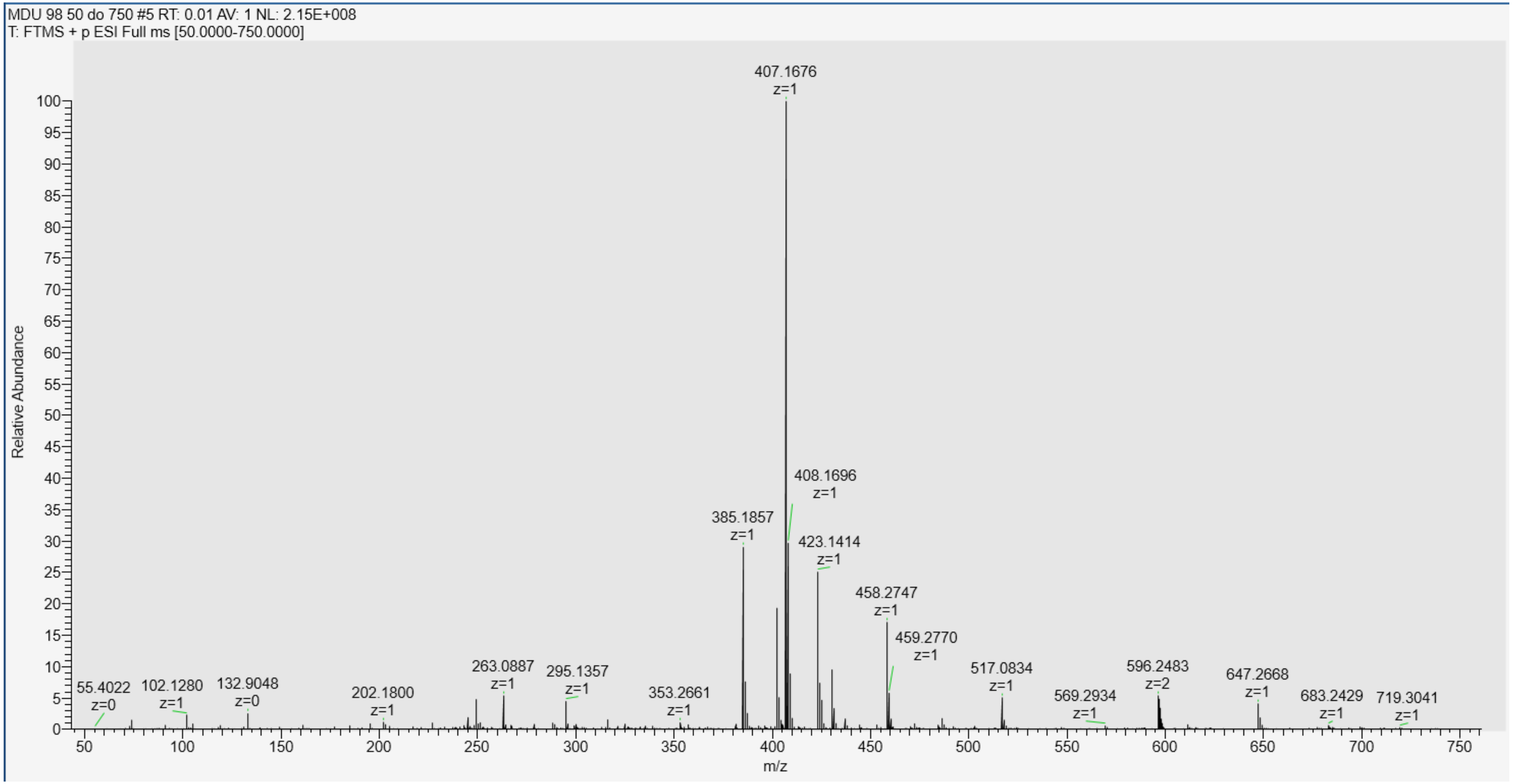


**Figure S51.** HRMS spectrum of compound **21**.

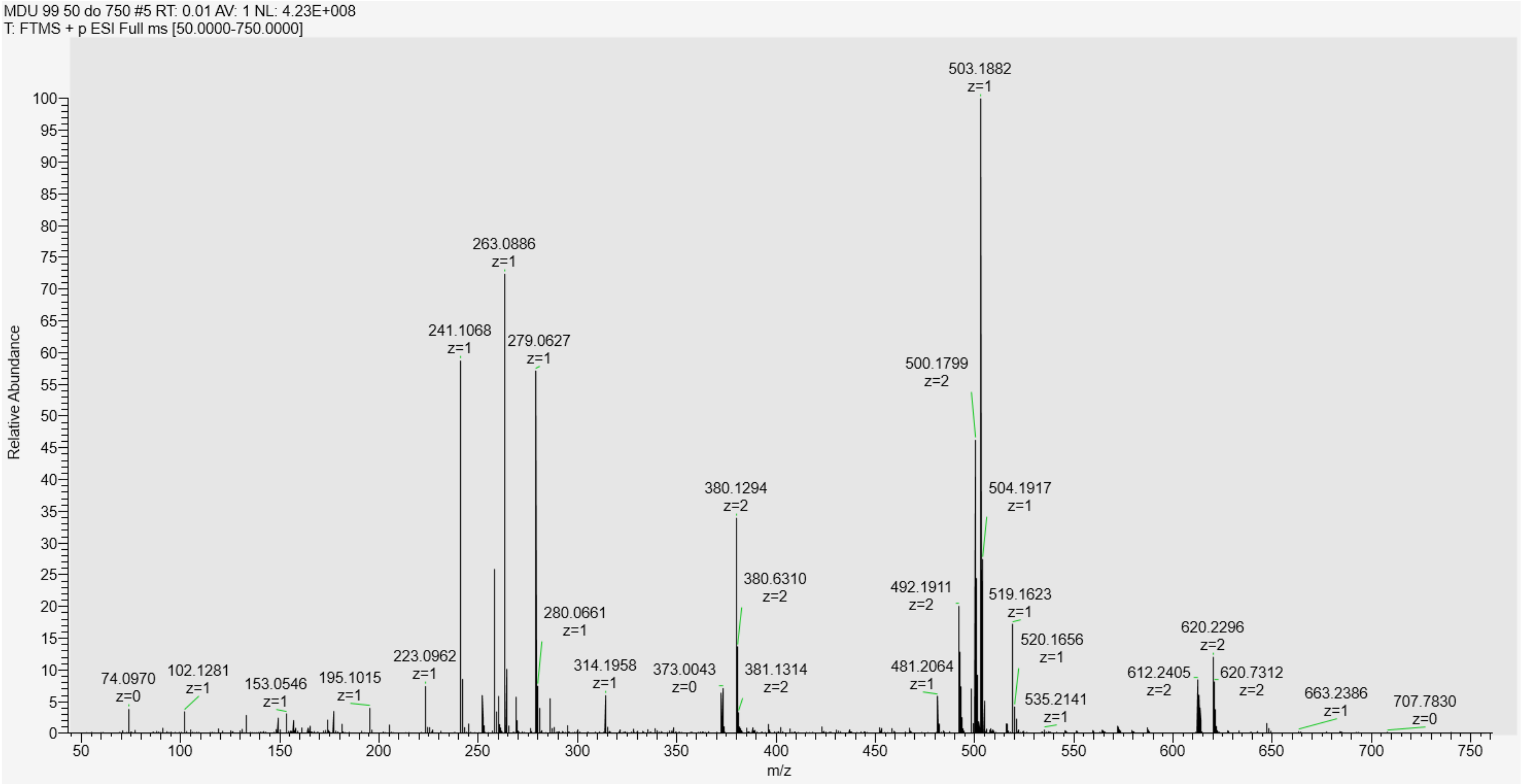


**Figure S52.** HRMS spectrum of compound (+)-**1**.

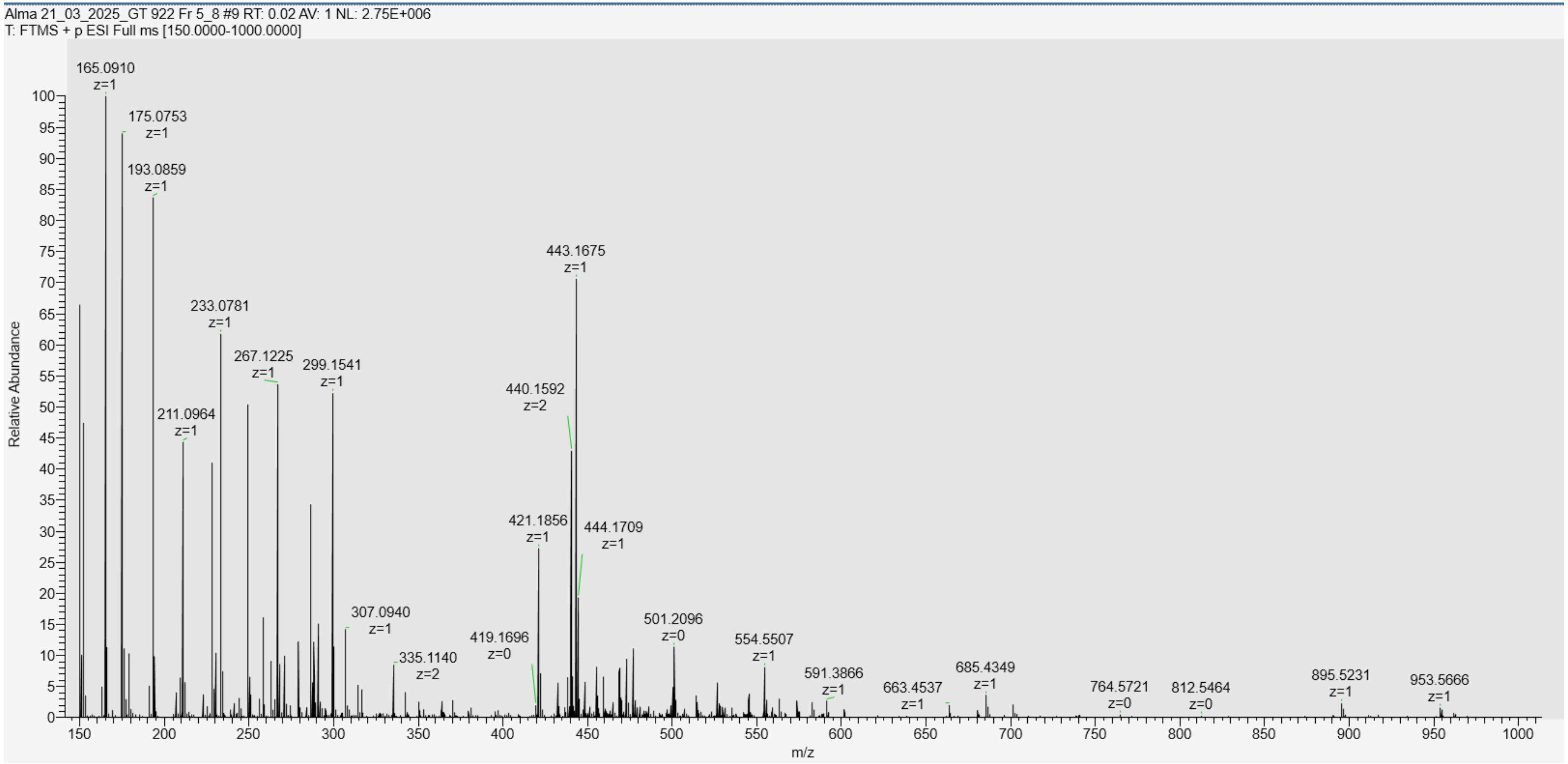


**Figure S53.** HRMS spectrum of compound **22**.

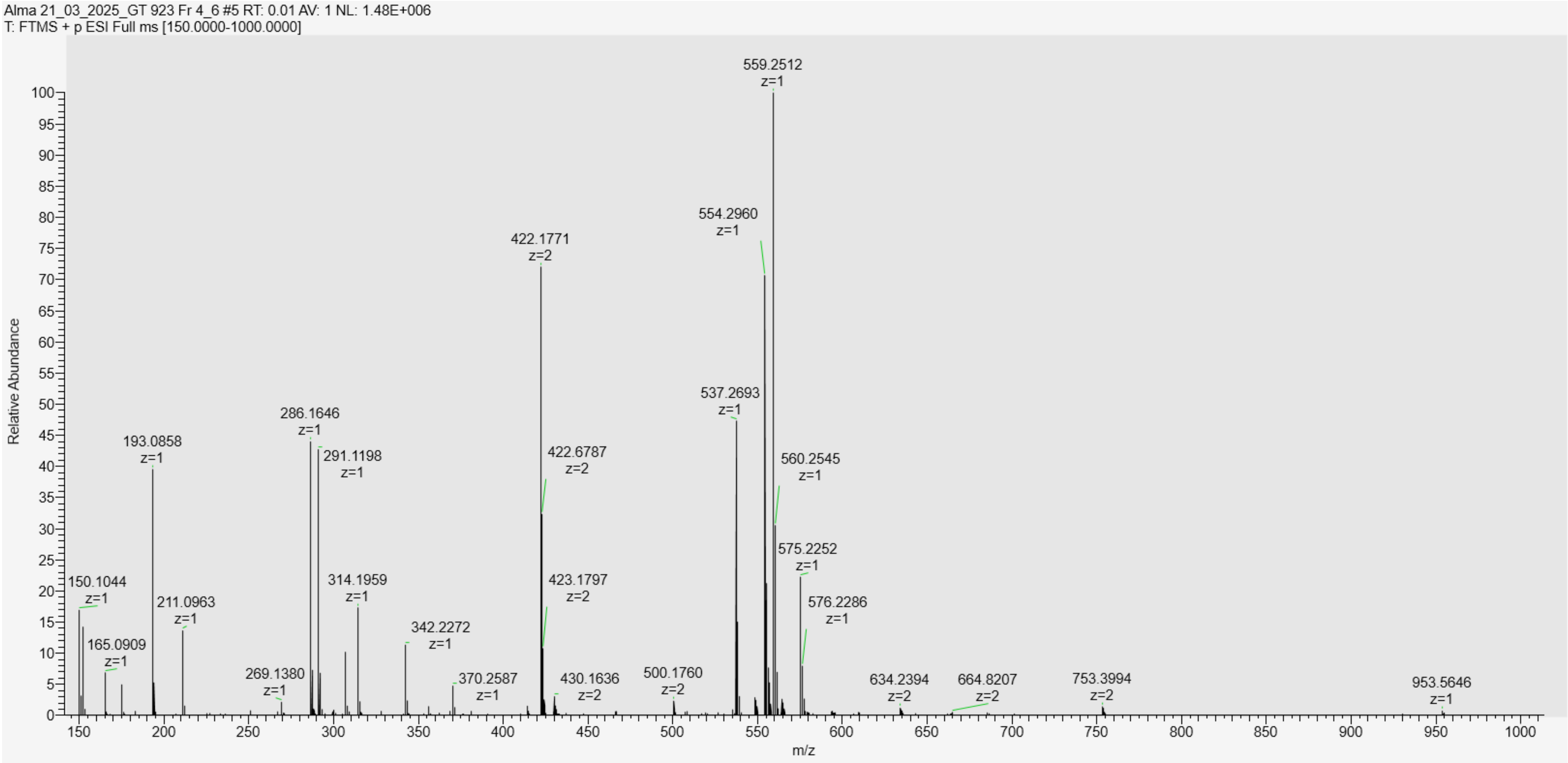


**Figure S54.** HRMS spectrum of compound **23**.

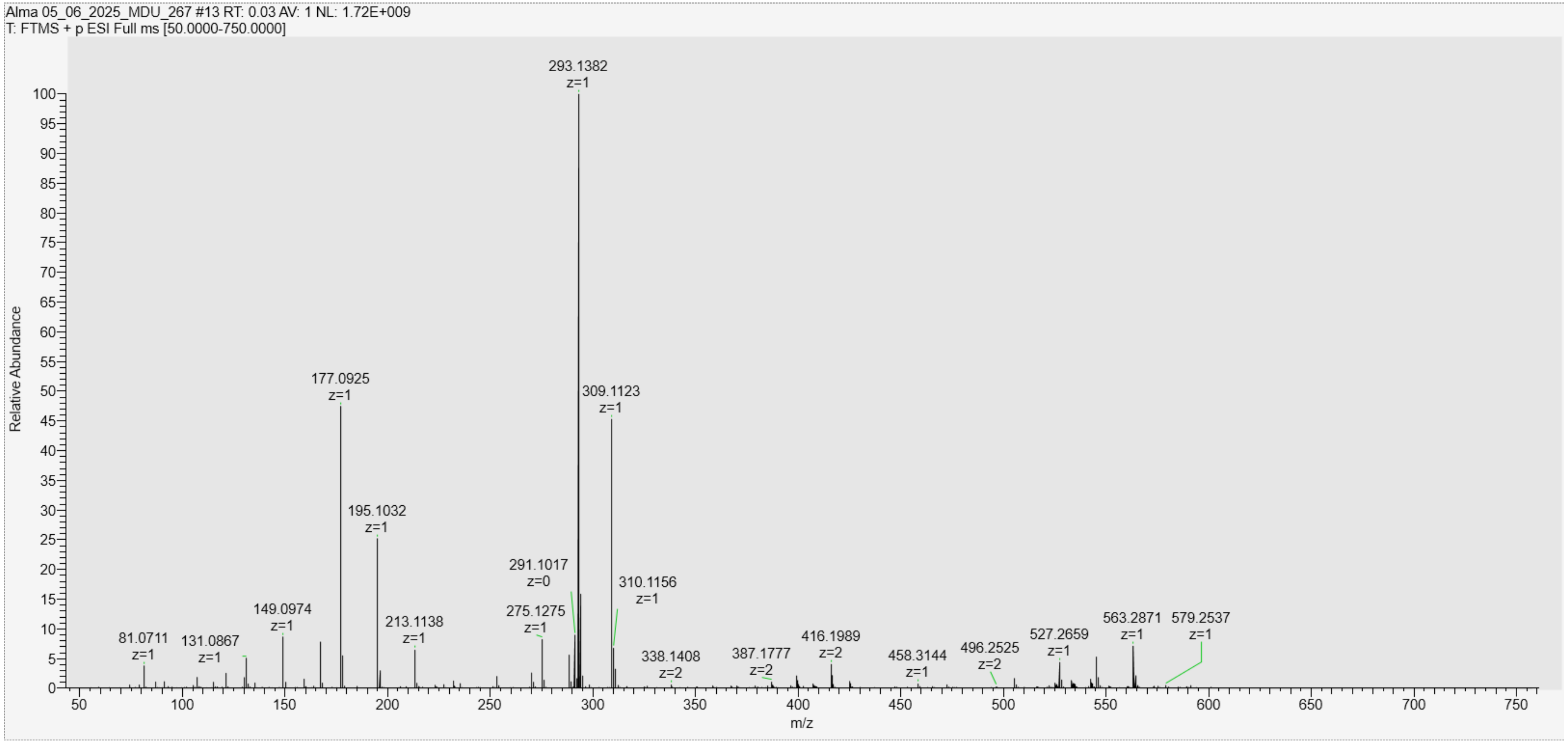


**Figure S55.** HRMS spectrum of compound **24**.
